# Developmental dynamics of chromatin accessibility during post-implantation development of monkey embryos

**DOI:** 10.1093/gigascience/giad038

**Published:** 2023-05-25

**Authors:** Xi Dai, Honglian Shao, Nianqin Sun, Baiquan Ci, Jun Wu, Chuanyu Liu, Liang Wu, Yue Yuan, Xiaoyu Wei, Huanming Yang, Longqi Liu, Weizhi Ji, Bing Bai, Zhouchun Shang, Tao Tan

**Affiliations:** College of Life Sciences, University of Chinese Academy of Sciences, Beijing 100049, China; BGI-Shenzhen, Shenzhen 518083, China; State Key Laboratory of Primate Biomedical Research; Institute of Primate Translational Medicine, Kunming University of Science and Technology, Kunming, Yunnan 650500, China; Yunnan Key Laboratory of Primate Biomedical Research, Kunming, Yunnan 650500, China; State Key Laboratory of Primate Biomedical Research; Institute of Primate Translational Medicine, Kunming University of Science and Technology, Kunming, Yunnan 650500, China; Yunnan Key Laboratory of Primate Biomedical Research, Kunming, Yunnan 650500, China; State Key Laboratory of Primate Biomedical Research; Institute of Primate Translational Medicine, Kunming University of Science and Technology, Kunming, Yunnan 650500, China; Yunnan Key Laboratory of Primate Biomedical Research, Kunming, Yunnan 650500, China; Department of Molecular Biology, University of Texas Southwestern Medical Center, Dallas, TX 75390, USA; BGI-Shenzhen, Shenzhen 518083, China; College of Life Sciences, University of Chinese Academy of Sciences, Beijing 100049, China; BGI-Shenzhen, Shenzhen 518083, China; BGI-Shenzhen, Shenzhen 518083, China; BGI-Shenzhen, Shenzhen 518083, China; James D. Watson Institute of Genome Sciences, Hangzhou 310013, China; College of Life Sciences, University of Chinese Academy of Sciences, Beijing 100049, China; BGI-Shenzhen, Shenzhen 518083, China; State Key Laboratory of Primate Biomedical Research; Institute of Primate Translational Medicine, Kunming University of Science and Technology, Kunming, Yunnan 650500, China; Yunnan Key Laboratory of Primate Biomedical Research, Kunming, Yunnan 650500, China; State Key Laboratory of Primate Biomedical Research; Institute of Primate Translational Medicine, Kunming University of Science and Technology, Kunming, Yunnan 650500, China; Yunnan Key Laboratory of Primate Biomedical Research, Kunming, Yunnan 650500, China; College of Life Sciences, University of Chinese Academy of Sciences, Beijing 100049, China; BGI-Shenzhen, Shenzhen 518083, China; James D. Watson Institute of Genome Sciences, Hangzhou 310013, China; State Key Laboratory of Primate Biomedical Research; Institute of Primate Translational Medicine, Kunming University of Science and Technology, Kunming, Yunnan 650500, China; Yunnan Key Laboratory of Primate Biomedical Research, Kunming, Yunnan 650500, China

**Keywords:** cynomolgus monkey, ex vivo, gastrulation, scATAC-seq, chromatin dynamics

## Abstract

**Background:**

Early post-implantation development, especially gastrulation in primates, is accompanied by extensive drastic chromatin reorganization, which remains largely elusive.

**Results:**

To delineate the global chromatin landscape and understand the molecular dynamics during this period, a single-cell assay for transposase accessible chromatin sequencing (scATAC-seq) was applied to *in vitro* cultured cynomolgus monkey (*Macaca fascicularis*, hereafter referred to as monkey) embryos to investigate the chromatin status. First, we delineated the *cis*-regulatory interactions and identified the regulatory networks and critical transcription factors involved in the epiblast (EPI), hypoblast, and trophectoderm/trophoblast (TE) lineage specification. Second, we observed that the chromatin opening of some genome regions preceded the gene expression during EPI and trophoblast specification. Third, we identified the opposing roles of FGF and BMP signaling in pluripotency regulation during EPI specification. Finally, we revealed the similarity between EPI and TE in gene expression profiles and demonstrated that PATZ1 and NR2F2 were involved in EPI and trophoblast specification during monkey post-implantation development.

**Conclusions:**

Our findings provide a useful resource and insights into dissecting the transcriptional regulatory machinery during primate post-implantation development.

## Background

The transition from pre-implantation to gastrulation represents a milestone of early embryogenesis in primates and involves extensive morphogenesis and lineage specification and differentiation. During this stage, a connection between the embryo and the mother is established, while the trophectoderm (TE) differentiates into cytotrophoblasts (CTs), extravillous cytotrophoblasts (EVTs), and syncytiotrophoblasts; the cavitation of the amnion and yolk sac initiates, and the gastrulation of the embryo launches to form 3 germ layers and program the body plan of the fetus [1, 2]. However, there are technical limitations and ethical concerns, and the molecular mechanisms underlying this transition remain largely elusive.

Recently, advancements in embryo *in vitro* culture systems have enabled us to investigate transcriptional and DNA methylation dynamics during the early embryonic development in humans and monkeys [3–7]. However, several key questions, including the chromatin status that underlies this transition, have yet to be addressed.

In the mouse, chromatin accessibility, histone modifications, and 3-dimensional chromatin structures during post-implantation development have been extensively studied, and epigenetic regulatory networks have been revealed [8–13]. As significant differences exist between primates and mice in terms of post-implantation development, for example, in the morphogenesis of embryonic and extra-embryonic structures and signaling pathways involved in the specification of embryonic and extra-embryonic lineages [1, 2, 14, 15], the knowledge derived from mouse models could not be straightforwardly extrapolated to primate models. This poses a significant limitation to studies of, for example, the regulation of pluripotent stem cells (PSCs) in primates.

Here, we harness the power of a single-cell assay for transposase accessible chromatin sequencing (scATAC-seq) and embryo *in vitro* culture platform to unravel the regulatory chromatin landscape during early embryonic development in monkeys. This study provides a valuable resource for studying chromatin dynamics and chromatin regulation during early embryonic development in primates.

## Results

### scATAC-seq profiles of monkey early embryogenesis

**Figure 1:**
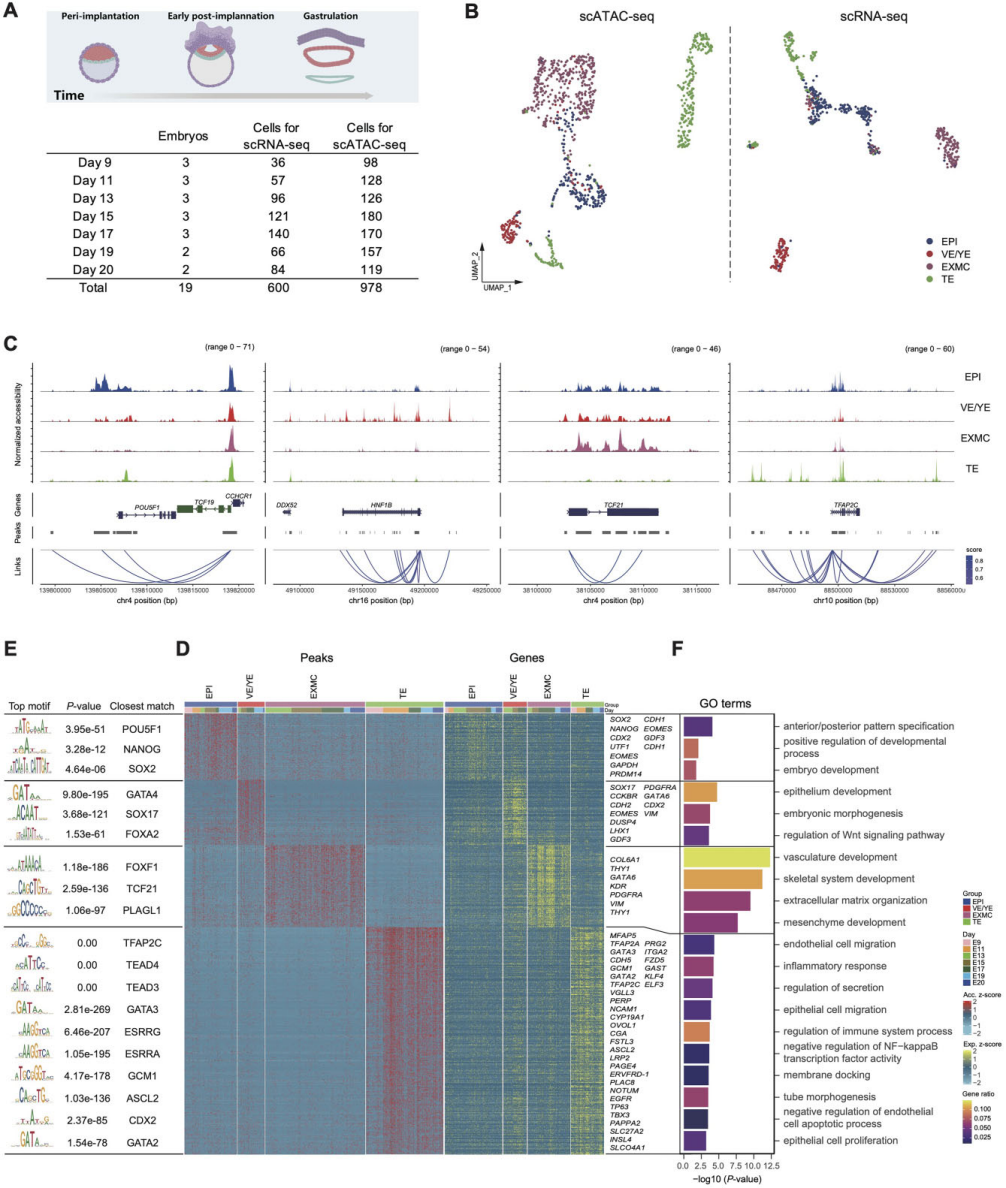
Landscape of chromatin accessibility during monkey peri- and post-implantation development. (A) Schematic illustration of scATAC sequencing of monkey embryos at different developmental stages. (B) UMAP plot of all the scATAC-seq and scRNA-seq cells. Cells are colored by their cell-type annotation. ATAC cell types were transferred from RNA. (C) Aggregated scATAC-seq tracks denoting the chromatin accessibility peaks for the marker genes of each cell type. Peak-to-gene linkages are shown at the bottom, and correlations are represented by arcs colored by the correlation score (color scales for both panels are to the right). (D) Heatmap showing DPs and corresponding DEGs for each cell type and some well-studied lineage markers are listed. Each row shows one DP with its corresponding gene, whereas some genes are duplicated in a row cluster as multiple peaks link to one gene. (E) Representative lineage-specific TFs and their binding motifs in DPs. (F) Representative enriched GO terms within each lineage. *P* values derived from the hypergeometric test are shown, and the color indicates the gene ratio.

To determine the regulatory landscape at single-cell resolution during monkey peri- and post-implantation development, we performed scATAC-seq of cultured monkey embryos from day 9 post-fertilization (9 d.p.f.) to 20 d.p.f. as our previously reported and published single-cell RNA sequencing (scRNA-seq) dataset was included for analysis [5] (Fig. 1A and Supplementary Fig. S1A). In total, 1,198 individual cells were sequenced, and after stringent filtration (usable fragments >10,000, promoter fragments ratio >10%), 978 high-quality single nuclei, distributing from 9 d.p.f. and 20 d.p.f., were retained (Supplementary Table S1 and Supplementary Fig. S1A). Cells within per embryo passing filter had median fragments ranging from 20,020 to 61,182, the median fraction of fragments in promoters (500 bp around transcriptional start site) ranged from 12.14% to 19.01%, and the median fraction of fragments in peaks ranged from 51.37% to 64.39% (Supplementary Fig. S1B).

Next, based on these high-quality data, we investigated global gene regulatory activities during monkey early development. First, the resting 978 cells were dimensionally reduced using uniform manifold approximation and projection (UMAP) and clustering analysis. In scRNA-seq analysis, four main cell clusters—namely, epiblast (EPI), TE, visceral endoderm or yolk-sac endoderm (VE/YE), and extra-embryonic mesenchyme cell (EXMC)—were identified (Fig.   1B). To interrogate correspondence between chromatin accessibility and gene expression during early monkey embryogenesis, we then integrated our scATAC-seq and scRNA-seq datasets. Generally, canonical correlation analysis (CCA) and mutual nearest-neighbor (MNN) algorithms were applied, and the annotated scRNA-seq dataset was used as a reference to annotate the scATAC-seq dataset (see also Methods). This integrated object yielded consistent overlap between scRNA-seq and scATAC-seq cell types with high integration scores after coembedding scRNA-seq and scATAC-seq datasets (Supplementary Fig. S1C and S1D). Additionally, the cell proportion (Supplementary Fig. S1E) and the gene expression and chromatin accessibility of lineage markers were comparable (Supplementary Fig. S1F). These results suggest a strong correlation between chromatin accessibility and gene expression in this integrated analysis.

Next, enrichment of ATAC-seq peaks in promoter and distal regions of lineage markers was identified, including the *OCT4* locus (also known as *POU5F1*) in the EPI, *TFAP2C* in the TE, *HNF1B* in the VE/YE, and *TCF21* in the EXMCs (Fig. 1C). Furthermore, the binding motifs and the expression of these markers were also enriched in the four major cell clusters (Supplementary Fig. S1G).

We then related the cluster-specific differential peaks (DPs) to the differentially expressed genes (DEGs) (Supplementary Table S2 and Fig. 1D). Furthermore, the enrichment of well-known lineage-specific transcription factor (TF) binding motifs was observed for cluster-specific DPs, such as *OCT4* (*POU5F1*) and *NANOG* in the EPI, *TFAP2C* and *TEAD4* in the TE, *GATA4* in the VE/YE, and *TCF21* and *FOXF1* in EXMCs (Fig. 1E). Gene Ontology (GO) term enrichment analysis of DEG-related DPs revealed that the early-development associated terms such as anterior/posterior pattern specification and embryo development were enriched in the EPI; the most enriched GO terms in the VE/YE included epithelium development and regulation of WNT signaling. According to the cell identity of EXMCs, mesenchyme development-associated terms were enriched in this cell lineage. Interestingly, we observed inflammatory response, regulation of immune system processes, and other GO terms enriched in the TE cells, suggesting their potential role in immune regulation during pregnancy [16] (Fig. 1F). Taken together, these findings indicate that combining an embryo *in vitro* culture platform with powerful scATAC-seq can successfully generate comprehensive and high-quality maps of open chromatin and lineage regulators during early monkey embryogenesis.

### Lineage-specific transcriptional regulatory networks of monkey early embryonic development

**Figure 2:**
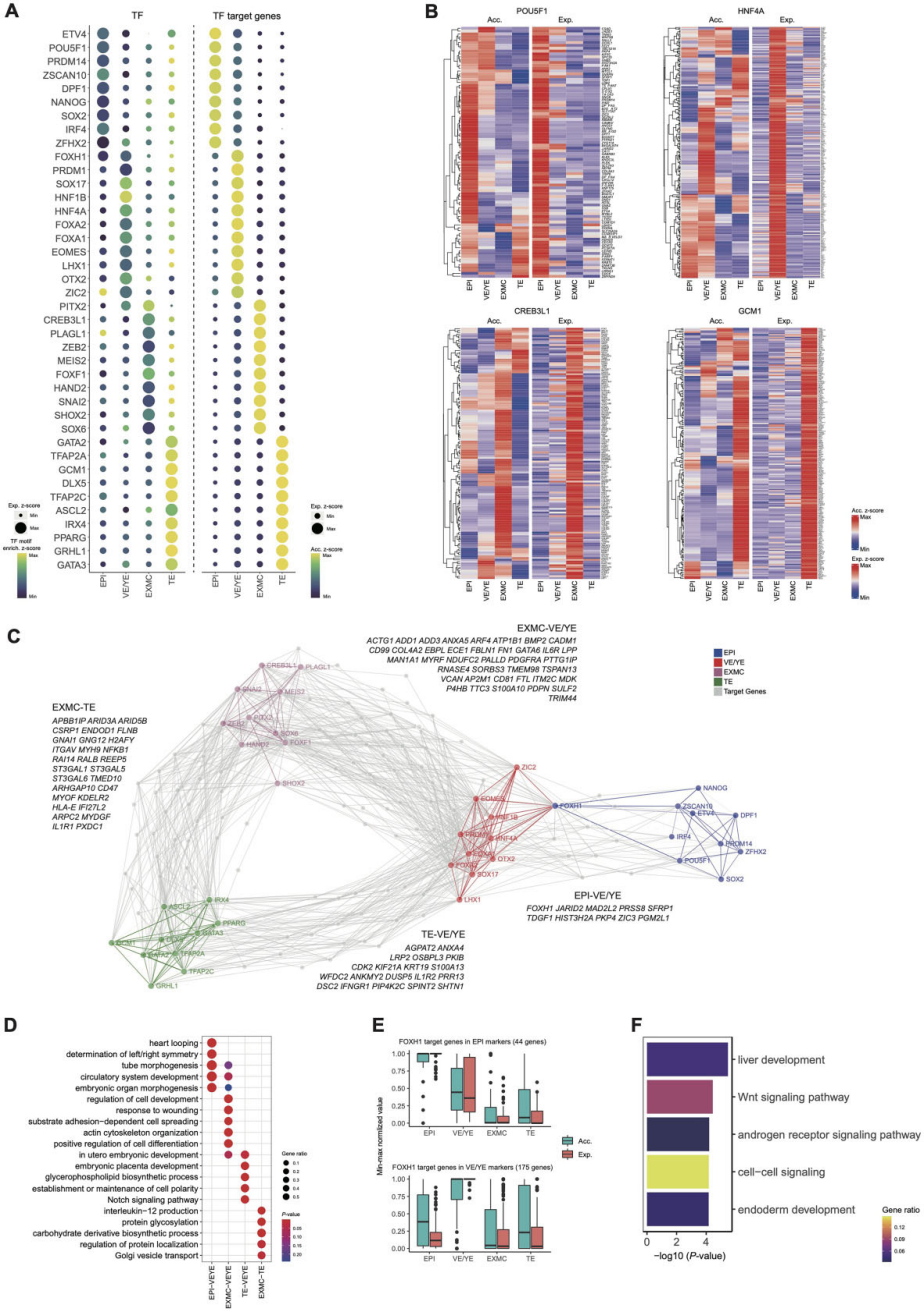
TF regulatory networks of early monkey embryogenesis. (A) The *z*-scores of averaged lineage-specific TF expression and corresponding motif enrichment in the DPs of each lineage (left panel). The *z*-scores of averaged lineage-specific TF target genes expression levels and averaged gene activity scores calculated from cells aggregated by lineage (right panel). Only the top ten lineage-specific TFs of each lineage with the most significant *P* values in the DEGs are shown here. (B) Heatmaps showing the averaged gene expression levels and activity scores of target genes of representative lineage-specific TFs. (C) Lineage-specific TF regulatory networks and target genes regulated by multiple lineage-specific TF modules are shown. The representative target genes are listed beside. (D) The corresponding genes' top five GO enrichment terms are listed in (C). (E) *FOXH1* target gene activity scores and expression levels in the EPI and VE/YE lineages, respectively. All values were min–max normalized. (F) Representative GO enrichment terms of *FOXH1* target genes. *P* values derived from the hypergeometric test are shown, and the color indicates the gene ratio.

To further characterize the transcriptional regulatory networks of monkey early embryonic development, we determined the lineage-specific TFs and their enriched motifs, as well as lineage-specific DPs. In addition, the gene activity scores and expression levels of the TF target genes were analyzed. As a result, a series of TFs, which may play important roles in cell lineage specification, were identified (Supplementary Table S3). The gene expression levels, TF motif enrichment, and chromatin accessibility of the top ten lineage-specific TFs and their target genes are shown in Fig.   2A and B. Next, leveraging identified lineage-specific TFs and their target genes, and we constructed modules of lineage-specific TFs and regulatory networks of target genes that were putatively coregulated by two lineage-specific TF modules (EPI-VE/YE, EXMC-VE/YE, EXMC-TE, and TE-VE/YE). EPI and VE/YE lineages were highly related in the networks by hub TFs, such as *FOXH1*, indicating their similarities in the regulatory program during early embryogenesis. In contrast, TE and EXMC lineages were distinct from the other lineages (Fig. 2C). Also, the top five GO terms of TF target genes are shown in Fig.   2D. Consistent with the transcriptional regulatory network analysis, we observed that the genome regions of TF coregulated genes were more accessible in coregulated lineages. Notably, the expression of TF coregulated genes displayed a lineage-specific pattern, suggesting that besides chromatin accessibility, additional mechanisms exist to guarantee lineage specification during early embryogenesis. For example, the expression and chromatin accessibility levels of *FOXH1* target genes in the EPI and VE/YE (Fig. 2E), as these genes are both involved in the WNT signaling pathway in the EPI and VE/YE (Fig. 2F).

### Single-cell chromatin accessibility reveals regulatory mechanisms of EPI lineage

**Figure 3:**
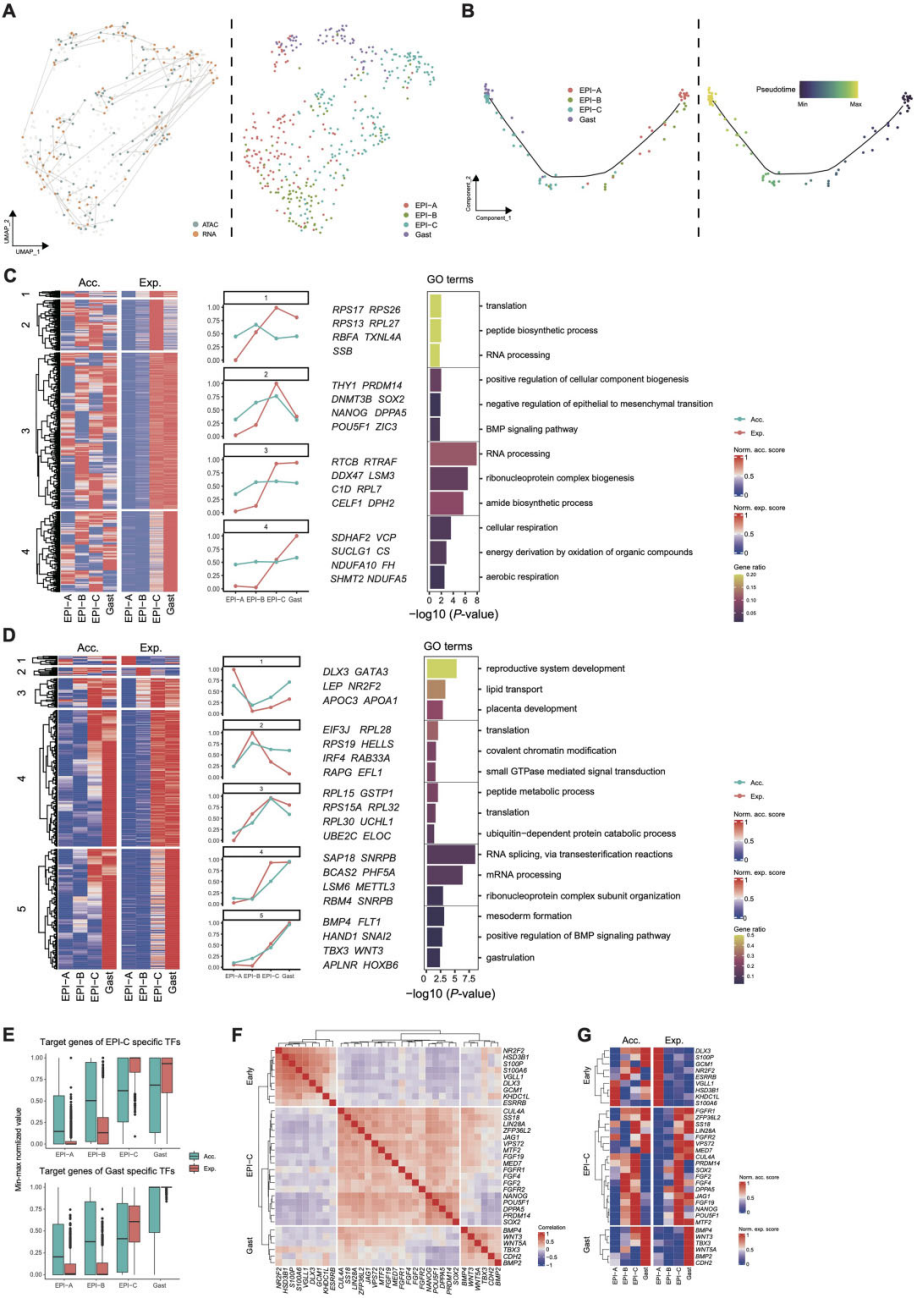
Transcriptional regulation of EPI specification. (A) Coembedded UMAP for single-cell pairs of scATAC-seq and scRNA-seq datasets based on geodesic distance-based pairing approach. The colors of cells represent technology and cell type. (B) Pseudotime trajectory of the scATAC-scRNA paired EPI subtypes. Cells are colored by the EPI subtypes and pseudotime. (C) Heatmaps showing gene activity scores and expression levels of subtype-specific DEGs in pattern 1, which are classified into four clusters based on hierarchical clustering. All values were min–max normalized (left panel). Line chart showing the averaged gene activity scores and gene expression levels in heatmap clusters, with representative genes listed (middle panel). Bar chart showing the representative GO enrichment terms of genes in heatmap clusters. *P* values derived from the hypergeometric test are shown, and the color indicates the gene ratio (right panel). (D) Heatmaps showing gene activity scores and expression levels of subtype-specific DEGs in pattern 2, which are classified into five clusters based on hierarchical clustering, and all values were min–max normalized (left panel). Line chart showing the averaged gene activity scores and gene expression levels in heatmap clusters, with representative genes listed (middle panel). Bar chart showing the representative GO enrichment terms of genes in heatmap clusters. *P* values derived from the hypergeometric test are shown, and the color indicates the gene ratio (right panel). (E) Box chart of averaged EPI-C and gastrulating cell (Gast)–specific TF target gene activity scores and expression levels. (F) Heatmap showing the correlation coefficients of gene expression levels among the representative genes. These genes were classified into three groups by hierarchical clustering. They were upregulated in early EPI (EPI-A and EPI-B), EPI-C, and Gast, respectively. (G) Heatmaps showing gene activity scores and gene expression levels corresponding to (F), and all values were min-max normalized.

To explore the regulatory mechanisms underpinnings the specification of EPI cells, Seurat [17] and FigR [18] were first applied to integrate the scRNA-seq and scATAC-seq datasets. We observed that most of the cells in the scRNA-seq and scATAC-seq datasets overlapped with each other, suggesting that the chromatin accessibility and gene expression in most EPI cells occur in a concordant manner during early embryonic development (Fig. 3A). The coembedded UMAP plot identified four cell clusters, and they were designated as EPI-A, EPI-B, EPI-C, and gastrulating cell (Gast) (Fig.   3A) based on our previous study [5]. Next, the Monocle2 analysis [19] was applied to construct the developmental trajectory of EPI-A, -B, -C, and Gast cells. We observed that all the cells were ordered in a U-like trajectory, with EPI-A cells occupying one end and Gast cells occupying the other. EPI-B and -C cells were in the middle (Fig. 3B). Worth noting is that identical developmental trajectories were obtained when different algorithms were applied, such as Slingshot [20] and TSCAN [21] (Supplementary Fig. S2A). This trajectory revealed the continuous differentiation of EPI-A to Gast cells, as EPI-A cells are equivalent to ICM cells, EPI-B cells are equivalent to EPI cells at the pre-implantation stage (EPI-A and -B were subsequently renamed as early EPI ), and EPI-C cells are equivalent to EPI cells at the early post-implantation stage. Gast cells are equivalent to *in vivo* gastrulation cells (Supplementary Fig. S2B).

To delineate the regulatory role of chromatin accessibility in EPI specification, we examined the cell type–specific DPs from EPI-A to Gast cells. During the transition from EPI-A to B and EPI-C to Gast, most genome regions tended to gain accessibility. In contrast, they lost accessibility during the transition from EPI-B to -C (Supplementary Fig. S2C and Table S4). Notably, *Hox* gene activation was observed when the EPI cells underwent gastrulation, indicating their important roles in primitive streak formation (Supplementary Fig. S2D). Interestingly, we observed that the activation of chromatin states preceded the expression of genes in some genome regions (Supplementary Fig. S2E), which implies an inconsistency between gene expression and chromatin accessibility during EPI cell specification. The GO terms and TF binding motif enrichment analyses of DPs indicated that *OTC4* and *SOX2* might be involved in the transition from EPI-A to -B and early post-implantation stage EPI (EPI-C) to gastrulating cells (Gast) (Supplementary Fig. S2E and 2F).

To interrogate the correlation between gene expression and chromatin accessibility, we related the DPs to the DEGs, and two main patterns were detected: (i) chromatin became accessible first in EPI-A cells, and then the genes were expressed (pattern 1) (Fig.   3C), and (ii) the scaled values of gene expression and chromatin accessibility levels were comparable in EPI-A cells (Fig. 3D) (pattern 2). In pattern 1, 4 subpatterns (clusters 1–4) were identified, and the GO enrichment analysis was performed. For example, pluripotency-related TFs, such as *OCT4* , *SOX2*, and *NANOG*, belong to cluster 2, in which chromatin opens at pre- and peri-implantation stages (EPI-A and EPI-B, early-stage EPI), and gene expressions were upregulated until the post-implantation stage (EPI-C) (Fig.   3C), which was also observed during the establishment of mouse PSCs in different pluripotent states [22].

In pattern 2, 5 subpatterns (clusters 1–5) were observed. Interestingly, genes involved in mesoderm formation and gastrulation belonged to pattern 2 (Fig. 3D). To further determine the dynamics of gene expression and chromatin accessibility during EPI specification, Monocle2-based pseudotime ordering of aligned single scRNA-seq and scATAC-seq cells was performed, and gene expression and chromatin accessibility levels were investigated. Similarly, two main patterns were observed: (i) the opening of chromatin regions preceded the gene expression, and (ii) the opening of chromatin regions occurred concurrently with the gene expression, despite subpatterns being detected in these 2 main patterns (Supplementary Fig. S3A).

As little is known about the pluripotency regulation of post-implantation EPI cells, we next focused our analysis on EPI-C and Gast cells. The EPI-C- and Gast-specific TFs (Supplementary Table S5), chromatin accessibility, and the expression levels of TF target genes were determined. We observed that chromatin became accessible preceding the gene expression, indicative of an initial priming process of post-implantation EPI cells before their commitment to the specific lineage (Fig. 3E). Monocle2-based pseudotime ordering analysis of aligned single scRNA-seq and scATAC-seq cells further confirmed this observation (Supplementary Fig. S3B). We then investigated mechanisms leading to the pluripotency transition from early EPI to Gast. We observed that the expressions of core pluripotent factors, such as *OCT4* and *NANOG*, were upregulated in EPI-C cells, which highly correlated with the expression of FGF signaling members such as *FGF2, FGF4*, and FGF receptor 1 (*FGFR1* ) (Fig.   3F). Furthermore, the expression of gastrulation marker genes, including *TBX3* and *CDH2*, was highly correlated with the expression of BMP and WNT signaling members. Neither FGF nor BMP signaling could regulate the expressions of naive pluripotency-related genes [23] (Fig. 3F), and the activation of chromatin regions corresponding to FGF and BMP signaling members preceded their expression (Fig. 3G). Thus, these results suggest that multiple mechanisms, including chromatin accessibility, guarantee EPI lineage specification.

### Transcriptional regulation of trophoblast specification

**Figure 4:**
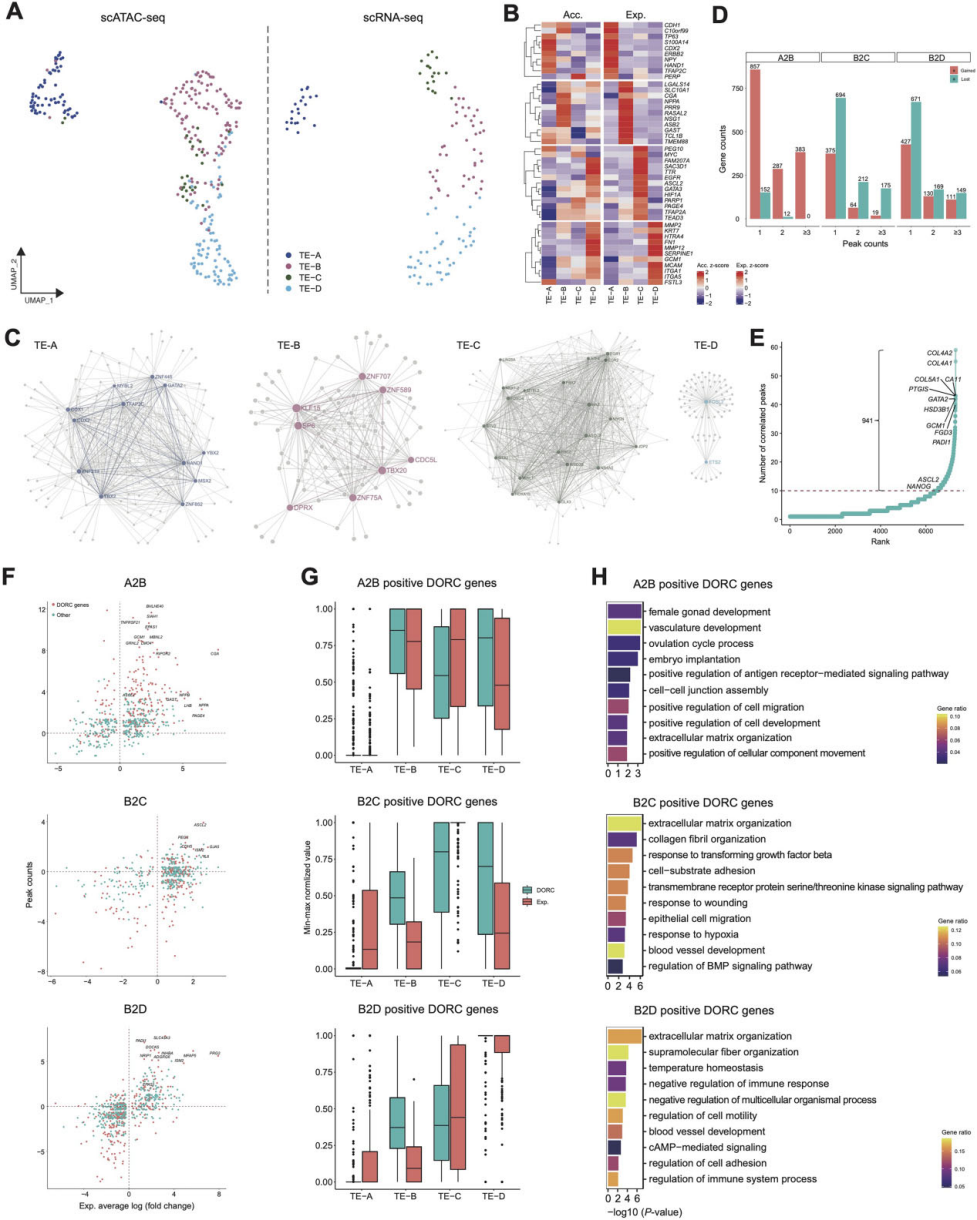
Chromatin accessibility dynamics of trophoblast specification during monkey early embryonic development. (A) UMAP plot of RNA-sequenced and ATAC-sequenced single cells derived from the trophoblast lineage, with cells colored by trophoblast subtypes. (B) Heatmaps showing averaged gene activity scores and expression levels of representative marker genes. The color bar represents the *z*-scores calculated from the TE subtype aggregates. A gradient of blue, gray, and red indicates low to high values. (C) TF regulatory networks of each trophoblast subtype are shown. Gray dots indicate target genes, colored dots indicate trophoblast subtype-specific TFs, and lines indicate regulatory relationships between TFs and target genes. (D) Bar chart showing gene numbers, with gain or loss peaks during trophoblast specification and 1, 2, and ≥3 indicating peak change numbers. (E) The number of significantly correlated peaks (*P* < 0.05) for each gene (±25 kb from transcription start sites). DORC genes situated above the dotted line are shown. (F) Changes in peak numbers and expression levels of genes during trophoblast specification from TE-A to TE-B (A2B), TE-B to TE-C (B2C), and TE-B to TE-D (B2D) are shown. The x-axis indicates the averaged log fold change between the later and earlier stages, and the y-axis indicates the corresponding number change of the accessible peak. (G) Accessible peak numbers and expression levels of upregulated DORC genes and all values were min–max normalized. (H) Bar chart showing representative GO terms enrichment in (G). *P* values derived from the hypergeometric test are shown, and the color indicates the gene ratio.

The transcriptional mechanism underlying early trophoblast specification in primates is elusive, so we then explored chromatin accessibility and gene expression profiles of trophoblasts. Louvain clustering analysis identified four distinct clusters (TE-A, TE-B, TE-C, and TE-D) in the trophoblasts. Based on scRNA-seq analysis and alignment of single scRNA-seq and scATAC-seq cells (Supplementary Fig. S4A), we annotated these four identical types of trophoblasts in scATAC-seq profiles (Fig.   4A). After considering the gene expression levels, chromatin accessibility of marker genes, and developmental trajectories (Fig. 4B and Supplementary Fig. S4B and S4C), TE-A cells were defined as TE cells, as they expressed the TE marker gene *CDX2* [24, 25]; TE-B cells were defined as early-stage CTs, as they expressed *ITGA6* [26]; and TE-C cells were defined as proliferative CTs, as they expressed the mature CT marker *KRT7* (*CK7* ) [ 26] and the CT marker *GATA3* [26] (Fig. 4B). Finally, TE-D cells were defined as EVTs, as they expressed the EVT markers *ITGA5* [26] and *FN1* [27, 28] (Fig. 4B). To define the critical TFs involved in trophoblast lineage specification, we investigated the gene expression and chromatin accessibility of lineage-specific TFs and their target genes (Supplementary Fig. S4C and Supplementary Table S6). In addition, the TF regulatory networks were generated, and key TFs involved in trophoblast lineage specification were identified: *MSX2* and *CDX2* in TE-A; zinc finger containing proteins (*ZNFs* ) including *ZNF707, ZNF75A* and *ZNF589* and et al. in TE-B; *EGR1* and *EGR2* in TE-C; and *EST2* and *FOSL2* in TE-D (Fig. 4C).

As we observed that the opening of chromatin regions preceded the gene expression alongside pseudotime ordering of single trophoblast cells during specification (Supplementary Fig. S4D), we sought to define the gene-regulatory mechanisms underlying trophoblast lineage specification by determining the gain and the loss of accessible peaks during transitions from TE-A to B, TE-B to C, and TE-B to D (Supplementary Table S7). We observed that TE-B mainly gained open chromatin peaks during differentiation from TE-A. In contrast, TE-C lost open chromatin peaks during differentiation from TE-B (Fig. 4D), and corresponding genes were ranked according to their changed peaks (Supplementary Fig. S5A). The GO term enrichment analysis of gained and lost peaks was performed, and TF motif enrichment was calculated by chromVAR [29] (Supplementary Fig. S5B and S5C). These findings imply that chromatin is primed for lineage specification in trophoblast progenitor cells and gradually closed, accompanied by the further differentiation of progenitor to mature cell types.

A previous study reported that domains of regulatory chromatin (DORCs) are enriched in lineage-determining genes and can be used to infer cell fate choices *de novo* [30]. To delineate the *cis*-regulatory programs during trophoblast specification, we defined 941 DORC genes during trophoblast specification based on previously reported criteria (regions with >10 significant peak–gene associations) [30]. Consistent with the study in mouse skin cells, DORCs were enriched for critical regulators of trophoblast lineage specification, such as *ASCL2* (regulator of human EVT differentiation) [31] and *GCM1* (essential for the differentiation of human trophoblast cells along both villous and extra-villous pathways) [32] (Fig. 4E). Next, DORCs that were activated during lineage transition were identified (Fig. 4F). As DORC activation precedes gene expressions [30], we analyzed chromatin accessibility and gene expressions of DORC genes. We also observed the chromatin activation of DORCs preceding gene expression during the differentiation from early CTs to mature CTs (TE-B to TE-C) and early CTs to EVTs (TE-B to TE-D) (Fig. 4G). These results indicate that chromatin activation is important for priming DORC loci before the final expression of DORC genes and commitment to specific cell lineages. However, during specification from TEs (TE-A) to early-stage CTs (TE-B), the activation of DORC regions and gene expressions was cogredient (Fig. 4G), indicating that another mechanism is involved in progenitor cell fate determination. Finally, the GO term enrichment analysis of DORC genes during lineage specification was performed, and the top ten GO terms are shown (Fig.   4H).

Finally, scRNA-seq–based cross-species comparisons between mouse (embryonic day 6.5–8.5) [33] and monkey (9–20 d.p.f.) gastrulation were performed as a comparable mouse scATAC-seq dataset during this stage is not available. UMAP analysis showed that monkey EPI cells overlapped with mouse EPI and primitive streak cells, monkey VE/YE cells overlapped with mouse extraembryonic and visceral endoderm cells, and monkey TE cells overlapped with mouse extraembryonic ectoderm cells. Interestingly, monkey EXMCs clustered with mouse mesenchyme, suggesting their mesenchymal cell characteristics (Supplementary Fig. S6A). Then, the DEGs were determined between monkey and mouse identical cell types, and representative genes are shown in Supplementary Fig. S6B.

### Lineage segregation between the epiblast and trophoblast

**Figure 5:**
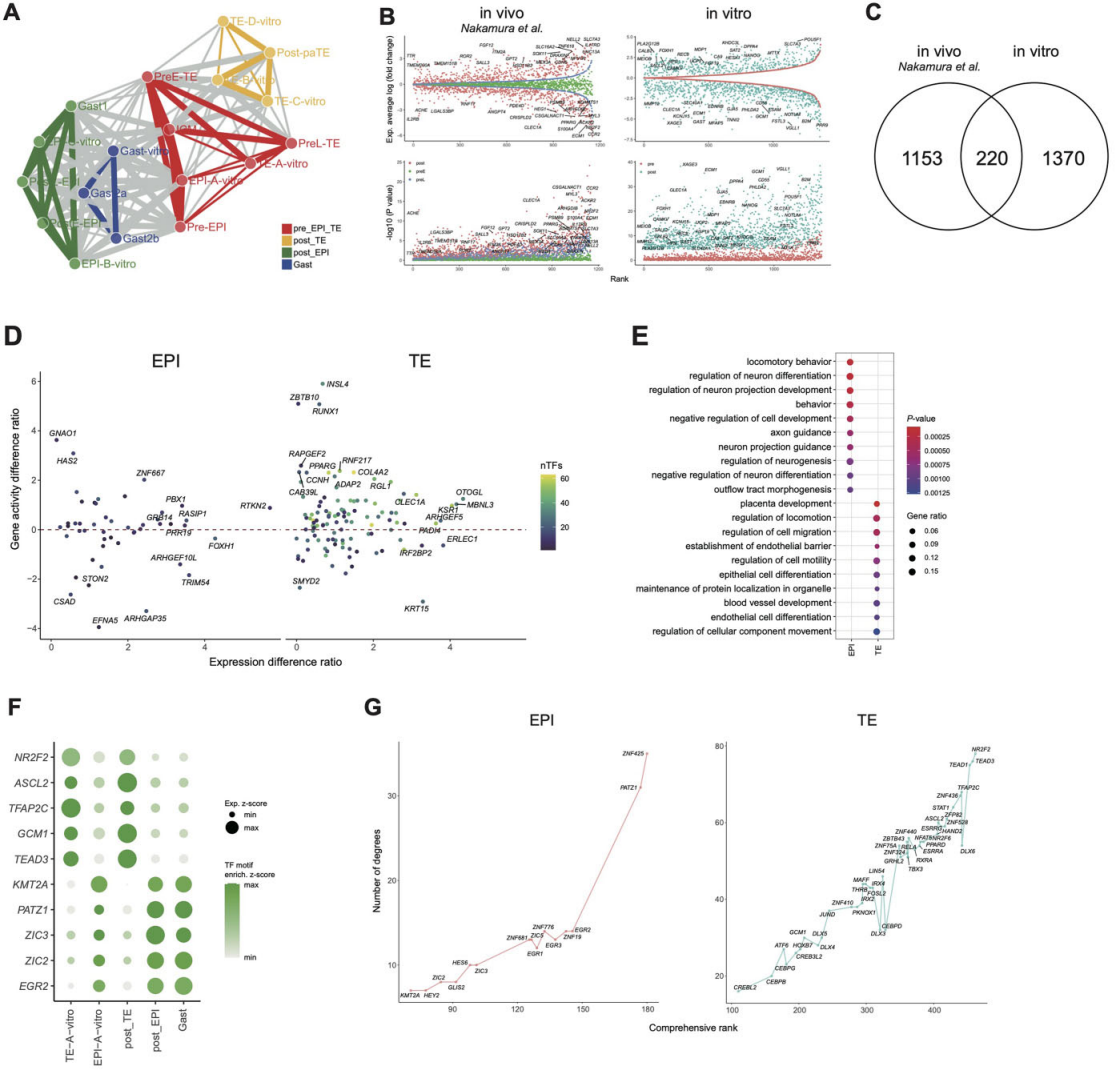
Transcriptional regulation of EPI and TE segregation. (A) Pearson correlation coefficient networks of gene expression profiles between EPI and trophoblast subtypes. Subtypes are classified into four groups based on developmental stage and hierarchical clustering. Line width represents the coefficient correlation, and the lines with a coefficient correlation <0.7 were removed. preE, pre-implantation early; preL, pre-implantation late; pre, pre-implantation; post, post-implantation; pa, parietal. (B) Averaged log fold change (top) and −log10 (*P* value) (bottom) of DEGs in the EPI and trophoblast from peri- to post-implantation of monkey embryos *in vivo* and *in vitro*. The x-axis indicates the ranked gene list, which was ordered by the averaged log fold change between EPI-A and TE-A, EPI-B and EPI-C, and TE-C and TE-D, respectively, in *in vitro* cultured embryos, and between ICM and preE-TE, pre-EPI and preL-TE, and post-implantation EPI (postE-EPI, postL-EPI, and Gast1) and post-implantation TE (post-paTE) in *in vivo* embryos [36]. (C) Venn diagram showing genes involved in EPI and trophoblast segregation. The 220 overlapping genes are conserved between embryos *in vivo* and *in vitro*. (D) Weighting the importance of the 220 genes in epiblast and trophoblast segregation. Dot chart showing gene expression level difference ratios and chromatin accessibility difference ratios in the 220 genes in (C). The ratios of the gene expression levels or chromatin accessibility differences were expressed as the post-implantation stage log fold change to the pre-implantation stage log fold change. The dot color indicates how many TFs potentially regulate a specific gene. (E) Representative enriched GO terms of 220 genes in the EPI and trophoblast. *P* values derived from the hypergeometric test are shown, and the color indicates the gene ratio. (F) Representative TFs that putatively regulate the segregation of the EPI and trophoblast. All expression levels are min–max normalized, and the motif enrichment is *z*-scores. (G) Weighting the ranking of the TFs that putatively regulate the expression of the 220 EPI and trophoblast lineage segregation genes.

Previous studies indicated lineage flexibility between naive PSCs and TEs [34, 35], but the underlying mechanism remains elusive, so we then sought to decipher the regulatory events during EPI and trophoblast lineage specification. First, the correlation of gene expression profiles between EPI and trophoblast cells in this study and the previously published dataset [5, 36] was calculated. We found that early-stage EPI cells (ICM [36] and EPI-A) highly correlated with early-stage TE cells (TE-A and pre-implantation early TE [36]) (Fig. 5A and Supplementary Fig. S7A). Moreover, the chromatin accessibility profiles also displayed a high correlation coefficient between early-stage EPI (EPI-A) and TE (TE-A) cells (Supplementary Fig. S7B). These data suggest a high similarity between early-stage EPI and TE at the transcriptional regulatory level.

To identify the key gene set involved in EPI and TE lineage specification, we explored a set of genes with expression differences gradually increasing between EPI and TE cells from the peri- to post-implantation transition in this and a previously published dataset, respectively [ 5, 36] (Fig. 5B). Next, we assessed the overlap between these two sets of genes, and 220 genes (designated as EPI-trophoblast lineage driving genes, E-T driving genes) were identified (Fig.   5C, Supplementary Table S8). To weigh the importance of the 220 genes in EPI and trophoblast lineage segregation, the absolute value of the log fold change of the averaged 220 E-T driving genes between the EPI and trophoblasts was calculated at the peri- and post-implantation stages, respectively (E-T expression difference). The same method was used to calculate the gene activity scores (E-T gene activity difference). Finally, log fold changes of the E-T expression differences and the E-T activity differences of the 220 genes were calculated. *GNAO1* in the EPI and *INSL4* in the trophoblasts were identified (Fig. 5D). GO term enrichment analysis showed that genes differentially expressed in the EPI were enriched in regulating neuron differentiation, which was also observed in early post-implantation EPI cells *in vivo* [36]. Meanwhile, genes specifically expressed in trophoblast cells were enriched in placenta development and other terms (Fig. 5E). To identify TFs that are critical for EPI and trophoblast lineage segregation, TF motif enrichment analysis for the peaks linked to the 220 genes was conducted, and the expression of TFs was also detected (Supplementary Fig. S7C). Representative TFs that potentially regulate EPI and trophoblast lineage identity are shown in Fig. 5F. To evaluate the weight of TFs in deriving the lineage specification of the EPI and trophoblasts, we devised an approach to calculate the driving potential of TFs, which putatively bound to the 220 E-T driving genes (see Methods). We observed clear ordering of *ZFNs* and *PATZ1* on the top of the list of TFs that putatively specify the EPI lineage and *NR2F2* on the top of the list of TFs that are involved in trophoblast lineage determination (Fig. 5G), a role that has been studied in humans [37]. Thus, the driving potential allows us to identify important TFs that play important roles in lineage specification.

## Discussion

After implantation, mammalian embryos undergo dramatic lineage diversification and determination, and a multifaceted regulatory process is involved to guarantee and achieve this cellular and molecular transition [38–40]. However, this multifaceted regulatory process, especially the epigenetic mechanism, remains unsolved in primates. Using the scATAC-seq approach, we delineated the chromatin accessibility landscape of *in vitro* cultured monkey embryos. Despite these advances, a low-throughput manual method was used here, and some cell lineages may have been entirely missed. In the future, high-throughput and spatial omics sequencing methods will provide more information on primate early post-implantation development.

In integrative scRNA-seq and scATAC-seq analysis, we observed chromatin opening before gene expression during EPI specification, suggesting EPI cells are permissive after implantation and ready for rapid differentiation. Lineage priming was also observed during the early differentiation of CTs to mature CTs or EVTs, but not during the specification of TE cells to early CTs. This observation is inconsistent with human hematopoietic stem cells and mouse skin cells [30, 41], implying that in addition to chromatin accessibility, another mechanism may underlie progenitor specification in monkey trophoblasts. Thus, we speculate that chromatin is primed before cell fate determination in cells requiring rapid specification.

Naive PSCs have been reported to possess trophoblast differentiation capability [34, 35, 42–45]. Is this a conserved cellular mechanism in PSCs, or does it only exist in cultured PSCs, and which TF networks and signaling pathways are involved in this process? These questions remain unanswered. In this study, we identified similarities between early-stage EPI and TE cells regarding gene expression and chromatin accessibility. Furthermore, after leveraging the “driving potential” calculation, we identified a group of TFs that may be involved in the trophoblast differentiation of naive PSCs. Among them, *NR2F2* has been confirmed as a marker of trophectoderm maturation [37]. Further studies that combine lineage tracing and genome editing will help us to understand early cell identity and plasticity.

Taken together, our findings help us understand the transcriptional regulation of primates’ early post-implantation development and provide a valuable resource for regenerative medicine.

## Methods

### Animals

Healthy cynomolgus monkeys aged 5 to 12 years were used in this study. Monkeys were usually housed in groups. During superovulation and oocyte collection, they were provisionally caged individually at 16‒26°C under 40% to 70% relative humidity and a 08:00 to 20:00 light vs. dark photoperiod, and they were provided with commercial pelleted food and water *ad libitum*. Vaginal bleeding was observed twice daily to detect the onset of menses; the beginning of bleeding was defined as the first day of menstruation.

### Single-cell collection

The monkey embryos were cultured as previously reported [5]. After washing with phosphate-buffered saline (PBS) (MA0008; Meilunbio), the embryos were cut into several pieces with a 1-mL syringe and digested into single cells with 0.1% trypsin (25200–072; GIBCO) at 37°C for 3 to 5 minutes. After neutralization with 2% fetal bovine serum (04–002-1A; Biological Industries), the cells were washed with ice-cold PBS containing 0.1% to 1% bovine serum albumin. Finally, the individual cells were picked into ice-cold lysis buffer on ice with a mouth pipette for single-cell ATAC library construction as previously described [5].

### Preprocessing of scRNA-seq data

For the preprocessing of raw sequencing data, we removed adapters and filtered out low-quality reads with an N rate >0.2 using Cutadapt (v1.15, RRID:SCR_011841) [46]. Filtered reads were then aligned to the *Macaca fascicularis* genome (Macaca_fascicularis_5.0) using STAR (v2.5.3, RRID:SCR_004463) [47]. Rsem-Calculate-Expression (RSEM; RRID:SCR_013027) (v1.3.0) [48] was used to calculate the read counts, which were then quantified as transcripts per million mapped reads (TPM). Cells with mapped reads <1 million and ≤2,000 genes with TPM values >1 were filtered out.

### Cell clustering and UMAP projection

We selected the top 2,000 variable genes based on log-transformed TPM matrices using Seurat (v3.2.2, RRID:SCR_016341) [17]. Principal components analysis (PCA) was performed, and the first 30 principal components were used to build an SNN graph using the “FindNeighbors” function in the R package Seurat. We used UMAP (RRID:SCR_018217) [49] to visualize the distance between the cells on a 2-dimensional map and Harmony (v1.0, RRID:SCR_018809) [50] to remove the batch effects between the embryos. Cell clustering was performed using the “FindClusters” function in Seurat and cell-type annotations based on known cell type–specific marker genes. We identified marker genes between the clusters using the “FindAllMarkers” function in Seurat (*P* < 0.05). The top 2,000 DEGs were selected to construct the trajectory model using Monocle2 (v2.18.0, RRID:SCR_016339) [19, 51].

### Comparisons between EPI cells *in vitro* and *in vivo*

We extracted the overlapping genes in EPI cells between our *in vitro* and *in vivo* embryos [36]. Then R package Seurat was used to integrate our *in vitro* and *in vivo* datasets. The log-transformed count expression matrices of genes × cells were used to create the Seurat object, and then we used normalized values processed by the “NormalizeData” function according to the tutorial of Seurat. The top 2,000 variable genes were identified using the “FindVariableFeatures” function, and scale gene expression values were obtained using the “ScaleData” function. The anchors between our and *in vivo* data were found with the FindTransferAnchors function (anchor.features = 2,000, reduction = “cca,” dims = 1:30). The “IntegrateData” function (dims = 1:30) was applied to our and *in vivo* datasets to get an integrated Seurat object. Then the integrated Seurat object was processed with “ScaleData,” “RunPCA,” and “RunUMAP” functions, and a UMAP graph was constructed to visualize the similarities between *in vitro* and *in vivo* embryos. Next, we averaged the gene expression levels of each cell cluster in both datasets and calculated the Pearson correlations between the cell clusters in both datasets. The Euclidean distance between the cell clusters was calculated, and unsupervised hierarchical clustering was performed to determine gene expression pattern similarities between the *in vitro* and *in vivo* cell clusters.

### Preprocessing of scATAC-seq data

The raw sequencing data were filtered using Cutadapt (v1.16) [46], and then filtered fragments were aligned to the *M. fascicularis* genome (Macaca_fascicularis_5.0) using Bowtie2 (v2.2.5, RRID:SCR_016368) [52]. Fragments with an alignment quality of >Q30 were retained, and duplicate fragments were removed. We filtered out cells whose usable fragments were <10,000 and promoter regions (500 bp around the transcriptional start site) with a ratio of fragments <10%. Sambamba (v0.6.6) [53] was used to aggregate the fragments of all cells. Reference peaks were constructed using MACS2 (v2.1.2, RRID:SCR_013291) [54]. Finally, we counted fragments in the reference peaks using the “getCounts” function in chromVAR (v1.4.0) [29].

### scATAC-seq dataset analysis

Signac (v0.2.5, RRID:SCR_021158) [55] was used to analyze the processed scATAC-seq dataset. We used latent semantic indexing and UMAP to reduce the dimensions and visualize for the scATAC-seq dataset (dims = 1:6). Gene activity scores were calculated using the “FeatureMatrix” function to count fragments in the 2-kb upstream regions of genes and gene bodies. Cell clustering was performed using Seurat’s “FindNeighbors” and “FindClusters” functions. Differentially accessible regions were identified using the “FindMarkers” function in Seurat with parameters of min.pct = 0.2 and test.use = “LR.” The per-cell motif activity score was computed by chromVAR (“RunChromVAR” function in Signac). Finally, TF motif enrichment of differentially accessible regions was performed using the “FindMotifs” function.

### Integrated analysis of scRNA-seq and scATAC-seq datasets

The gene activity value was obtained by calculating the fragments in the 2-kb upstream region and gene body, which could be used to measure the gene accessibility and for correlation analysis with the gene expressions. scATAC-seq and scRNA-seq pairs were matched by Seurat’s CCA using the “FindTransferAnchors” function (dims = 1:30, reduction = “cca”) based on the top 2,000 variable genes identified by “FindVariableFeatures” function, and then scRNA-seq cell-type annotations information was transferred to scATAC-seq using the “TransferData” function. To generate coembedding UMAP, “FindIntegrationAnchors” (anchor.features = 2,000, dims = 1:30) and “IntegrateData” (dims = 1:30) functions in Seurat were used. As previously described [56], we identified peak-to-gene links based on the null *trans* correlations. Differential peak-to-gene linkages were visualized by ComplexHeatmap (v2.7.8.1000, RRID:SCR_017270) [57]. GO analysis of the corresponding genes was performed using clusterProfiler (v3.18.1, RRID:SCR_016884) [58].

For constructing the TF–target gene network, we first identified the TFs and marker genes highly expressed in the same group of cells. Next, we identified TF–target gene pairs, that is, if the marker gene was linked with peaks that matched the corresponding TF motif. For a given marker gene with at least one linked and matched peak, we summed their squared correlation *R*² as the linkage score for the TF–target pair. NetworkD3 (v0.4) package [59] in R V4.1.0 was used to visualize the TF–target gene network. We reconstructed the TF regulatory network using the dataset from the corresponding lineage for subtype analysis in each lineage.

### Establishment of ATAC–RNA cell pairs and development trajectory analysis

ATAC and RNA cells were paired by the “pairCells” function in R package FigR [55] based on coembedding PCA components, and pairs with ATAC and RNA cells in the same cell type were retained.

Since ATAC–RNA cell pairs were established, we could get “pseudo cells” in which each “pseudo cell” had chromatin accessibility and gene expression information like multimodal data. Then, paired RNA cells were used to create a pseudotime trajectory using R package monocle2.

For the trajectory of EPI, we identified pseudotime-dependent genes whose expression varied with pseudotime using the “differentialGeneTest” function (fullModelFormulaStr = “∼sm.ns(Pseudotime)”), and genes with *Q*-values <0.1 were retained. Pseudotime-dependent genes were split into 4 main clusters based on expression and further split into some subclusters in each main cluster based on chromatin accessibility using the “plot_pseudotime_heatmap” function. ATAC and RNA pseudotime heatmaps were combined using the “Heatmap” function in the R package ComplexHeatmap. Other pseudotime analysis methods, such as Slingshot (RRID:SCR_017012) [20] and TSCAN [21], were also used to create an EPI developmental trajectory.

For the trajectory of TE, we identified branch-dependent genes whose expression varied with the branch using the “BEAM” function, and genes with *Q*-values <0.001 were retained. Branch-dependent genes were split into four main clusters based on expression and further split into some subclusters in each main cluster based on chromatin accessibility using the “plot_genes_branched_heatmap” function. ATAC and RNA branched pseudotime heatmaps were combined using the “Heatmap” function in the R package ComplexHeatmap.

### Comparison of scRNA-seq datasets among mouse and monkey embryos

To get coembedding UMAP for mouse and monkey embryos, we extracted cells in embryonic day 6.5 to 8 mouse embryos. In total, 17,358 common genes were retained in mouse and monkey embryo data based on a homologous gene list from Ensembl BioMart. Then, Seurat objects of mouse and monkey embryos were created, and the Seurat object of mouse embryos was split into three objects based on sequencing batch. Four objects were normalized using the “NormalizeData” function, and their variable genes were identified using the “FindVariableFeatures” function. The integration features between four objects, obtained using the “SelectIntegrationFeatures” function, were inputted into the “ScaleData” and “RunPCA” functions to perform PCA. Then, we identified “anchors” between four objects by the “FindIntegrationAnchors” function (reduction = “rpca,” k.anchor = 20) and integrated four objects using the “IntegrateData” function. Coembedding UMAP was generated by an integrated object using the “RunUMAP” function.

Aiming to study the differences between monkey and mouse embryos in gene expressions, we matched cell types of cross-species in the shared neighborhood based on coembedding UMAP and got mouse EPI, VE/YE, EXMC, and TE corresponding cell types in monkey embryos. Conserved cell-type markers were identified using “FindConservedMarkers” functions with max *P* values <0.05 and min log_2_ fold changes >0.25. DEGs were identified using “FindMarkers” functions in corresponding cell types between species with *P* values <0.05 and log_2_ fold changes >0.25. Then, shared genes in DEGs with cell-type markers were retained.

### Analysis of gained and lost peaks

We identified the accessible peaks (peak read count was >0) in each stage (the percentage of cells with accessible peaks was >0.25). The gained peaks at a particular stage were defined as the accessible peaks nonoverlapping with a previous stage. The lost peaks at a specific stage were defined as the peaks nonexisting with this stage compared to a previous stage. Gained and lost peak-to-gene linkages were visualized by ComplexHeatmap (RRID:SCR_017270) [56]. GO analysis of the corresponding genes was performed using clusterProfiler (RRID:SCR_016884) [58].

### Identification of genes involved in EPI and TE lineage specification

To study the genes with increasing expression differences during lineage differentiation, we identified the DEGs in EPI and TE subtypes using the “FindMarkers” function in Seurat and selected genes with increasing log fold change −log *P* values. EPI and TE upregulated genes were classified based on the log fold change values.

### Identification of TF regulating lineage specification

TF motif enrichment analysis was performed for genes upregulated in the EPI and TE. TF motif enrichment in the peaks of EPI and TE genes, *P* values less than 0.01 in EPI and greater than 0.01 in TE, and the enrichment fold changes in EPI larger than that in TE were identified as EPI-regulated TFs and vice versa.

To examine the importance of TFs in the corresponding group regulatory network, we calculated the degree centrality, closeness centrality, and eigenvector centrality of each TF in the network and the rank, respectively. The comprehensive rank was obtained by adding the ranks of the 3 centralities, and the higher the rank, the more influential the TFs in the network.

## Data Availability

All sequencing data were deposited at the National Center for Biotechnology Information Sequence Read Archive under accession no. SRP175059. The data were also deposited at the China National GeneBank (CNGB) Nucleotide Sequence Archive under accession no. CNP0000231. The mouse embryo scRNA-seq dataset was downloaded from EMBL-EBI ArrayExpress under accession no. E-MTAB-6967. All supporting data and materials are available in the *GigaScience* GigaDB database [60].

## Additional Files

**Supplementary Table S1**. Quality control data for the scATAC-seq dataset.

**Supplementary Table S2**. Lineage-specific marker peaks and corresponding genes.

**Supplementary Table S3**. Lineage-specific TFs and their candidate target genes.

**Supplementary Table S4**. Gained or lost peaks and corresponding genes during EPI lineage transition.

**Supplementary Table S5**. EPI subtype lineage-specific TFs and corresponding target genes.

**Supplementary Table S6**. Trophoblast subtype lineage-specific TFs and corresponding target genes.

**Supplementary Table S7**. Gained or lost peaks and corresponding genes during trophoblast lineage transition.

**Supplementary Table S8**. Gene list of 220 genes involved in EPI and trophoblast lineage segregation.

**Supplementary Fig. S1**. Quality control of scATAC-seq data. (A) Bar charts showing the distribution of embryonic day in each cell type. Left panel, scATAC-seq dataset; right panel, scRNA-seq dataset. (B) Violin chart showing the quality control of the scATAC-seq dataset for each embryo. (C) Coembedded UMAP for single-cell pairs of scATAC-seq and scRNA-seq datasets based on geodesic distance-based pairing approach. The colors of cells represent technology and cell type. (D) The distribution of prediction scores calculated in the integrated procedure. (E) Percentage of each cell type in scATAC-seq and scRNA-seq datasets. (F) The chromatin accessibility and gene expression levels of cell-type markers in the individual cell. Each gray line indicates one ATAC–RNA single-cell pair. (G) UMAP plot of gene expression levels and motif deviation scores of lineage-specific TFs.

**Supplementary Fig. S2**. Lineage specification of EPI. (A) Pseudotime trajectories of EPI cells inferred by slingshot (left) and TSCAN (right) analysis. (B) Hierarchical cluster analysis of expression profiles of EPI subtypes between *in vivo* [36] and *in vitro* embryos. (C) Bar chart showing the number of genes that gained or lost peaks during EPI subtype transitions. Genes are grouped by the number of peaks changed. (D) The number of accessible peaks changed for each gene during EPI subtype transitions. (E) Heatmap showing gained peaks and corresponding genes during EPI subtype transitions with listed well-studied marker genes, TF binding motifs, candidate TFs, and enriched GO terms. *P* values derived from the hypergeometric test are shown, and the color indicates the gene ratios. (F) Heatmap showing lost peaks and corresponding genes during EPI subtype transitions with listed well-studied marker genes, TF binding motifs, candidate TFs, and enriched GO terms. *P* values derived from the hypergeometric test are shown, and the color indicates the gene ratio.

**Supplementary Fig. S3**. The correlation between gene expression and chromatin accessibility during EPI specification. (A) Heatmaps showing gene activity scores and expression levels of pseudotime-dependent genes in Fig. 3B. Columns of the heatmap indicate pseudotime (left panel). Representative genes and GO enrichment terms of genes are listed in the middle and right panels. *P* values derived from the hypergeometric test are shown, and the color indicates the gene ratio. (B) Pseudotime heatmaps showing gene activity scores and expression levels of EPI-C- and Gast-specific TF target genes in Fig. 3E.

**Supplementary Fig. S4**. Dynamics of trophoblast cell fate transitions. (A) Coembedded UMAP for single-cell pairs of scATAC-seq and scRNA-seq datasets based on geodesic distance-based pairing approach. The colors of cells represent technology and cell type. (B) Pseudotime trajectory of the scATAC–scRNA paired EPI subtypes with cells colored by trophoblast subtypes and pseudotime. (C) Heatmaps showing *z*-scores of trophoblast-averaged subtype-specific TFs, their target gene expression levels, and gene activity scores. Averaged gene expression levels and activity scores are calculated from cells aggregated by trophoblast subtypes. (D) Heatmaps showing gene activity scores and expression levels of branch-dependent genes in (B). Columns of the heatmap indicate pseudotime (left panel). Representative genes and GO enrichment terms are listed in the middle and right panels. *P* values derived from the hypergeometric test are shown, and the color indicates the gene ratio.

**Supplementary Fig. S5**. Dynamics of chromatin accessibility during trophoblast specification. (A) The number of changed accessible peaks of each gene during the transition of trophoblast subtypes. The genes are ranked by changed accessible peaks. (B) Heatmap showing gained peaks and corresponding genes during each trophoblast subtype transitions with listed well-studied marker genes, TF binding motifs, corresponding candidate TFs, and representative enriched GO terms. *P* values derived from the hypergeometric test are shown, and the color indicates the gene ratio. (C) Heatmap showing lost peaks and corresponding genes during each trophoblast subtype’s transitions with listed well-studied marker genes, TF binding motifs, corresponding candidate TFs, and representative enriched GO terms. *P* values derived from the hypergeometric test are shown, and the color indicates the gene ratio.

**Supplementary Fig. S6**. Cross-species comparison between mouse and monkey gastrulating embryos. (A) UMAP visualization of cells of *in vivo* mouse (embryonic days 6.5–8) and *in vitro* monkey (9–20 d.p.f.) embryos. Colors encode cell source and type. (B) Heatmap of the top 40 DEG expressions between monkey and mouse embryos. Each column indicates matched cell cluster between monkey and mouse embryos.

**Supplementary Fig. S7**. Transcriptional regulation of EPI and trophoblast lineage segregation. (A) Heatmap showing Pearson correlation coefficient of gene expression profiles between EPI and TE subtypes. The EPI and TE cells were classified into four groups based on development stage and hierarchical clustering (pre_EPI_TE, post_TE, post_EPI, and Gast). pre, pre-implantation; post, post-implantation; Gast, gastrulating cells. (B) Heatmap showing Pearson correlation coefficient of chromatin accessibility profiles between EPI and TE subtypes. The EPI and TE cells were classified into four groups (pre_EPI_TE, post_TE, post_EPI, and Gast). (C) Bubble plot showing TF expression and corresponding motif enrichments that regulate lineage differentiation. *P* values are derived from the hypergeometric test.

giad038_GIGA-D-22-00278_Original_Submission

giad038_GIGA-D-22-00278_Revision_1

giad038_Response_to_Reviewer_Comments_Original_Submission

giad038_Reviewer_1_Report_Original_Submission Zhicheng Ji -- 11/12/2022 Reviewed

giad038_Reviewer_1_Report_Revision_1 Zhicheng Ji -- 3/30/2023 Reviewed

giad038_Reviewer_2_Report_Original_Submission Ivan Costa -- 11/29/2022 Reviewed

giad038_Supplemental_Figures_and_Tables

## Abbreviations

bp: base pair; CCA: canonical correlation analysis; CT: cytotrophoblast; DEG: differentially expressed gene; DORC: domain of regulatory chromatin; DP: differential peak; dpf: day postfertilization; EPI: epiblast; E-T: EPI-trophoblast; EVT: extravillous cytotrophoblast; EXMC: extra-embryonic mesenchyme cell; GO: Gene Ontology; MNN: mutual nearest-neighbor; PBS: phosphate-buffered saline; PCA: principal components analysis; PSC: pluripotent stem cell; scATAC-seq: transposase accessible chromatin sequencing; scRNA-seq: single-cell RNA sequencing; TE: trophectoderm; TF: transcription factor; TPM: transcripts per million mapped reads; UMAP: uniform manifold approximation and projection; VE/YE: visceral endoderm or yolk-sac endoderm; ZNF: zinc finger containing protein.

## Ethics approval

The ethical committee of the State Key Laboratory of Primate Biomedical Research (LPBR) approved all animal and experiment procedures (LPBR-2016–01), and the procedures were performed by following the guidelines of the Association for Assessment and Accreditation of Laboratory Animal Care International (AAALAC) for the ethical treatment of nonhuman primates.

## Competing interests

The authors declare no competing interests.

## Funding

This work was supported by the National Natural Science Foundation of China (82192871), the Natural Science Foundation of Yunnan Province (202001BC070001 and 202102AA100053), and the China National GeneBank (CNGB).

## Authors’ contributions

H.S., N.S., C.L., L.W., Y.Y., X.W., and L.L. performed most of the experiments. X.D., B.C., and B.B. performed the bioinformatics analysis. X.D., J.W, H.Y., W.J., Z.S., and T.T. participated in discussions. X.D., J.W., Z.S., and T.T. analyzed the data and wrote the manuscript. T.T. and Z.S. conceived and supervised the study.
