# Supplementary material for: Developmental dynamics of chromatin accessibility during post-implantation development of monkey embryos
Source: Gigascience. 2023 May 25;12:giad038. doi: 10.1093/gigascience/giad038 (PMC10209733; doi:10.1093/gigascience/giad038)
Supplement: giad038_GIGA-D-22-00278_Original_Submission [file giad038_giga-d-22-00278_original_submission.pdf]

GigaScience

Developmental dynamics of chromatin accessibility during post-implantation development of monkey embryos

--Manuscript Draft--

**Manuscript Number:**	GIGA-D-22-00278
**Full Title:**	Developmental dynamics of chromatin accessibility during post-implantation development of monkey embryos
**Article Type:**	Research
**Funding Information:**	National Natural Science Foundation of China (82192871) — Dr. Tao Tan<br>Natural Science Foundation of Yunnan Province (202001BC070001 and 202102AA100053) — Dr. Tao Tan<br>the China National GeneBank — Dr. Zhouchun Shang
**Abstract:**	Abstract<br>Background: Early post-implantation development, especially gastrulation in primates, is accompanied by extensive drastic chromatin reorganization, which remains largely elusive.<br>Results: To delineate the global chromatin landscape and understand the molecular dynamics during this period, single-cell assay for transposase accessible chromatin sequencing (scATAC-seq) was applied to in vitro cultured monkey embryos to investigate the chromatin status. We delineated the cis-regulatory interactions and identified the regulatory networks and key transcription factors involved in epiblast (EPI), hypoblast, and trophectoderm (TE)/trophoblast lineage specification. We also identified the opposing roles of FGF and BMP signaling in pluripotency regulation in EPI cells. The integrative analysis of chromatin accessibility and gene expression revealed that chromatin status activation precedes gene expression during EPI and trophoblast specification. Finally, we revealed the similarity between EPI and TE in terms of gene expression patterns and demonstrated that NR2F2 and PATZ1 are involved in EPI and trophoblast specification during monkey post-implantation development.<br>Conclusions: Our findings provide new resources and insights into dissecting the transcriptional regulatory machinery during primate post-implantation development.
**Corresponding Author:**	Tao Tan<br>State Key Laboratory of Primate Biomedical Research<br>Kunming, CHINA
**Corresponding Author Secondary Information:**	
**Corresponding Author's Institution:**	State Key Laboratory of Primate Biomedical Research
**Corresponding Author's Secondary Institution:**	
**First Author:**	Tao Tan
**First Author Secondary Information:**	
**Order of Authors:**	Tao Tan
	Xi Dai
	Honglian Shao
	Nianqin Sun
	Baiquan Ci
	Jun Wu

Powered by Editorial Manager® and ProduXion Manager® from Aries Systems Corporation

	Chuanyu Liu
	Liang Wu
	Yue Yuan
	Xiaoyu Wei
	Huanming Yang
	Longqi Liu
	Weizhi Ji
	Bing Bai
	Zhouchun Shang
**Order of Authors Secondary Information:**	
**Additional Information:**	
**Question**	**Response**
Are you submitting this manuscript to a special series or article collection?	No
**Experimental design and statistics**<br><br>Full details of the experimental design and statistical methods used should be given in the Methods section, as detailed in our Minimum Standards Reporting Checklist. Information essential to interpreting the data presented should be made available in the figure legends.<br><br>Have you included all the information requested in your manuscript?	Yes
**Resources**<br><br>A description of all resources used, including antibodies, cell lines, animals and software tools, with enough information to allow them to be uniquely identified, should be included in the Methods section. Authors are strongly encouraged to cite Research Resource Identifiers (RRIDs) for antibodies, model organisms and tools, where possible.<br><br>Have you included the information requested as detailed in our Minimum Standards Reporting Checklist?	Yes

Powered by Editorial Manager® and ProduXion Manager® from Aries Systems Corporation

**Availability of data and materials**<br><br>All datasets and code on which the conclusions of the paper rely must be either included in your submission or deposited in publicly available repositories (where available and ethically appropriate), referencing such data using a unique identifier in the references and in the “Availability of Data and Materials” section of your manuscript.<br><br>Have you have met the above requirement as detailed in our Minimum Standards Reporting Checklist?	Yes

Manuscript Click here to access/download;Manuscript;manuscript.docx

Developmental dynamics of chromatin accessibility during post-implantation development of monkey embryos

Xi Dai[1, 2, #], Honglian Shao[3, 4, #], Nianqin Sun[3, 4, #], Baiquan Ci[3, 4], Jun Wu[5], Chuanyu Liu[2], Liang Wu[1, 2], Yue Yuan[2], Xiaoyu Wei[2], Huanming Yang[2, 6], Longqi Liu[1, 2], Weizhi Ji[3, 4], Bing Bai[3, 4], Zhouchun Shang[1, 2, 6*], Tao Tan[3, 4, *]

[1] College of Life Sciences, University of Chinese Academy of Sciences, Beijing 100049, China

[2] BGI-Shenzhen, Shenzhen 518083, China

[3] State Key Laboratory of Primate Biomedical Research, Institute of Primate Translational Medicine, Kunming University of Science and Technology, Kunming, Yunnan 650500, China

[4] Yunnan Key Laboratory of Primate Biomedical Research, Kunming, Yunnan, 650500, China

[5] Department of Molecular Biology, University of Texas Southwestern Medical Center, Dallas, TX 75390, USA

[6]James D. Watson Institute of Genome Sciences, Hangzhou, 310013, China

[#]These authors contributed equally to this work.

[*]Corresponding author. Email: tant@lpbr.cn.com (T.T.); shangzhouchun@genomics.cn (Z.S.).

Abstract

Background: Early post-implantation development, especially gastrulation in primates, is accompanied by extensive drastic chromatin reorganization, which remains largely elusive.

Results: To delineate the global chromatin landscape and understand the molecular dynamics during this period, single-cell assay for transposase accessible chromatin sequencing (scATAC-seq) was applied to *in vitro* cultured monkey embryos to investigate the chromatin status. We delineated the cis-regulatory interactions and identified the regulatory networks and key transcription factors involved in epiblast

(EPI), hypoblast, and trophectoderm (TE)/trophoblast lineage specification. We also identified the opposing roles of FGF and BMP signaling in pluripotency regulation in EPI cells. The integrative analysis of chromatin accessibility and gene expression revealed that chromatin status activation precedes gene expression during EPI and trophoblast specification. Finally, we revealed the similarity between EPI and TE in terms of gene expression patterns and demonstrated that NR2F2 and PATZ1 are involved in EPI and trophoblast specification during monkey post-implantation development.

Conclusions: Our findings provide new resources and insights into dissecting the transcriptional regulatory machinery during primate post-implantation development.

Keywords: cynomolgus monkey, ex vivo, gastrulation, scATAC-seq, chromatin dynamics

Background

The transition from pre-implantation to gastrulation represents a milestone of early

embryogenesis in primates and involves extensive morphogenesis and lineage specification and differentiation. During this stage, a connection between the embryo and the mother is established, while the trophectoderm (TE) differentiates into cytotrophoblasts (CTs), extravillous cytotrophoblasts (EVTs) and syncytiotrophoblasts (STs); the cavitation of the amnion and yolk sac initiates and the gastrulation of the embryo launches to form three germ layers and program the body plan of the fetus [1, 2]. However, there are technical limitations and ethical concerns, and the molecular mechanisms underlying this transition remain largely elusive. Recently, advancements in the study of *in vitro*-cultured embryos have enabled our and other groups to investigate transcriptional and DNA methylation dynamics during early embryonic development of humans and monkeys [3-7]. However, several key questions, including the chromatin status that underlies this transition, have yet to be addressed.

In the mouse, chromatin accessibility, histone modifications and 3D chromatin structures during post-implantation development have been extensively studied and epigenetic regulatory networks have been revealed [8-13]. As the large differences

exist between primates and mice in terms of post-implantation development, for example, in the morphogenesis of embryonic and extra-embryonic structures, and signaling pathways involved in the specification of embryonic and extra-embryonic lineages [1, 2, 14, 15], the knowledge derived from mouse models could not be straightforwardly extrapolated to primate models. This poses a significant limitation to studies of, for example, the regulation of pluripotent stem cells (PSCs) in primates.

Here, we harness the power of single-cell assay for transposase accessible chromatin sequencing (scATAC-seq) and *in vitro* culture system to unravel the regulatory chromatin landscape during early embryonic development in cynomolgus monkeys (*Macaca fascicularis*), as early post-implantation development is similar between the monkey and the human and global ethical considerations exist in humans (14-day rules). This study provides new resources to study chromatin dynamics and chromatin regulation during early embryonic developments in primates.

Results

Single-cell chromatin accessibility profiles of early embryogenesis in the

cynomolgus monkeys

To determine the regulatory landscape at a single-cell resolution during cynomolgus monkey peri- and post-implantation development, we performed scATAC-seq of *in vitro*-cultured cynomolgus monkey embryos from post-fertilization day 9 (d.p.f. 9) to day 20 (d.p.f. 20) as previously reported by our group and single cell RNA-seq data were used for further analysis [5] (Fig. 1A and Supplementary Fig. S1A). In total, 1,198 individual cells were sequenced, and after applying stringent filtration (usable fragments > 10,000, promoter fragments ratio > 10%), 978 high-quality single nuclei, which distributed from d.p.f. 9 and d.p.f. 20, were retained (Supplementary Table S1 and Supplementary Fig. S1A). Cells within per embryo passing filter had the median fragments ranged from 20,020 to 61,182, the median fraction of fragments in promoters (500 bp around transcriptional start site) ranged from 12.14% to 19.01% and the median fraction of fragments in peaks ranged from 51.37% to 64.39 % (Supplementary Fig. S1B).

Based on these high-quality data, we investigated global gene regulatory activities during cynomolgus monkey early development. First, the resting 978 cells were

dimensionally reduced using UMAP and clustering analysis. In scRNA-seq analysis, four main cell clusters, namely epiblast (EPI), trophectoderm/trophoblast (TE), yolk-sac or visceral endoderm (VE/YE), and extra-embryonic mesenchyme cell (EXMC) were identified (Fig. 1B). In addition, coembedding analysis of scRNA-seq and scATAC-seq data revealed a consistent overlap of all cell types (Supplementary Fig. S1C), suggesting the power of scATAC-seq in cell identity identification. Next, increased and unique ATAC-seq peaks in promoter and distal regions of cluster-specific marker genes were identified, including OCT4 locus (also known as POU5F1) in the EPI, TFAP2C in the TE, HNF1B in the VE/YE, and TCF 21 in the EXMCs (Fig. 1C). Furthermore, the binding motifs of these marker genes were also enriched in the four major cell clusters, which was accompanied by cluster-specific expressions of the marker genes (Supplementary Fig. S1D).

We related the cluster-specific differentially peaks (DPs) to the differentially expressed genes (DEGs) (Supplementary Table S2 and Fig. 1D). Furthermore, the enrichment of well-known lineage specific TFs binding motifs was observed for cluster specific DPs, such as OCT4 (POU5F1) and NANOG in the EPI, TFAP2C and

TEAD4 in the TE, GATA4 in the VE/YE, and TCF21 and FOXF1 in EXMCs (Fig. 1E). After confirming the above observations, the most enriched Gene Ontology (GO) terms for DPs related to DEGs in the EPI included early-development associated terms such as anterior/posterior pattern specification and embryo development, and the most enriched GO terms in the VE/YE included epithelium development and regulation of WNT signaling. According to the cell identity of EXMCs, mesenchyme development associated terms were enriched in this cell lineage. Interestingly, we observed inflammatory response, regulation of immune system processes and other GO terms enriched in the TE cells, suggesting their role in immune regulation during pregnancy [16] (Fig. 1F). Taken together, these findings indicate that combining embryo *in vitro* culture platform with powerful single cell chromatin assays can successfully generate comprehensive and high-quality maps of open chromatin corresponding to major cell lineages and lineage regulators during monkey early embryogenesis.

Lineage specific transcriptional regulatory network of early embryonic

development in monkey

To further characterize the transcriptional regulatory networks of early embryonic development in the monkey, we identified lineage specific TFs and their enriched motifs as well as lineage specific DPs. In addition, the gene activity scores and expression levels of the TF target genes were analyzed. Based on the above analysis, we constructed a TFs data resource (Supplementary Table S3), which may play important roles in main cell lineage specification during monkey early embryogenesis. The top ten lineage specific TFs are shown in Fig. 2A, and among them several well-studied cell lineage markers were identified, including POU5F1 (OCT4), HNF4A, CREB3L1 and GCM1 (Fig. 2A and b). Next, leveraging identified lineage specific TFs and their target genes, we constructed modules of lineage specific TFs and regulatory networks of target genes that were putatively co-regulated by two lineage specific TF modules (EPI-VE/YE, EXMC-VE/YE, EXMC-TE and TE-VE/YE). EPI and VE/YE lineages were highly related in this network by hub TFs, such as FOXH1, indicating their similarities in the regulatory program of early embryogenesis, while TE and EXMC lineages were distinct from the other lineages

(Fig. 2C). The top five GO enrichment terms of target genes are shown in Fig. 2D. Consistent with the transcriptional regulatory network analysis, we observed that the chromatin regions of co-regulated genes were more accessible in both lineage specific TF modules and the expression of genes displayed a lineage specific pattern, suggesting that besides chromatin accessibility, additional mechanisms exist to guarantee lineage specification during early embryogenesis such as FOXH1 target genes in the EPI and VE/YE (Fig. 2E), as these genes are involved in WNT signaling pathway in the EPI and in VE/YE (Fig. 2F).

Single cell chromatin accessibility reveals regulatory mechanisms of EPI lineage

To explore the regulatory mechanisms underpinnings the identity of EPI cells, we integrated the scRNA-seq and scATAC-seq datasets to query the relationship and the congruence between chromatin accessibility and gene expression using Seurat [17]. The coembedded UMAP plot identified four cell clusters, designate as EPI-A, EPI-B, EPI-C and gastrulating cell (Gast)) (Fig. 3A). We also observed that most of cells in the scRNA-seq and scATAC-seq datasets overlapped with each other, suggesting that

the chromatin accessibility and gene expression in most EPI cells occur in a concordant manner during early embryonic development (Fig. 3A). Next, the developmental trajectories of EPI-A, -B, -C and Gast cells were constructed. We observed that all cells were ordered in a U-like trajectory, with EPI-A cells occupying one end and Gast cells occupying the other end, while EPI-B and -C cells were in the middle (Fig. 3B). This trajectory revealed the continuous differentiation of EPI-A to Gast cells, as EPI-A cells are equivalent to ICM cells, EPI-B cells are equivalent to EPI cells at the pre-implantation stage (EPI-A and B were subsequently renamed as early EPI, early), EPI-C cells are equivalent to EPI cells at the early post-implantation stage and Gast cells are equivalent to *in vivo* gastrulation cells (Supplementary Fig. S2A).

To delineate the regulatory role of chromatin accessibility in EPI cell lineage progression, we examined the cell identity of specific DPs from EPI-A to Gast cells. During the transition from EPI-A to B and EPI-C to Gast, most gene regions tended to gain accessibility, whereas they lost accessibility during the transition from EPI-B to C (Supplementary Fig. S2B and Supplementary Table S4). Notably, Hox gene

activation was observed when the EPI cells underwent gastrulation, indicating their important roles in primitive streak formation (Supplementary Fig. S2C). Interestingly, we observed that the activation of chromatin states preceded the expression of genes (Supplementary Fig. S2D), which implies that there is a time lag between gene expression and chromatin accessibility during EPI cell specification. GO terms and TF binding motif enrichment analyses of DPs indicated that OTC4 and SOX2 may be involve in the transition from EPI-A to B and early post-implantation stage EPI (EPI-C) to gastrulating cells (Gast) (Supplementary Fig. S2D and 2E).

To further study the relationship between chromatin accessibility and gene expression, we related the DPs to the DEGs and two patterns of genes expression and chromatin accessibility were observed. In the first pattern, the opening of chromatin regions preceded the gene expression (designated as the preceding manner), and in the second pattern, the opening of chromatin regions occurred concurrently with the gene expression (designated as the synchronized manner) (Fig. 3C and D). In the preceding manner, four sub-patterns (clusters 1-4) of gene expression and chromatin accessibility were identified and GO analysis was performed. For example,

pluripotency related TFs, such as POU5F1, SOX2, and NANOG, belong to cluster 2, in which chromatin opens at pre- and peri-implantation stages (EPI-A and EPI-B, early-stage EPI), and gene expression was up-regulated until the post-implantation stage (EPI-C) (Fig. 3C), which was also observed during the establishment of mouse PSCs in different pluripotent states [18]. In the synchronized manner, five clusters of chromatin accessibility and gene expression patterns were observed. Interestingly, genes involved in mesoderm formation and gastrulation belonged to the synchronized manner (Fig. 3D).

As the little is known about cell identity and pluripotency regulation of post-implantation EPI cells, we next focused our analysis on EPI-C and Gast cells. We identified EPI-C and Gast specific TFs (Supplementary Table S5), and the chromatin accessibility and gene expression of TF target genes were determined. We observed that chromatin became accessible preceding the gene expression, indicative of an initial priming process of post-implantation EPI cells prior to their commitment to the specific lineage (Fig. 3E). We further investigated mechanisms leading to the pluripotency transition from early-stage EPI to Gast. We observed that the

expressions of core pluripotent factors, such as OCT4 and NANOG, was upregulated in EPI-C cells, which highly correlated with the expression of FGF signaling members such as FGF2, FGF4 and FGF receptor 1 (FGFR1) (Fig. 3F). Furthermore, the expressions of gastrulation marker genes, including TBX3 and CDH2, was highly correlated with BMP and WNT signaling. Neither FGF nor BMP signaling could regulate the expressions of naïve pluripotency related genes [19] (Fig. 3F), and the activation of chromatin regions corresponding to FGF and BMP signaling members preceded their expression (Fig. 3G). Thus, these results suggest that multiple mechanisms, including chromatin accessibility, guarantee EPI cell lineage specification.

Transcriptional regulation of trophoblast specification during monkey early embryogenesis

Presently, little is known about the transcriptional mechanism underlying primate early trophoblast specification in primates. Thus, we explored chromatin accessibility and gene expression profiles of trophoblast in *in vitro* cultured monkey embryos.

Louvain clustering analysis identified four distinct clusters (TE-A, TE-B, TE-C, and TE-D) in the trophoblast. Based on scRNA-seq analysis, we annotated these four identical types of trophoblasts (TE-A, TE-B, TE-C, and TE-D) using scATAC-seq profiles (Fig. 4A). After considering the gene expression levels, chromatin accessibility of marker genes and developmental trajectories (Fig. 4B and Supplementary Fig. S3A), TE-A cells were defined as trophectoderm cells (TEs), as they expressed the TE marker gene CDX2 [20, 21]; TE-B cells were defined as early stage cytotrophoblasts (CTs), as they expressed ITGA6 [22], and TE-C were defined as the proliferative CTs, as they expressed the mature CT marker KRT7 (CK7) [22] and the CT markers GATA3 [22] (Fig. 4B). Finally, TE-D were defined as EVTs, as they expressed the EVT markers, ITGA5 [22] and FN1 [23, 24] (Fig. 4B). To define the key TFs involved in trophoblast lineage specification, we investigated the gene expression and chromatin accessibility of lineage specific TFs and their target genes (Supplementary Fig. S3B and Supplementary Table S6). In addition, the TF regulatory network was generated, and key TFs involved in trophoblast lineage specification were identified. MSX2 and CDX2 in TE-A, zinc finger containing

proteins (ZNFs) including ZNF707, 75A and et al in TE-B, EGR1 and EGR2 in TE-C, and EST2 and FOSL2 in TE-D were identified (Fig. 4C).

To further define the gene-regulatory mechanisms underlying trophoblast lineage specification with the developmental paths that we have constructed, we determined the gain and the loss of accessible peaks during specification from TE-A to B, TE-B to C and TE-B to D (Supplementary Table S7). We observed that TE-B mainly gained open chromatin peaks during differentiation from TE-A, whereas TE-C lost open chromatin peaks during differentiation from TE-B (Fig. 4D) and corresponding genes were ranked according to their changed peaks (Supplementary Fig. S3C). GO term enrichment analysis of gained and lost peaks was performed, and TF motif enrichment was calculated by chromVAR [25] (Supplementary Fig. S3D and E). Taken together, these findings imply that chromatin is primed for lineage specification in trophoblast progenitor cells and gradually “closed”, which is accompanied by the further differentiation of progenitor to mature cell types.

Previous study reported that domains of regulatory chromatin (DORCs) are enriched for lineage-determining genes and can be used to infer cell fate choices *de*

novo [26]. To delineate the cis-regulatory programs during trophoblast specification, we defined 941 DORC genes during trophoblast specification based on previously reported criteria (regions with >10 of significant peak-gene associations) [26]. Consistent with study in mouse skin cells, DORCs were enriched for key regulators of trophoblast lineage specification, such as ASCL2 (regulator of human EVT differentiation) [27] and GCM1 (important for the differentiation of human trophoblast cells along both villous and extra-villous pathways) [28] (Fig. 4E). Next, DORCs that were activated during lineage transition were identified (Fig. 4F). As DORCs activation precedes gene expressions [26], we analyzed chromatin accessibility and DORC gene expression. We also observed the chromatin activation of DORCs preceding gene expression during the lineage specification of early CTs to mature CTs (TE-B to TE-C) and early CTs to EVTs (TE-B to TE-D) (Fig. 4G), and the results indicate that chromatin activation is important for the priming of DORC loci before final expression of DORC genes and commitment to specific cell lineages. However, during specification from TEs (TE-A) to early stage CTs (TE-B), the activation of DORC regions and gene expressions were cogredient (Fig. 4G),

indicating that another mechanism is involved in progenitor cell fate determination. Finally, GO term enrichment analysis of DORC genes during lineage specification was performed and the top ten GO terms are shown (Fig. 4H).

Lineage segregation between the epiblast and trophoblast

Previous studies indicated that lineage flexibility exists between naïve PSCs and TEs [29, 30]; however, the underlying regulatory mechanism remains elusive. We deciphered the regulatory events during EPI and trophoblast lineage specification. The correlation of gene expression patterns between EPI and trophoblast cells in this study and previously published dataset [5, 31] were calculated. We found that early stage EPI cells (ICM [31] and EPI-A) highly correlated with early stage TE cells (TE-A and pre-implantation early TE) [31] (Fig. 5A and Supplementary Fig. S4A). Moreover, the chromatin accessibility profile also displayed a highly correlation coefficient between early-stage EPI (EPI-A) and TE (TE-A) cells (Supplementary Fig. S4B). These data suggest that there is a high degree of similarity between early-stage EPI and TE at the transcriptional regulatory level.

To identify the key gene set involved in EPI and TE lineage specification, we explored a set of genes with expression differences gradually increasing between EPI and TE cells from peri to post-implantation transition in this and previously published dataset, respectively [5, 31] (Fig. 5B). Next, we assessed the overlap between these two sets of genes and 220 genes (designated as EPI-trophoblast lineage driving genes, E-T driving genes) were identified (Fig. 5C, Supplementary Table S8). To weight the importance of the 220 genes in EPI and trophoblast lineage segregation, the absolute value of the log fold change of the averaged 220 E-T driving genes between the EPI and trophoblast were calculated at peri and post-implantation, respectively (E-T expression difference). The same method was used to calculate the gene activity scores (E-T gene activity difference). Finally, a log fold changes of the E-T expression differences and the E-T activity differences of the 220 genes were calculated. GNAO1 in the EPI and INSL4 in the trophoblast were identified (Fig. 5D). GO term enrichment analysis showed that genes differentially expressed in the EPI were enriched in the regulation of neuron differentiation, which was also observed in early post-implantation EPI cells *in vivo* [31]. Meanwhile, genes specifically expressed in

trophoblast cells were enriched in placenta development and other terms (Fig. 5E). To identify TFs that are critical for EPI and trophoblast lineage segregation, TF motif enrichment analysis for the peaks linked to the 220 genes was conducted and the expression of TFs was also detected (Supplementary Fig. S4C). Representative TFs that potentially regulate EPI and trophoblast lineage identity are shown in Fig. 5F. To evaluate the weight of TFs in deriving the lineage specification of the EPI and trophoblast, we devised an approach to calculate the driving potential of TFs, which putatively bound to the 220 E-T driving genes (see Methods). We observed clear ordering of ZFNs and PATZ1 on the top of the list of TFs that putatively specify the EPI lineage and NR2F2 on the top of the list of TFs that are involved in trophoblast lineage determination (Fig. 5G), a role of that has been studied in humans [32]. Thus, the driving potential allows us to identify important TFs that play important roles in lineage specification.

Discussion

After implantation, mammalian embryos undergo dramatic lineage diversification and determination, and a multi-faceted regulatory process is involved to guarantee and achieve this cellular and molecular transition [33-35]. However, this multi-faceted regulatory process, especially the epigenetic mechanism, remains unsolved in primate post-implantation development. Using the scATAC-seq approach, we delineated the chromatin accessibility landscape of *in vitro* cultured cynomolgus monkey embryos. Although the development of *in vitro* cultured embryos was slower than that of *in vivo* embryos, this study provides new insights on the transcriptional regulation of primate post-implantation development. Here, a low-throughput manual method was used, and some cell lineages may have been entirely missed. In the future, high-throughput methods and spatial omics sequencing methods will provide more information on primate early post-implantation development.

In integrative scRNA-seq and scATAC-seq analysis, we observed EPI cells in the post-implantation stage, which included early and gastrulating cells that were primed at the chromatin level just prior to final lineage commitment. This finding indicates that EPI cells are in a permissive state after implantation and ready for rapid

differentiation into distinct cell types, which is important for gastrulation stage. Lineage priming was also observed during early differentiation of CTs to mature CTs or EVTs, but not observed during specification of TE cells to early CTs. This observation is inconsistent with that of human hematopoietic stem cells and mouse skin cells [26, 36], implying that in addition to chromatin accessibility, another mechanism may underlie progenitor specification in monkey trophoblasts. Based on the above findings, we speculate that chromatin is primed prior to cell fate determination in cells requiring rapid specification.

Naïve PSCs have been reported to possess trophoblast differentiation capability [29, 30, 37-40]. Is this a conserved cellular mechanism in PSCs or does it only exists in cultured PSCs, and which TFs networks and signalling pathways are involved in this process? These questions remain unanswered. In this study, we identified similarities between early stage EPI and TE cells in terms of gene expression and chromatin accessibility. Furthermore, after leveraging the ‘driving potential’ calculation, we identified a group of TFs that may regulate trophoblast differentiation of naïve PSCs. Among them, NR2F2 has been confirmed as a marker of trophectoderm maturation

[32]. Thus, our study partially answers the above questions. Further studies that combines lineage tracing and genome editing will help us to understand early cell identity and plasticity.

Taken together, our findings not only help us to understand the transcriptional regulation of primates early post-implantation development but also provide valuable resources for regenerative medicine.

Methods

Animals

Healthy cynomolgus monkeys (Macaca fascicularis) of 5 to 12 years old were used in this study. Monkeys were usually housed in groups, while during superovulation, oocyte collection they were provisionally caged individually at 16–26℃ under 40% to 70% relative humidity and a 08:00 to 20:00 light vs. dark photoperiod, fed of commercial pelleted food and water ad libitum. Vaginal bleeding was observed twice per day to detect the onset of menses. The beginning of bleeding wad defined as the 1st day of menstruation.

Embryo culture and single cell collection

After washing with phosphate buffered saline (PBS) (MA0008, Meilunbio), the embryos were cut into several pieces with a 1-mL syringe and digested into single cells with 0.1% trypsin (25200-072, GIBCO) at 37℃ for 3-5 minutes. After neutralization with 2% FBS (04-002-1A, Biological Industries), the cells were washed with ice-cold PBS containing 0.1-1% BSA. Finally, the individual cells were picked into ice-cold lysis buffer on ice with a mouth pipette for single cell ATAC libraries construction as previously described [5] .

Pre-processing of single-cell RNA-sequencing (scRNA-seq) data

For the pre-processing of raw sequencing data, we removed adapters and filtered out low-quality reads with an N rate > 0.2 using Cutadapt (v1.15) [41]. Filtered reads were then aligned to the *Macaca fascicularis* genome (Macaca_fascicularis_5.0) using STAR (v2.5.3) [42]. Rsem-Calculate-Expression (RSEM) (v1.3.0) [43] was used to calculate the read counts, which were then quantified as transcripts per million

mapped reads (TPM). Cells with mapped reads < 1 million and ≤ 2000 genes with TPM values > 1 were filtered out.

Cell clustering and uniform manifold approximation and projection (UMAP) projection

We selected the top 2,000 variable genes based on log-transformed TPM matrices using Seurat (v3.2.2) [17]. Principal components analysis (PCA) was performed, and the first 30 principal components (PCs) were used to build an SNN graph using the "FindNeighbors" function in the R package Seurat. We used UMAP [44] to visualize the distance between the cells on a two-dimensional map. Harmony (v1.0) [45] was used to remove the batch effects between the embryos. Cell clustering was performed using the "FindClusters" function in Seurat and cell-types annotations based on known cell-type specific marker genes. We identified marker genes between the clusters using "FindAllMarkers" function in Seurat ($p < 0.05$). The top 2,000 differentially expressed genes were selected to construct the trajectory model using Monocle (v2.18.0) [46, 47].

Comparisons between EPI cells *in vitro* and *in vivo*

We extracted the overlapping genes in EPI cells between our and *in vivo* embryos [31]. The “IntegrateData” function in Seurat was applied to our and *in vivo* datasets and a UMAP graph was constructed to visualize the similarities between *in vitro* and *in vivo* embryos. Next, we averaged the gene expression values of each cell cluster in both datasets and calculated the Pearson correlations between the cell clusters in both datasets. The Euclidean distance between the cell clusters was calculated and unsupervised hierarchical clustering was performed to determine gene expression pattern similarities between the *in vitro* and *in vivo* cell clusters.

Pre-processing of single-cell ATAC-sequencing (scATAC-seq) data

The raw sequencing data was filtered using Cutadapt (v1.16) [41], and then filtered fragments were aligned to the *Macaca fascicularis* genome (Macaca_fascicularis_5.0) using Bowtie2 (v2.2.5) [48]. Fragments with an alignment quality of > Q30 were retained, and duplicate fragments were removed. We filtered out cells whose usable

fragments were<10,000 and promoter regions (500 bp around the transcriptional start site) with a ratio of fragments <10%. Sambamba (v0.6.6) [49] was used to aggregate the fragments of all cells. Reference peaks were constructed using MACS2 (v2.1.2) [50]. Finally, we counted fragments in the reference peaks using the "getCounts" function in chromVAR (v1.4.0) [25].

scATAC-seq dataset analysis

Signac (v0.2.5) [51] was used to analyze the processed scATAC-seq dataset. We used latent semantic indexing (LSI) and UMAP to reduce the dimensions and visualize for the scATAC-seq dataset (dims = 1:6). Gene activity scores were calculated using the "FeatureMatrix" function to count fragments in the 2 kb-upstream regions of genes and gene bodies. Cell clustering was performed using "FindNeighbors" and "FindClusters" functions in Seurat. Differentially accessible regions were identified using the "FindMarkers" function in Seurat with parameters of min.pct = 0.2 and test.use = 'LR'. The per-cell motif activity score was computed by chromVAR

(“RunChromVAR” function in Signac). Finally, TF motif enrichment of differentially accessible regions was performed by using the “FindMotifs” function.

Integrated analysis of scRNA-seq and scATAC-seq dataset

scATAC-seq and scRNA-seq pairs were matched by Seurat’s canonical correlation analysis (CCA, dims = 1:30) and scRNA-seq cell-type annotations information were transferred to scATAC-seq. As previously described [52], we identified peak-to-gene links based on the null *trans* correlations. Differential peak-to-gene linkages were visualized by ComplexHeatmap (v2.7.8.1000) [53]. Gene Ontology (GO) analysis of the corresponding genes was performed using clusterProfiler (v3.18.1) [54].

For the construction of the transcription factor (TF)-target gene network, we first identified the TFs and marker genes that were highly expressed in the same group of cells. Next, we identified TF-target gene pairs, that is, if the marker gene was linked with peaks that matched the corresponding TF motif. For a given marker gene with at least one linked and matched peak, we summed their squared correlation R^2 as the linkage score for the TF-target pair. NetworkD3 (v0.4)

(https://CRAN.R-project.org/package=networkD3) was used to visualize the TF-target gene network. For subtype analysis of each individual lineage, we reconstructed the TF regulatory network using data from the corresponding lineage.

Analysis of gained and lost peaks

We identified the accessible peaks that (peak read count was > 0) in each stage (the percentage of cells with accessible peaks was > 0.25). The gained peaks at a particular stage were defined as the accessible peaks nonoverlapping with a previous stage. The lost peaks at a particular stage were defined as the peaks non-existing with this stage compared to a previous stage. Gained and lost peak-to-gene linkages were visualized by ComplexHeatmap [52]. GO analysis of the corresponding genes was performed using clusterProfiler [54].

Identification of genes involving in EPI and TE lineage specification

To study the genes with increasing expression differences during lineage differentiation, we identified the differentially expressed genes in EPI and TE

sub-types using the “FindMarkers” function in Seurat and selected genes with increasing log fold change values and -log *P* values. EPI and TE up-regulated genes were classified based on the log fold change values.

Identification of transcription factors that regulates lineage specification

TF motif enrichment analysis was performed for genes that were upregulated in the EPI and TE. The *P* values of EPI-upregulated genes were divided by the *P* values of TE-upregulated genes and subsequently log10-transformed. TFs were classified into EPI and TE groups based on positive and negative of log10 *P* value fold changes. TFs with absolute values of log10 *P* value fold changes greater > 1 and corresponding *P* values < 0.05 were retained.

To examine the importance of TFs in the corresponding group regulatory network, we calculated the degree centrality, closeness centrality and eigenvector centrality of each TF in the network and the rank, respectively. The comprehensive rank was obtained by adding the ranks of the three centralities, and the higher the rank is, the more influential the TFs in the network.

Data Availability

Data and materials availability: All sequencing data were deposited at the National Center for Biotechnology Information Sequence Read Archive (https://www.ncbi.nlm.nih.gov/sra) under accession no. SRP175059. The data were also deposited at the China National GeneBank (CNGB) Nucleotide Sequence Archive (CNSA; https://db.cngb.org/cnsa) under accession no. CNP0000231.

Additional Files

Supplementary Table S1. Quality control data for scATAC-seq dataset.

Supplementary Table S2. Lineage specific marker peaks and corresponding genes.

Supplementary Table S3. Lineage specific TFs and their candidate target genes.

Supplementary Table S4. Gained or lost peaks and corresponding genes during EPI lineages transitions.

Supplementary Table S5. EPI subtype lineage specific TFs and corresponding target genes.

Supplementary Table S6. Trophoblast subtype lineage specific TFs and corresponding target genes.

Supplementary Table S7. Gained or lost peaks and corresponding genes during trophoblast lineages transitions.

Supplementary Table S8. Gene list of 220 genes involving in EPI and trophoblast lineage segregation.

Supplementary Fig. S1. Quality control of scATAC-seq data. (A) Bar charts showing distribution of embryonic day in each cell types. Left panel, scATAC-seq dataset; right panel, scRNA-seq dataset. (B) Violin chart showing the quality control for each embryo. (C) Coembedded UMAP of scATAC-seq and scRNA-seq data with cells colored by technology, cell type or embryonic day. (D) UMAP plot of gene expression levels and motif deviation scores of lineage specific TFs.

Supplementary Fig. S2. Dynamics of EPI cell fate transitions. (A) Hierarchical cluster analysis of expression profiles of EPI subtypes between *in vivo* and *in vitro* embryos. (B) Bar chart showing number of genes which gained or lost peaks during EPI subtypes transitions. Genes are grouped by numbers of peaks changed. (C) The

number of accessible correlated peaks changed for each gene during EPI subtypes transitions. (D) Heatmap showing gained peaks and corresponding genes during EPI subtypes transitions with listed well-studied marker genes, TF binding motifs, candidate TFs, and enriched GO terms. *P* values derived from the hypergeometric test are shown, and the color indicates the gene ratios. (E) Heatmap showing lost peaks and corresponding genes during EPI subtypes transitions with listed well-studied marker genes, TF binding motifs, candidate TFs, and enriched GO terms. *P* values derived from the hypergeometric test are shown, and the color indicates the gene ratio.

Supplementary Fig. S3. Dynamics of trophoblast cell fate transitions. (A) Pseudotime trajectory of trophoblast lineages with cells colored by trophoblast subtypes and pseudotime. (B) Heatmaps showing z-scores of trophoblast averaged subtype specific TFs, their target gene expression levels and gene activity scores. Averaged gene expressions levels and gene activity scores are calculated from cells aggregated by trophoblast subtypes. (C) The number of changed accessible peaks of each gene during transition of trophoblast subtypes. The genes are ranked by changed

accessible peaks. (D) Heatmap showing gained peaks and corresponding genes during each trophoblast subtypes transitions with listed well-studied marker genes, TF binding motifs, corresponding candidate TFs, and representative enriched GO terms. *P* values derived from the hypergeometric test are shown, and the color indicates the gene ratio. (E) Heatmap showing lost peaks and corresponding genes during each trophoblast subtypes transitions with listed well-studied marker genes, TF binding motifs, corresponding candidate TFs, and representative enriched GO terms. *P* values derived from the hypergeometric test are shown, and the color indicates the gene ratio.

Supplementary Fig. S4. Transcriptional regulation of EPI and trophoblast lineage segregation. (A) Heatmap showing Pearson correlation coefficient representing EPI and TE subtype expression profiles and subtypes were classified into four groups based on development stage and hierarchical clustering (pre_EPI_TE, post_TE, post_EP and Gast). pre, pre-implantation; post, post-implantation; Gast, gastrulating cells. (B) Heatmap showing Pearson correlation coefficient representing EPI and TE subtypes chromatin accessibility profiles, subtypes were classified four groups

(pre_EPI_TE, post_TE, post_EP and Gast). pre, pre-implantation; post, post-implantation; Gast, gastrulating cells. (C) Heatmap showing TF expression and corresponding motif enrichments which regulate lineage differentiation. Expression levels were transformed to z-scores and *P* values are derived from the hypergeometric test.

Ethics Approval

All programs were approved by the Institutional Animal Care and Use Committee (IACUC) of Yunnan Key Laboratory of Primate Biomedical Research.

Competing Interests

The authors declare no competing interests.

Funding

This work was supported by the National Natural Science Foundation of China (82192871), the Natural Science Foundation of Yunnan Province (202001BC070001 and 202102AA100053), and the China National GeneBank (CNGB).

Authors' contributions

H.S., N.S., C.L., L.W., Y.Y., X.W. and L.L. performed most of the experiments. X.D., B.C. and B.B. performed the bioinformatics analysis. X.D., J.W, H.Y., W.J., Z.S. and T.T. participated in discussions. X.D., J. W., Z.S., and T.T. analyzed the data and wrote the manuscript. T.T. and Z.S. conceived and supervised the study.

References

1. Rossant J and Tam PPL. Early human embryonic development: Blastocyst formation to gastrulation. Dev Cell. 2022;57 2:152-65. doi:10.1016/j.devcel.2021.12.022.

2. Zhai J, Xiao Z, Wang Y and Wang H. Human embryonic development: from peri-implantation to gastrulation. Trends Cell Biol. 2022;32 1:18-29.

doi:10.1016/j.tcb.2021.07.008.

3. Xiang L, Yin Y, Zheng Y, Ma Y, Li Y, Zhao Z, et al. A developmental landscape of 3D-cultured human pre-gastrulation embryos. Nature. 2020;577 7791:537-42. doi:10.1038/s41586-019-1875-y.

4. Ma H, Zhai J, Wan H, Jiang X, Wang X, Wang L, et al. In vitro culture of cynomolgus monkey embryos beyond early gastrulation. Science. 2019;366 6467 doi:10.1126/science.aax7890.

5. Niu Y, Sun N, Li C, Lei Y, Huang Z, Wu J, et al. Dissecting primate early post-implantation development using long-term in vitro embryo culture. Science. 2019;366 6467 doi:10.1126/science.aaw5754.

6. Deglincerti A, Croft GF, Pietila LN, Zernicka-Goetz M, Siggia ED and Brivanlou AH. Self-organization of the in vitro attached human embryo. Nature. 2016;533 7602:251-4. doi:10.1038/nature17948.

7. Shahbazi MN, Jedrusik A, Vuoristo S, Recher G, Hupalowska A, Bolton V, et al. Self-organization of the human embryo in the absence of maternal tissues. Nat Cell Biol. 2016;18 6:700-8. doi:10.1038/ncb3347.

8. Xiang Y, Zhang Y, Xu Q, Zhou C, Liu B, Du Z, et al. Epigenomic analysis of gastrulation identifies a unique chromatin state for primed pluripotency. Nat Genet. 2020;52 1:95-105. doi:10.1038/s41588-019-0545-1.

9. Yang X, Hu B, Liao J, Qiao Y, Chen Y, Qian Y, et al. Distinct enhancer signatures in the mouse gastrula delineate progressive cell fate continuum during embryo development. Cell Res. 2019;29 11:911-26. doi:10.1038/s41422-019-0234-8.

10. Argelaguet R, Clark SJ, Mohammed H, Stapel LC, Krueger C, Kapourani CA, et al. Multi-omics profiling of mouse gastrulation at single-cell resolution. Nature. 2019;576 7787:487-91. doi:10.1038/s41586-019-1825-8.

11. Ke Y, Xu Y, Chen X, Feng S, Liu Z, Sun Y, et al. 3D Chromatin Structures of Mature Gametes and Structural Reprogramming during Mammalian Embryogenesis. Cell. 2017;170 2:367-81 e20. doi:10.1016/j.cell.2017.06.029.

12. Zheng H, Huang B, Zhang B, Xiang Y, Du Z, Xu Q, et al. Resetting Epigenetic Memory by Reprogramming of Histone Modifications in Mammals. Mol

Cell. 2016;63 6:1066-79. doi:10.1016/j.molcel.2016.08.032.

13. Wang C, Liu X, Gao Y, Yang L, Li C, Liu W, et al. Reprogramming of H3K9me3-dependent heterochromatin during mammalian embryo development. Nat Cell Biol. 2018;20 5:620-31. doi:10.1038/s41556-018-0093-4.

14. Shahbazi MN and Zernicka-Goetz M. Deconstructing and reconstructing the mouse and human early embryo. Nat Cell Biol. 2018;20 8:878-87. doi:10.1038/s41556-018-0144-x.

15. Mole MA, Weberling A and Zernicka-Goetz M. Comparative analysis of human and mouse development: From zygote to pre-gastrulation. Curr Top Dev Biol. 2020;136:113-38. doi:10.1016/bs.ctdb.2019.10.002.

16. Yockey LJ and Iwasaki A. Interferons and Proinflammatory Cytokines in Pregnancy and Fetal Development. Immunity. 2018;49 3:397-412. doi:10.1016/j.immuni.2018.07.017.

17. Stuart T, Butler A, Hoffman P, Hafemeister C, Papalexi E, Mauck WM, 3rd, et al. Comprehensive Integration of Single-Cell Data. Cell. 2019;177

7:1888-902 e21. doi:10.1016/j.cell.2019.05.031.

18. Shen H, Yang M, Li S, Zhang J, Peng B, Wang C, et al. Mouse totipotent stem cells captured and maintained through spliceosomal repression. Cell. 2021;184 11:2843-59 e20. doi:10.1016/j.cell.2021.04.020.

19. Messmer T, von Meyenn F, Savino A, Santos F, Mohammed H, Lun ATL, et al. Transcriptional Heterogeneity in Naive and Primed Human Pluripotent Stem Cells at Single-Cell Resolution. Cell Rep. 2019;26 4:815-24 e4. doi:10.1016/j.celrep.2018.12.099.

20. Strumpf D, Mao CA, Yamanaka Y, Ralston A, Chawengsaksophak K, Beck F, et al. Cdx2 is required for correct cell fate specification and differentiation of trophectoderm in the mouse blastocyst. Development. 2005;132 9:2093-102. doi:10.1242/dev.01801.

21. Sritanaudomchai H, Sparman M, Tachibana M, Clepper L, Woodward J, Gokhale S, et al. CDX2 in the formation of the trophectoderm lineage in primate embryos. Dev Biol. 2009;335 1:179-87. doi:10.1016/j.ydbio.2009.08.025.

22. Okae H, Toh H, Sato T, Hiura H, Takahashi S, Shirane K, et al. Derivation of Human Trophoblast Stem Cells. Cell Stem Cell. 2018;22 1:50-63 e6. doi:10.1016/j.stem.2017.11.004.

23. Dong C, Beltcheva M, Gontarz P, Zhang B, Popli P, Fischer LA, et al. Derivation of trophoblast stem cells from naive human pluripotent stem cells. Elife. 2020;9 doi:10.7554/eLife.52504.

24. Telugu BP, Adachi K, Schlitt JM, Ezashi T, Schust DJ, Roberts RM, et al. Comparison of extravillous trophoblast cells derived from human embryonic stem cells and from first trimester human placentas. Placenta. 2013;34 7:536-43. doi:10.1016/j.placenta.2013.03.016.

25. Schep AN, Wu B, Buenrostro JD and Greenleaf WJ. chromVAR: inferring transcription-factor-associated accessibility from single-cell epigenomic data. Nat Methods. 2017;14 10:975-8. doi:10.1038/nmeth.4401.

26. Ma S, Zhang B, LaFave LM, Earl AS, Chiang Z, Hu Y, et al. Chromatin Potential Identified by Shared Single-Cell Profiling of RNA and Chromatin. Cell. 2020;183 4:1103-16 e20. doi:10.1016/j.cell.2020.09.056.

27. Varberg KM, Iqbal K, Muto M, Simon ME, Scott RL, Kozai K, et al. ASCL2 reciprocally controls key trophoblast lineage decisions during hemochorial placenta development. Proc Natl Acad Sci U S A. 2021;118 10 doi:10.1073/pnas.2016517118.

28. Baczyk D, Drewlo S, Proctor L, Dunk C, Lye S and Kingdom J. Glial cell missing-1 transcription factor is required for the differentiation of the human trophoblast. Cell Death Differ. 2009;16 5:719-27. doi:10.1038/cdd.2009.1.

29. Guo G, Stirparo GG, Strawbridge SE, Spindlow D, Yang J, Clarke J, et al. Human naive epiblast cells possess unrestricted lineage potential. Cell Stem Cell. 2021;28 6:1040-56 e6. doi:10.1016/j.stem.2021.02.025.

30. Io S, Kabata M, Iemura Y, Semi K, Morone N, Minagawa A, et al. Capturing human trophoblast development with naive pluripotent stem cells in vitro. Cell Stem Cell. 2021;28 6:1023-39 e13. doi:10.1016/j.stem.2021.03.013.

31. Nakamura T, Okamoto I, Sasaki K, Yabuta Y, Iwatani C, Tsuchiya H, et al. A developmental coordinate of pluripotency among mice, monkeys and

humans. Nature. 2016;537 7618:57-62. doi:10.1038/nature19096.

32. Meistermann D, Bruneau A, Loubersac S, Reignier A, Firmin J, Francois-Campion V, et al. Integrated pseudotime analysis of human pre-implantation embryo single-cell transcriptomes reveals the dynamics of lineage specification. Cell Stem Cell. 2021;28 9:1625-40 e6. doi:10.1016/j.stem.2021.04.027.

33. Peng G and Jing N. The genome-wide molecular regulation of mouse gastrulation embryo. Sci China Life Sci. 2017;60 4:363-9. doi:10.1007/s11427-016-0285-3.

34. Parfitt DE and Shen MM. From blastocyst to gastrula: gene regulatory networks of embryonic stem cells and early mouse embryogenesis. Philos Trans R Soc Lond B Biol Sci. 2014;369 1657 doi:10.1098/rstb.2013.0542.

35. Tam PP and Behringer RR. Mouse gastrulation: the formation of a mammalian body plan. Mech Dev. 1997;68 1-2:3-25. doi:10.1016/s0925-4773(97)00123-8.

36. Ranzoni AM, Tangherloni A, Berest I, Riva SG, Myers B, Strzelecka PM, et al.

Integrative Single-Cell RNA-Seq and ATAC-Seq Analysis of Human Developmental Hematopoiesis. Cell Stem Cell. 2021;28 3:472-87 e7. doi:10.1016/j.stem.2020.11.015.

37. Yu L, Wei Y, Duan J, Schmitz DA, Sakurai M, Wang L, et al. Blastocyst-like structures generated from human pluripotent stem cells. Nature. 2021;591 7851:620-6. doi:10.1038/s41586-021-03356-y.

38. Fan Y, Min Z, Alsolami S, Ma Z, Zhang E, Chen W, et al. Generation of human blastocyst-like structures from pluripotent stem cells. Cell Discov. 2021;7 1:81. doi:10.1038/s41421-021-00316-8.

39. Kagawa H, Javali A, Khoei HH, Sommer TM, Sestini G, Novatchkova M, et al. Human blastoids model blastocyst development and implantation. Nature. 2022;601 7894:600-5. doi:10.1038/s41586-021-04267-8.

40. Liu X, Tan JP, Schroder J, Aberkane A, Ouyang JF, Mohenska M, et al. Modelling human blastocysts by reprogramming fibroblasts into iBlastoids. Nature. 2021;591 7851:627-32. doi:10.1038/s41586-021-03372-y.

41. Martin M. Cutadapt removes adapter sequences from high-throughput sequencing reads. EMBnet J 2011;17:10-2.

42. Dobin A, Davis CA, Schlesinger F, Drenkow J, Zaleski C, Jha S, et al. STAR: ultrafast universal RNA-seq aligner. Bioinformatics. 2013;29 1:15-21. doi:10.1093/bioinformatics/bts635.

43. Li B and Dewey CN. RSEM: accurate transcript quantification from RNA-Seq data with or without a reference genome. BMC Bioinformatics. 2011;12:323. doi:10.1186/1471-2105-12-323.

44. Leland McInnes JH, James Melville. Umap: Uniform manifold approximation and projection for dimension reduction. arXiv preprint arXiv. 2018;1802.

45. Korsunsky I, Millard N, Fan J, Slowikowski K, Zhang F, Wei K, et al. Fast, sensitive and accurate integration of single-cell data with Harmony. Nat Methods. 2019;16 12:1289-96. doi:10.1038/s41592-019-0619-0.

46. Qiu X, Hill A, Packer J, Lin D, Ma YA and Trapnell C. Single-cell mRNA quantification and differential analysis with Census. Nat Methods.

2017;14 3:309-15. doi:10.1038/nmeth.4150.

47. Qiu X, Mao Q, Tang Y, Wang L, Chawla R, Pliner HA, et al. Reversed graph embedding resolves complex single-cell trajectories. Nat Methods. 2017;14 10:979-82. doi:10.1038/nmeth.4402.

48. Langmead B and Salzberg SL. Fast gapped-read alignment with Bowtie 2. Nat Methods. 2012;9 4:357-9. doi:10.1038/nmeth.1923.

49. Tarasov A, Vilella AJ, Cuppen E, Nijman IJ and Prins P. Sambamba: fast processing of NGS alignment formats. Bioinformatics. 2015;31 12:2032-4. doi:10.1093/bioinformatics/btv098.

50. Zhang Y, Liu T, Meyer CA, Eeckhoute J, Johnson DS, Bernstein BE, et al. Model-based analysis of ChIP-Seq (MACS). Genome Biol. 2008;9 9:R137. doi:10.1186/gb-2008-9-9-r137.

51. Stuart T, Srivastava A, Madad S, Lareau CA and Satija R. Single-cell chromatin state analysis with Signac. Nat Methods. 2021;18 11:1333-41. doi:10.1038/s41592-021-01282-5.

52. Granja JM, Klemm S, McGinnis LM, Kathiria AS, Mezger A, Corces MR, et al.

Single-cell multiomic analysis identifies regulatory programs in mixed-phenotype acute leukemia. Nat Biotechnol. 2019;37 12:1458-65. doi:10.1038/s41587-019-0332-7.

53. Gu Z, Eils R and Schlesner M. Complex heatmaps reveal patterns and correlations in multidimensional genomic data. Bioinformatics. 2016;32 18:2847-9. doi:10.1093/bioinformatics/btw313.

54. Yu G, Wang LG, Han Y and He QY. clusterProfiler: an R package for comparing biological themes among gene clusters. OMICS. 2012;16 5:284-7. doi:10.1089/omi.2011.0118.

Figure Legends

Figure 1 Landscape of chromatin accessibility during monkey peri- and post-implantation development

(A) Schematic illustration of scATAC sequencing of cynomolgus monkey embryos at different developmental stages. (B) Uniform manifold approximation and projection (UMAP) plot of all the scATAC-sequenced and scRNA-sequenced cells. Cells are

colored by their cell-type annotation. ATAC cell types were transferred from RNA. (C) Aggregated scATAC-seq tracks denoting the chromatin accessibility peaks for the marker genes of each cell type. Peak-to-gene linkages are shown at the bottom and correlations are represented by arcs colored by the correlation score (color scales for both panels are to the right). (D) Heatmap showing differential peaks (DPs) and corresponding differentially expressed genes (DEGs) for each cell type. Some well-studied lineage marker genes are listed. (E) Representative lineage specific transcription factors (TFs) and their binding motifs in DPs. (F) Representative enriched Gene Ontology (GO) terms within each lineage. *P* values derived from the hypergeometric test are shown, and the color indicates the gene ratio.

Figure 2 Transcription factors network of early embryogenesis in the monkey

(A) Z-scores of averaged lineage specific transcription factor (TF) expression and corresponding motif enrichment in the DPs of each lineage (left panel). Z-scores of averaged lineage specific TF target genes expression levels and averaged gene activity scores calculated from cells aggregated by lineage (right panel). Only the top

ten lineage specific TFs of each lineage with the most significant *P* values in terms of the DEGs are shown here. (B) Heatmaps showing the averaged expressions levels and averaged gene activity scores of representative lineage specific TFs. (C) Lineage specific TF regulatory networks and target genes that are regulated by multiple lineage specific TF modules are shown. The representative target genes are listed beside. (D) Top five GO enrichment terms of the corresponding genes listed in (C). (E) FOXH1 target gene activity scores and gene expression levels in epiblast (EPI) and yolk-sac or visceral endoderm (VE/YE) lineages, respectively. All values were min-max normalized. (F) Representative GO enrichment terms of FOXH1 target genes. *P* values derived from the hypergeometric test are shown, and the color indicates the gene ratio.

Figure 3 Transcriptional regulation of EPI specification

(A) Coembedding of scRNA-seq and scATAC-seq data into single uniform manifold approximation and projection (UMAP) plots (middle and right panels). Cells are colored by EPI subtypes or technology. UMAP plotting of scRNA-sequenced cells is

shown in the left panel. Cells are colored by EPI subtypes. (B) Pseudotime trajectory of the EPI subtypes. Cells are colored by the EPI subtypes. (C) Heatmaps showing gene activity scores and gene expression levels of subtype specific DEGs in preceding manner, which were classified into four groups based on hierarchical clustering, and all values were min-max normalized (left panel). Line chart showing the averaged gene activity scores and gene expression levels in heatmap clusters and representative genes are listed (middle panel). Bar chart showing the representative GO enrichment terms of genes in heatmap clusters. *P* values derived from the hypergeometric test are shown, and the color indicates the gene ratio (right panel). (D) Heatmaps showing gene activity scores and gene expression levels of subtype specific DEGs in synchronized manner, which were classified into four groups based on hierarchical clustering, and all values were min-max normalized (left panel). Line chart showing the averaged gene activity scores and gene expression levels in heatmap clusters and representative genes are listed (middle panel). Bar chart showing the representative GO enrichment terms of genes in heatmap clusters. *P* values derived from the hypergeometric test are shown and the color indicates the gene ratio (right panel). (E)

Box chart of averaged EPI-C and gastrulating cell (Gast) specific TF target gene activity scores and expression levels. (F) Heatmap showing the correlation coefficients of gene expression levels among the representative genes. These genes were classified into three groups by hierarchical clustering. They were up-regulated in early EPI (EPI-A and EPI-B), EPI-C and Gast, respectively. (G) Heatmaps showing gene activity scores and gene expression levels corresponding to (F) and all values were min-max normalized.

Figure 4 Chromatin accessibility dynamics of trophoblast specification during monkey early embryonic development

(A) UMAP plot of RNA-sequenced and ATAC-sequenced single cells derived from the trophoblast lineage, with cells colored by trophoblast subtypes. (B) Heatmaps showing averaged gene activity scores and expression levels of representative marker genes. The color bar represented the z-scores, which were calculated from the trophoblast (TE) subtypes aggregates. A gradient of blue, gray, and red indicates low to high values. (C) TF regulatory networks of each trophoblast subtype are shown.

Gray dots indicate target genes, colored dots indicate trophoblast subtype specific TFs and lines indicate regulatory relationships between TFs and target genes. (D) Bar chart showing gene numbers, with gains or loses peaks during trophoblast specification and 1, 2, and ≥3 indicating peak change numbers. (E) Number of significantly correlated peaks ($p < 0.05$) for each gene (±25 kb from transcription start sites (TSSs)). Domains of regulatory chromatin (DORC) genes situated above the dotted line are shown. (F) Changes in peak numbers and expression levels of genes during trophoblast specification from TE-A to TE-B (A2B), TE-B to TE-C (B2C), and TE-B to TE-D (B2D) are shown. The x-axis indicates the averaged log fold change between the later stage and the earlier stage, and the y-axis indicates the corresponding number change of the accessible peak. (G) Accessible peak numbers, and expression levels of upregulated DORC genes and all values were min-max normalized. (H) Bar chart showing representative GO terms enrichment in (G). *P* values derived from the hypergeometric test are shown, and the color indicates the gene ratio.

Figure 5 Integrative scRNA-Seq and scATAC-Seq analysis reveals epiblast and trophoblast segregation

(A) Pearson correlation coefficient network of EPI and trophoblast subtype expression profiles. Subtypes were classified into four groups based on developmental stage and hierarchical clustering. Line width represents the coefficient correlation and the lines with a coefficient correlation < 0.7 were removed. preE, pre-implantation early; preL, pre-implantation late; pre, pre-implantation; post, post-implantation; pa, parietal. (B) Averaged log fold change (top) and -log10 (*P* value) (bottom) of DEGs in the EPI and trophoblast from peri- to post-implantation of monkey embryos *in vivo* and *in vitro*. The x-axis indicates the ranked gene list, which was ordered by the averaged log fold change between EPI-A and TE-A, EPI-B and EPI-C and TE-C and TE-D, respectively, in *in vitro* cultured embryos, and between ICM and preE-TE, pre-EPI and preL-TE, and post-implantation EPI (postE-EPI, postL-EPI and Gast1) and post-implantation TE (post-paTE) in *in vivo* embryos [31]. preE, pre-implantation early; preL, pre-implantation late; pre, pre-implantation; post, post-implantation; pa, parietal. (C) Venn diagram showing genes involved in EPI and trophoblast segregation. The 220

overlapping genes were conserved genes between embryos *in vivo* and *in vitro*. (D) Weighting the importance of the 220 genes in epiblast and trophoblast segregation. Dot chart showing gene expression level difference ratios and chromatin accessibility difference ratios in the 220 genes in **c**. The ratios of the gene expression levels, or chromatin accessibility differences were expressed as the post-implantation stage log fold change to the pre-implantation stage log fold change. Dot color indicates how many TFs potentially regulated a specific gene. (E) Representative enriched GO terms of 220 genes in the EPI and trophoblast. *P* values derived from the hypergeometric test are shown, and the color indicates the gene ratio. (F) Representative TFs that putatively regulate the segregation of the EPI and trophoblast. All expression levels were min-max normalized, and the motif enrichment were z-scores. (G) Weighting the ranking of the TFs that putatively regulate the expression of the 220 EPI and trophoblast lineage segregation genes.

Figure 1

Click here to access/download;Figure;fig1.jpg

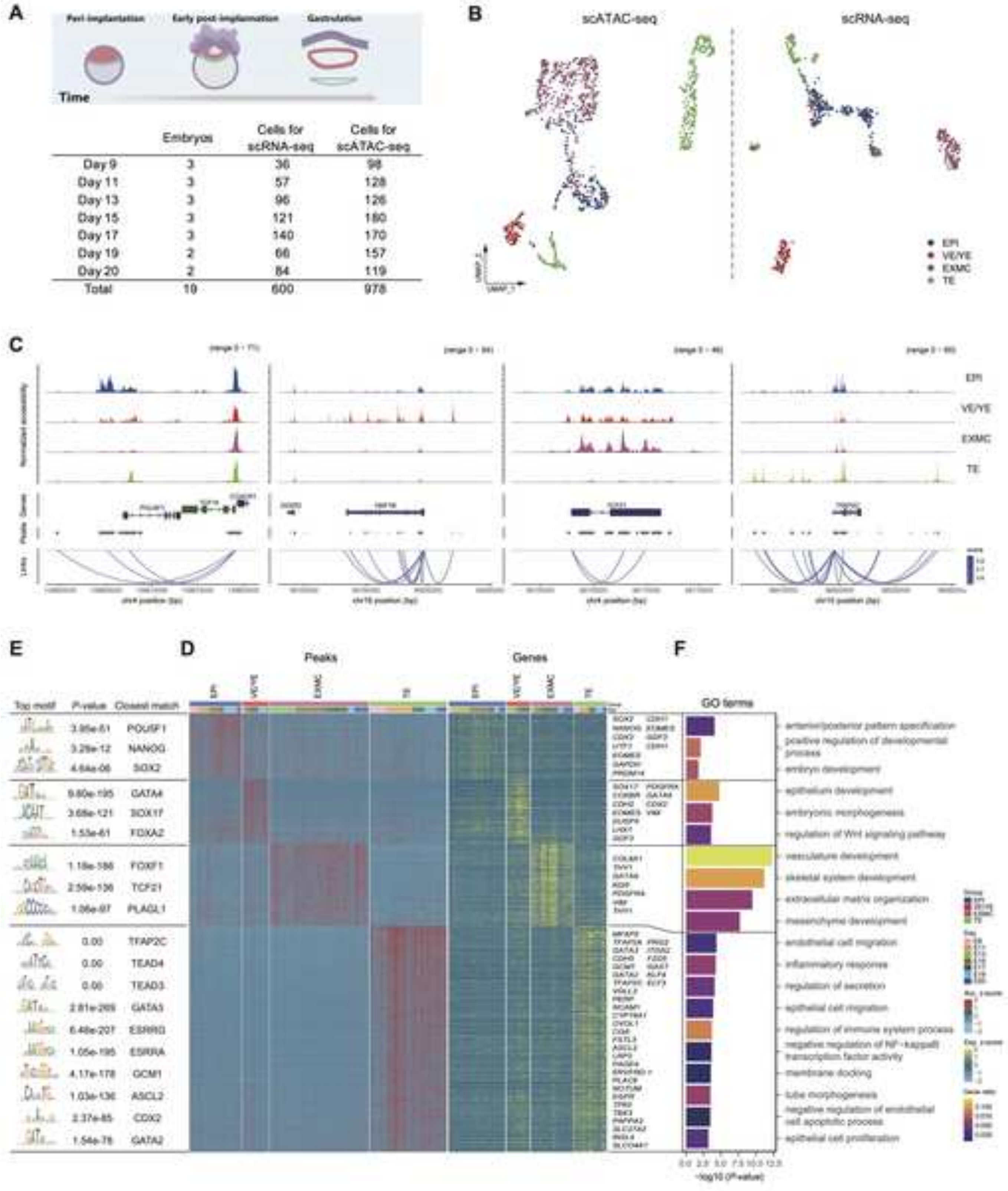

Click here to access/download;Figure;fig2.jpg

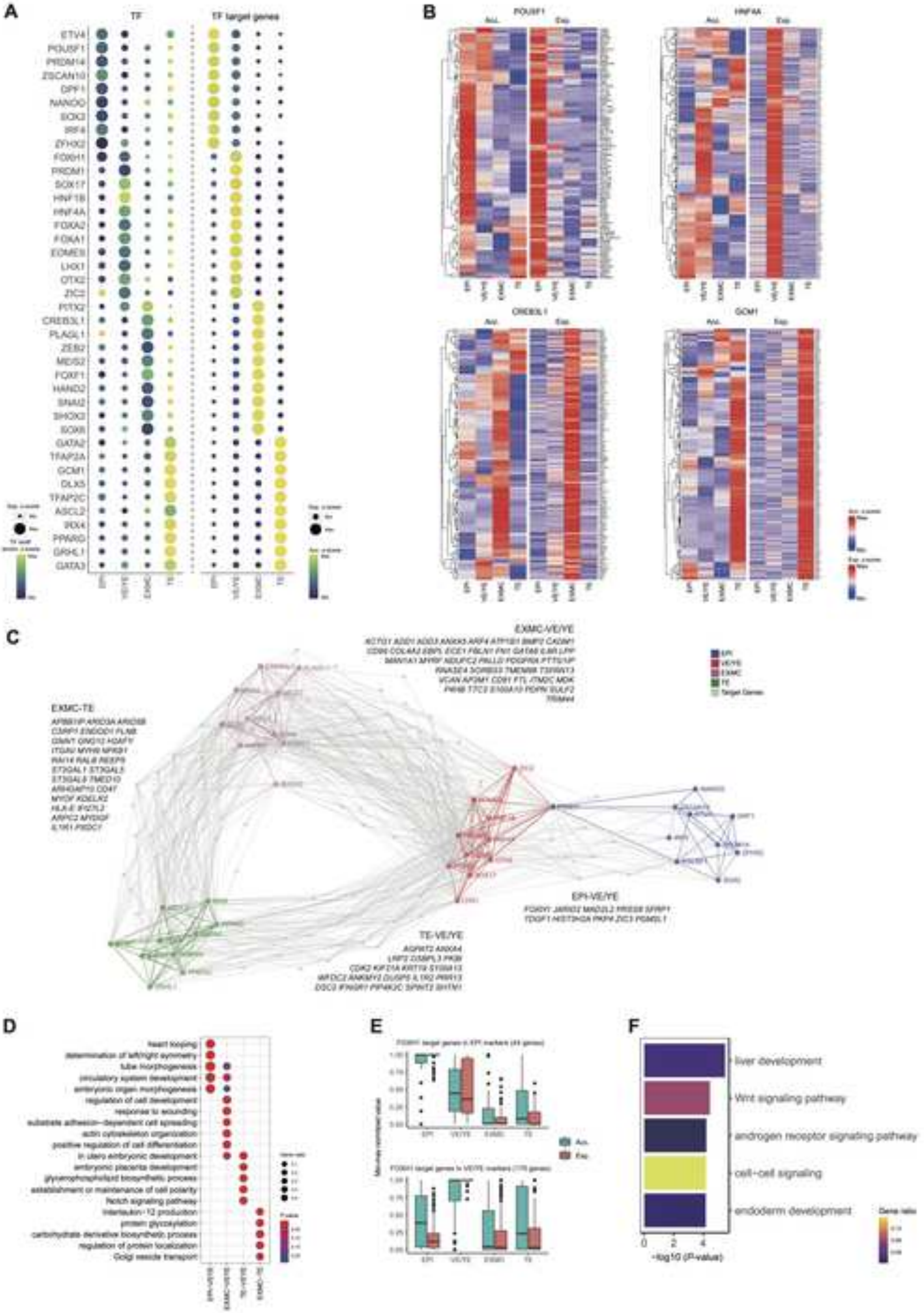

Figure 3

Click here to access/download;Figure;fig3.jpg

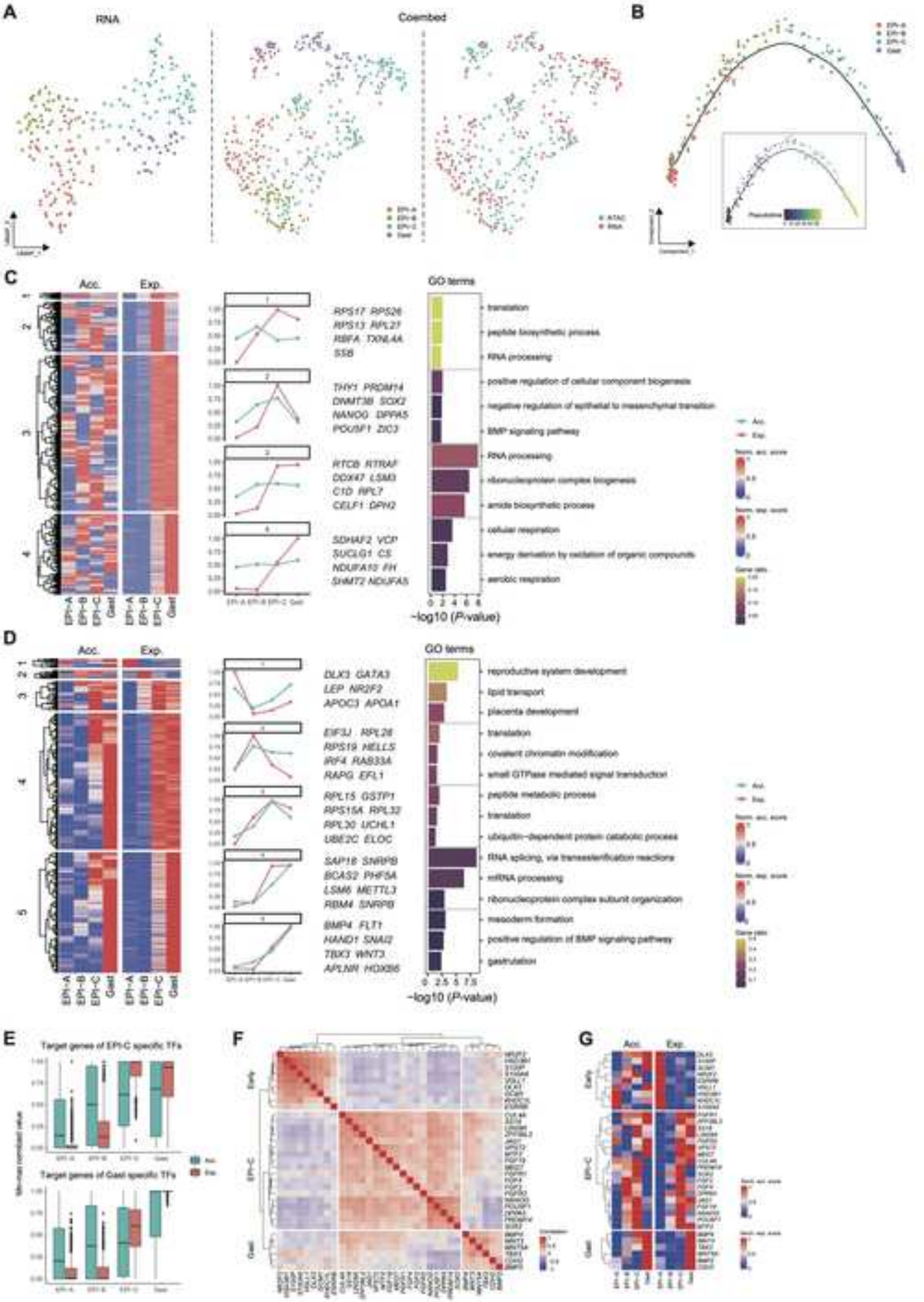

Figure 4 Click here to access/download;Figure;fig4.jpg

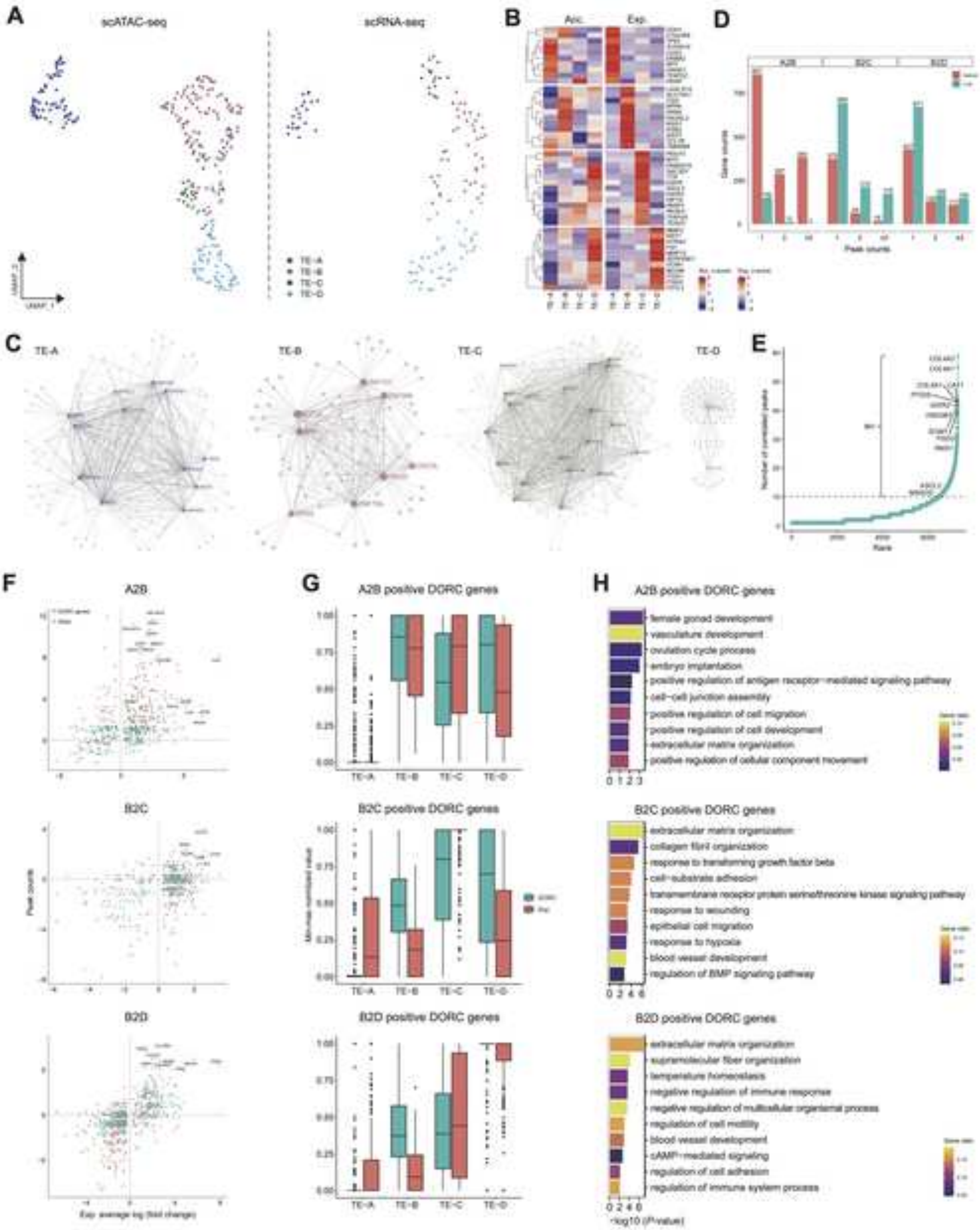

Figure 5

Click here to access/download;Figure;fig5.jpg

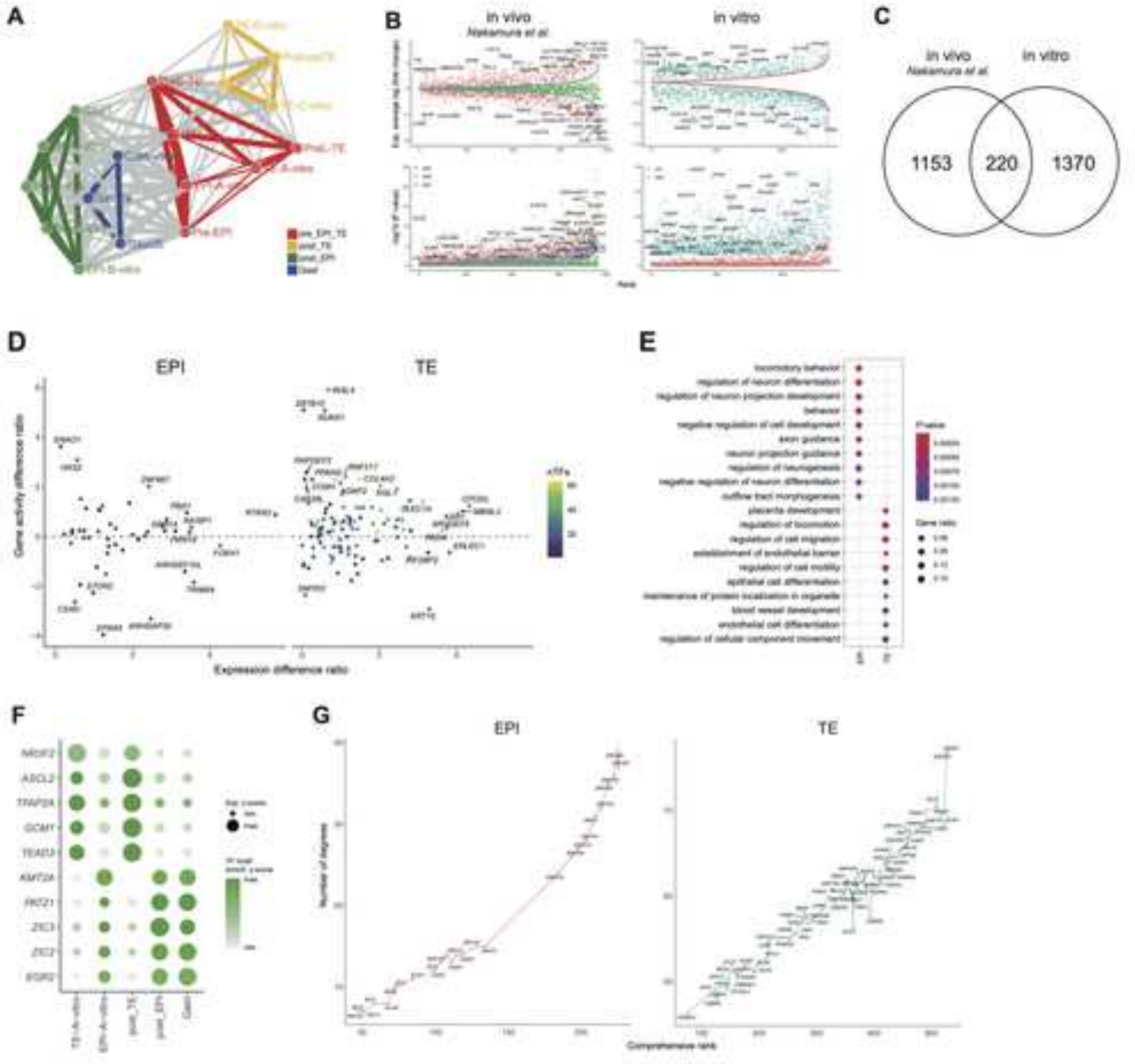

Supplementary Fig. S1

Click here to access/download
Supplementary Material
figS1.jpg

Supplementary Fig. S2

Click here to access/download
Supplementary Material
figS2.jpg

Supplementary Fig. S3

Click here to access/download
Supplementary Material
figS3.jpg

Supplementary Fig. S4

Click here to access/download
Supplementary Material
figS4.jpg

Supplementary Table S1

Click here to access/download
Supplementary Material
TableS1.xlsx

Supplementary Table S2

Click here to access/download
Supplementary Material
TableS2.xlsx

Supplementary Table S3

Click here to access/download
Supplementary Material
TableS3.xlsx

Supplementary Table S4

Click here to access/download
Supplementary Material
TableS4.xlsx

Supplementary Table S5

Click here to access/download
Supplementary Material
TableS5.xlsx

Supplementary Table S6

Click here to access/download
Supplementary Material
TableS6.xlsx

Supplementary Table S7

Click here to access/download
Supplementary Material
TableS7.xlsx

Supplementary Table S8

Click here to access/download
Supplementary Material
TableS8.xlsx
