# Supplementary material for: Developmental dynamics of chromatin accessibility during post-implantation development of monkey embryos
Source: Gigascience. 2023 May 25;12:giad038. doi: 10.1093/gigascience/giad038 (PMC10209733; doi:10.1093/gigascience/giad038)
Supplement: giad038_GIGA-D-22-00278_Revision_1 [file giad038_giga-d-22-00278_revision_1.pdf]

GigaScience

Developmental dynamics of chromatin accessibility during post-implantation development of monkey embryos

--Manuscript Draft--

**Manuscript Number:**	GIGA-D-22-00278R1
**Full Title:**	Developmental dynamics of chromatin accessibility during post-implantation development of monkey embryos
**Article Type:**	Research
**Funding Information:**	National Natural Science Foundation of China (82192871) — Dr. Tao Tan<br>Natural Science Foundation of Yunnan Province (202001BC070001 and 202102AA100053) — Dr. Tao Tan<br>the China National GeneBank — Dr. Zhouchun Shang
**Abstract:**	Background: Early post-implantation development, especially gastrulation in primates, is accompanied by extensive drastic chromatin reorganization, which remains largely elusive.<br>Results: To delineate the global chromatin landscape and understand the molecular dynamics during this period, a single-cell assay for transposase accessible chromatin sequencing (scATAC-seq) was applied to in vitro cultured cynomolgus monkey (Macaca fascicularis, hereafter as monkey) embryos to investigate the chromatin status. Firstly, we delineated the cis-regulatory interactions and identified the regulatory networks and critical transcription factors involved in the epiblast (EPI), hypoblast, and trophectoderm/trophoblast (TE) lineage specification. Secondly, we observed that the chromatin opening of some genome regions preceded the gene expression during EPI and trophoblast specification. Thirdly, we identified the opposing roles of FGF and BMP signaling in pluripotency regulation during EPI specification. Finally, we revealed the similarity between EPI and TE in gene expression profiles and demonstrated that PATZ1 and NR2F2 were involved in EPI and trophoblast specification during monkey post-implantation development.<br>Conclusions: Our findings provide a useful resource and insights into dissecting the transcriptional regulatory machinery during primate post-implantation development.
**Corresponding Author:**	Tao Tan<br>State Key Laboratory of Primate Biomedical Research<br>Kunming, CHINA
**Corresponding Author Secondary Information:**	
**Corresponding Author's Institution:**	State Key Laboratory of Primate Biomedical Research
**Corresponding Author's Secondary Institution:**	
**First Author:**	Tao Tan
**First Author Secondary Information:**	
**Order of Authors:**	Tao Tan<br>Xi Dai<br>Honglian Shao<br>Nianqin Sun<br>Baiquan Ci<br>Jun Wu<br>Chuanyu Liu

Powered by Editorial Manager® and ProduXion Manager® from Aries Systems Corporation

	Liang Wu
	Yue Yuan
	Xiaoyu Wei
	Huanming Yang
	Longqi Liu
	Weizhi Ji
	Bing Bai
	Zhouchun Shang
**Order of Authors Secondary Information:**	
**Response to Reviewers:**	Dear Dr. Hongling,<br><br>I am attaching our revised manuscript (GIGA-D-22-00278) titled "Developmental dynamics of chromatin accessibility during post-implantation development of monkey embryos". We thank you and the reviewers for the positive comments and suggestions, which helped improve our manuscript immensely. We have provided a detailed point-by-point response and revised the manuscript to address the remaining concerns.<br><br>If you have any questions, please do not hesitate to contact us.<br><br>Tao Tan<br>State Key Laboratory of Primate Biomedical Research<br>Institute of Primate Translational Medicine<br>Kunming University of Science and Technology<br><br>Our responses to the reviewers' comments<br><br>Referee #1:<br><br>The authors present a scATAC-seq dataset of monkey embryo development. A major limitation of the analysis is that the authors seem to pool cells within each cell type instead directly doing analysis on a single-cell level, which largely loses the power and resolution of single-cell analysis. Below are my specific comments:<br><br>[Response] We greatly thank the reviewer for the constructive comments and agree that analysis on the single-cell level is necessary. We have added the single-cell level analysis in Supplementary Fig. S1C-1G, Fig. 3A-3B, Supplementary Fig. S2A, Supplementary Fig. S3, Supplementary Fig. S4A, S4B, and S4D based on the reviewer's suggestion.<br><br>Fig 1d: What is each row? Is each row showing one DP and its corresponding gene, or one DEG and its corresponding peak? Or are there duplicated genes or peaks in the plot?<br><br>[Response] We have added the descriptions in the figure legend of Fig 1D to clarify this ambiguity. Each row shows one DP with its corresponding gene, whereas some genes are duplicated in a row cluster as multiple peaks link to one gene. (Page 50, lines 880-882).<br><br>For the single-cell analysis, the authors claim that ' which implies that there is a time lag between gene expression and chromatin accessibility during EPI cell specification'. However, this may not be true biology since the scRNA and scATAC were computationally aligned. The time lag could be introduced by the computational alignment.<br><br>[Response] We thank the reviewer for raising this point. In our bioinformatic analysis approach, similar clusters were aligned between scRNA-seq and scATAC-seq datasets based on the top 2,000 variable genes, and high consistency between matched clusters was observed (also see Methods) (Page 29, lines 457-462), implying similar clusters of scRNA-seq and scATAC-seq datasets were well aligned (Fig. 3A).

Powered by Editorial Manager® and ProduXion Manager® from Aries Systems Corporation

Based on this observation, the asynchrony features instead of the congruent features in the aligned clusters are plausible. To further clarify the possibility that the computational alignment could introduce the time lag, we have aligned single ATAC and RNA cells in the revised manuscript based on a geodesic distance-based pairing approach using “FigR” which can reduce the deviation caused by cell cluster mean value to a certain extent (Kartha et al., 2022) (Fig.3A). Notably, a time lag between gene expression and chromatin accessibility during EPI cell specification was also observed (Supplementary Fig. S3A). We also have toned down this claim as “which implies an inconsistency between gene expression and chromatin accessibility during EPI cell specification” in the revised manuscript (Page 12, lines 184-185).

For the pattern analysis in Figure 3C,D, why not use the pseudotime ordering and perform the analysis on a single-cell level, instead of the current analysis that pools cells with the same cell type

[Response] We have aligned the nearest scATAC and scRNA cells by the “pairCells” function in the R package FigR (Kartha et al., 2022) based on co-embedding PCA components, and pairs with the same cell types were retained. The pseudotime ordering of scATAC-seq and scRNA-seq cells have been included in Supplementary Fig. S3A.

Patterns in Figure 3C,D are rather noisy. It is not convincing that there are only two patterns. For example, in C, there are many peaks showing decreasing then increasing pattern, and it is not sure when the open chromatin leads to the increase of gene expression. In D, there are peaks that have increased accessibility after increase of gene expression (in cluster 4 for example). Those may not be called to have synchronized pattern since there is a time lag.

[Response] We agree with the reviewer’s comments; the description of the dynamic gene expression patterns and chromatin accessibility during EPI specification needed to clarify. We have rewritten this part as “To interrogate the correlation between gene expression and chromatin accessibility, we related the DPs to the DEGs, and two main patterns were detected:1) chromatin became accessible first in EPI-A cells, and then the genes were expressed (pattern 1) (Fig. 3C); 2) the scaled values of gene expressions and chromatin accessibility were comparable in EPI-A cells (Fig. 3D) (pattern 2).” (Page 12, lines 189-193). To further delineate the developmental dynamics of gene expression and chromatin accessibility alongside pseudotime ordering of EPI specification, we have paired single cells of scRNA-seq and scATAC-seq datasets, and the patterns of correlation between gene expression and chromatin accessibility were determined (Supplementary Fig. S3A).

Authors should consider comparing the pseudotime analysis by Monocle 2 with other pseudotime analysis methods such as Slingshot and TSCAN.”

[Response] We have compared the pseudotime trajectory created by Slingshot (Street et al., 2018) and TSCAN (Ji and Ji, 2016) with that of Monocle (v2.18.0) (Qiu et al., 2017), and the same developmental trajectories were observed between this three methods (Fig. 3B and Supplementary Fig. S2A)

Similarly for Figure 3E, why not perform analysis on single-cell level using pseudotime ordering?

[Response] We have performed analysis on single-cell level using pseudotime ordering in Supplementary Fig. S3B.

For Figure 4, similar pseudotime analysis on single-cell level should be done.

[Response] As the reviewer suggested, we have paired single TE cells of scRNA-seq and scATAC-seq datasets and created pseudotime trajectory. Then branch-dependent genes whose expression varied with developmental branching were identified, and their chromatin accessibility and expression levels were visualized (Supplementary Fig. S4D).

“FindAllMarkers" function in Seurat ($p < 0.05$) were raw p-value or adjusted p-value

used?

[Response] The adjusted p-values were used.

In the methods section "Comparisons between EPI cells in vitro and in vivo" gene expression values should be scale.

[Response] We have used Seurat (v3.2.2) (Stuart et al., 2019) to remove batch effects from both in vitro and in vivo data, and we used normalized values processed by the "NormalizeData" function according to the tutorial of Seurat. We have added this information in the method section (Page 27, lines 415-416).

In the methods section "Identification of transcription factors that regulates lineage specification": 'The P values of EPI-upregulated genes were divided by the P values of TE-upregulated genes and subsequently log10-transformed.' this does not seem right to me. The pvalues merely reflect the reproducibility of the test and it does not make sense to use p-values as measures of signals. Instead, statistics or fold change should be used in these scenarios.

[Response] As suggested by the reviewer, we have now identified lineage TF based on threshold P value and enrichment fold change. EPI-regulated TFs and TE-regulated TFs have been updated, and corresponding figures were replaced in all figures.

Referee #2:

The proposed manuscript described the analysis of the chromatin accessibility of monkey embryos. This is an important resource that has the potential to increase our knowledge in early embryogenesis of embryos. The analysis is enhanced with previously published single cell RNA-seq data. The computational approach and analysis is sound, but mostly restrict on describing TFs, putative targets and the gene ontology terms. Authors do not include any functional validation or comparative analysis, i.e. contrast of results in other organisms. This would have enhanced the study.

[Response] We thank the reviewer for the positive comments. We have revised the manuscript based on the reviewer's suggestions, and the cross-species comparison is included in Supplementary Fig. S6.

Major points:
It is not clear from figure S1C that coembedding found the similar cell types. More analysis should be done, as for example a comparison of cell proportion of the clusters found in scatac and scrna. Another interesting approach would be to correlate the gene accessibility and gene expression of the equivalent cells.

[Response] Thanks for this insightful comment. As suggested, we have added a bar plot showing the percentage of cell types in scATAC-seq and scRNA-seq datasets and box plots to show the chromatin accessibility and gene expression levels of cell markers in each cell type of ATAC-RNA aligned cells. To correlate the gene accessibility and expression of the equivalent cells, we have now paired scATAC-seq and scRNA-seq cells based on a geodesic distance-based pairing approach using FigR, and the combined patterns of chromatin accessibility and gene expressions have been determined (Supplementary Fig. S1C-S1G)

Similarly, it is hard to interpret Fig. S1D. Authors should show the results in the co-embedding space.

[Response] We have provided the new figures showing the results in the co-embedding space in Supplementary Fig. S1G.

Altogether authors should give more details on how the scRNA/scATAC integration is done. This could also include a short results in the results section (page 7).

[Response] We thank the reviewer for raising this point. As suggested, we have described more details on integrating scRNA-seq and scATAC-seq datasets in the

	results section (page 7, lines 96-106) and methods (page 29, lines 454-476). Furthermore, a histogram plot was provided to show the distribution of prediction scores in annotated label transfer based on CCA, reflecting the integration quality (Supplementary Fig. S1D).<br><br>Reference<br>Ji, Z., and Ji, H. (2016). TSCAN: Pseudo-time reconstruction and evaluation in single-cell RNA-seq analysis. Nucleic Acids Res 44, e117. 10.1093/nar/gkw430.<br>Kartha, V.K., Duarte, F.M., Hu, Y., Ma, S., Chew, J.G., Lareau, C.A., Earl, A., Burkett, Z.D., Kohlway, A.S., Lebofsky, R., and Buenrostro, J.D. (2022). Functional inference of gene regulation using single-cell multi-omics. Cell Genom 2. 10.1016/j.xgen.2022.100166.<br>Qiu, X., Hill, A., Packer, J., Lin, D., Ma, Y.A., and Trapnell, C. (2017). Single-cell mRNA quantification and differential analysis with Census. Nat Methods 14, 309-315. 10.1038/nmeth.4150.<br>Street, K., Risso, D., Fletcher, R.B., Das, D., Ngai, J., Yosef, N., Purdom, E., and Dudoit, S. (2018). Slingshot: cell lineage and pseudotime inference for single-cell transcriptomics. BMC Genomics 19, 477. 10.1186/s12864-018-4772-0.<br>Stuart, T., Butler, A., Hoffman, P., Hafemeister, C., Papalexi, E., Mauck, W.M., 3rd, Hao, Y., Stoeckius, M., Smibert, P., and Satija, R. (2019). Comprehensive Integration of Single-Cell Data. Cell 177, 1888-1902 e1821. 10.1016/j.cell.2019.05.031.
**Additional Information:**	
**Question**	**Response**
Are you submitting this manuscript to a special series or article collection?	No
**Experimental design and statistics**<br><br>Full details of the experimental design and statistical methods used should be given in the Methods section, as detailed in our Minimum Standards Reporting Checklist. Information essential to interpreting the data presented should be made available in the figure legends.<br><br>Have you included all the information requested in your manuscript?	Yes
**Resources**<br><br>A description of all resources used, including antibodies, cell lines, animals and software tools, with enough information to allow them to be uniquely identified, should be included in the Methods section. Authors are strongly encouraged to cite Research Resource Identifiers (RRIDs) for antibodies, model organisms and tools, where possible.	Yes

Powered by Editorial Manager® and ProduXion Manager® from Aries Systems Corporation

Have you included the information requested as detailed in our Minimum Standards Reporting Checklist?	
**Availability of data and materials**<br><br>All datasets and code on which the conclusions of the paper rely must be either included in your submission or deposited in publicly available repositories (where available and ethically appropriate), referencing such data using a unique identifier in the references and in the "Availability of Data and Materials" section of your manuscript.<br><br>Have you have met the above requirement as detailed in our Minimum Standards Reporting Checklist?	Yes

Powered by Editorial Manager® and ProduXion Manager® from Aries Systems Corporation

Manuscript

Click here to access/download;Manuscript;manuscript (1) (1).docx

Developmental dynamics of chromatin accessibility during post-implantation development of monkey embryos

Xi Dai[1, 2, #], Honglian Shao[3, 4, #], Nianqin Sun[3, 4, #], Baiquan Ci[3, 4], Jun Wu[5], Chuanyu Liu[2], Liang Wu[1], Yue Yuan[2], Xiaoyu Wei[2], Huanming Yang[2, 6], Longqi Liu[1, 2], Weizhi Ji[3, 4], Bing Bai[3, 4], Zhouchun Shang[1, 2, 6*], Tao Tan[3, 4, *]

[1] College of Life Sciences, University of Chinese Academy of Sciences, Beijing 100049, China

[2] BGI-Shenzhen, Shenzhen 518083, China

[3]Yunnan Key Laboratory of Primate Biomedical Research, Institute of Primate Translational Medicine, Kunming University of Science and Technology, Kunming, Yunnan 650500, China

[4]Yunnan Key Laboratory of Primate Biomedical Research, Kunming, Yunnan, 650500, China

[5] Department of Molecular Biology, University of Texas Southwestern Medical Center, Dallas, TX 75390, USA

[6]James D. Watson Institute of Genome Sciences, Hangzhou, 310013, China

[#]These authors contributed equally to this work.

[*]Corresponding author. Email: tant@lpbr.cn (T.T.); shangzhouchun@mgi-tech.com (Z.S.).

ORCID iDs:

Tao Tan [0000-0001-8650-0388]; Xi Dai [0000-0002-8240-5487]; Honglian Shao [0000-0002-8758-1834]; Nianqin Sun [0000-0001-9461-3307]; Baiquan Ci [0000-0001-8269-8032]; Jun Wu [0000-0001-9863-1668]; Chuanyu Liu [0000-0003-2258-0897]; Liang Wu [0000-0002-6784-0181]; Xiaoyu Wei [0000-0001-9592-3083]; Huanming Yang [0000-0002-0858-3410]; Longqi Liu [0000-0002-5828-5542]; Weizhi Ji [0000-0003-2550-4224]; Zhouchun Shang [0000-0002-1740-7961].

Abstract

Background: Early post-implantation development, especially gastrulation in

primates, is accompanied by extensive drastic chromatin reorganization, which remains largely elusive.

Results: To delineate the global chromatin landscape and understand the molecular dynamics during this period, a single-cell assay for transposase accessible chromatin sequencing (scATAC-seq) was applied to *in vitro* cultured cynomolgus monkey (*Macaca fascicularis*, hereafter as monkey) embryos to investigate the chromatin status. Firstly, we delineated the cis-regulatory interactions and identified the regulatory networks and critical transcription factors involved in the epiblast (EPI), hypoblast, and trophectoderm/trophoblast (TE) lineage specification. Secondly, we observed that the chromatin opening of some genome regions preceded the gene expression during EPI and trophoblast specification. Thirdly, we identified the opposing roles of FGF and BMP signaling in pluripotency regulation during EPI specification. Finally, we revealed the similarity between EPI and TE in gene expression profiles and demonstrated that PATZ1 and NR2F2 were involved in EPI and trophoblast specification during monkey post-implantation development.

Conclusions: Our findings provide a useful resource and insights into dissecting the

transcriptional regulatory machinery during primate post-implantation development.

Keywords: cynomolgus monkey, ex vivo, gastrulation, scATAC-seq, chromatin dynamics

Background

The transition from pre-implantation to gastrulation represents a milestone of early embryogenesis in primates and involves extensive morphogenesis and lineage specification and differentiation. During this stage, a connection between the embryo and the mother is established, while the trophectoderm (TE) differentiates into cytotrophoblasts (CTs), extravillous cytotrophoblasts (EVTs), and syncytiotrophoblasts (STs); the cavitation of the amnion and yolk sac initiates, and the gastrulation of the embryo launches to form three germ layers and program the body plan of the fetus [1, 2]. However, there are technical limitations and ethical concerns, and the molecular mechanisms underlying this transition remain largely elusive.

Recently, advancements in embryo *in vitro* culture systems have enabled us to

investigate transcriptional and DNA methylation dynamics during the early embryonic development in humans and monkeys [3-7]. However, several key questions, including the chromatin status that underlies this transition, have yet to be addressed.

In the mouse, chromatin accessibility, histone modifications, and 3D chromatin structures during post-implantation development have been extensively studied, and epigenetic regulatory networks have been revealed [8-13]. As significant differences exist between primates and mice in terms of post-implantation development, for example, in the morphogenesis of embryonic and extra-embryonic structures and signaling pathways involved in the specification of embryonic and extra-embryonic lineages [1, 2, 14, 15], the knowledge derived from mouse models could not be straightforwardly extrapolated to primate models. This poses a significant limitation to studies of, for example, the regulation of pluripotent stem cells (PSCs) in primates.

Here, we harness the power of single-cell assay for transposase accessible chromatin sequencing (scATAC-seq) and embryo *in vitro* culture platform to unravel the regulatory chromatin landscape during early embryonic development in monkeys.

This study provides a valuable resource for studying chromatin dynamics and chromatin regulation during early embryonic development in primates.

Results

scATAC-seq profiles of early monkey embryogenesis

To determine the regulatory landscape at single-cell resolution during monkey peri- and post-implantation development, we performed scATAC-seq of cultured monkey embryos from day 9 post-fertilization (9 d.p.f.) to 20 d.p.f. as our previously reported and our published single cell RNA sequencing (scRNA-seq) dataset was included for analysis [5] (Fig. 1A and Supplementary Fig. S1A). In total, 1,198 individual cells were sequenced, and after stringent filtration (usable fragments > 10,000, promoter fragments ratio > 10%), 978 high-quality single nuclei, distributing from 9 d.p.f. and 20 d.p.f. were retained (Supplementary Table S1 and Supplementary Fig. S1A). Cells within per embryo passing filter had median fragments ranging from 20,020 to 61,182, the median fraction of fragments in promoters (500 bp around transcriptional start site) ranged from 12.14% to 19.01%, and the median fraction of fragments in peaks ranged

from 51.37% to 64.39 % (Supplementary Fig. S1B).

Next, based on these high-quality data, we investigated global gene regulatory activities during monkey early development. First, the resting 978 cells were dimensionally reduced using uniform manifold approximation and projection (UMAP) and clustering analysis. In scRNA-seq analysis, four main cell clusters, namely EPI, TE, yolk-sac or visceral endoderm (VE/YE), and extra-embryonic mesenchyme cell (EXMC) were identified (Fig. 1B). To interrogate correspondence between chromatin accessibility and gene expression during early monkey embryogenesis, we then integrated our scATAC-seq and scRNA-seq datasets. Generally, canonical correlation analysis (CCA) and mutual nearest-neighbors (MNNs) algorithms were applied, and the annotated scRNA-seq dataset was used as a reference to annotate the scATAC-seq dataset (see also method). This integrated object yielded consistent overlap between scRNA-seq and scATAC-seq cell types with high integration scores after co-embedding scRNA-seq and scATAC-seq datasets (Supplementary Fig. S1C and S1D). Additionally, the cell proportion (Supplementary Fig. S1E) and the gene expression and chromatin accessibility of lineage markers were comparable

(Supplementary Fig. S1F). These results suggest a strong correlation between chromatin accessibility and gene expression in this integrated analysis.

Next, enrichment of ATAC-seq peaks in promoter and distal regions of lineage markers were identified, including OCT4 locus (also known as POU5F1) in the EPI, TFAP2C in the TE, HNF1B in the VE/YE, and TCF 21 in the EXMCs (Fig. 1C). Furthermore, the binding motifs and the expression of these markers were also enriched in the four major cell clusters (Supplementary Fig. S1G).

We then related the cluster-specific differential peaks (DPs) to the differentially expressed genes (DEGs) (Supplementary Table S2 and Fig. 1D). Furthermore, the enrichment of well-known lineage-specific transcription factors (TFs) binding motifs was observed for cluster-specific DPs, such as OCT4 (POU5F1) and NANOG in the EPI, TFAP2C, and TEAD4 in the TE, GATA4 in the VE/YE, and TCF21 and FOXF1 in EXMCs (Fig. 1E). Gene Ontology (GO) term enrichment analysis of DEG related DPs revealed that the early-development associated terms such as anterior/posterior pattern specification and embryo development were enriched in the EPI; the most enriched GO terms in the VE/YE included epithelium development and regulation of

WNT signaling. According to the cell identity of EXMCs, mesenchyme development-associated terms were enriched in this cell lineage. Interestingly, we observed inflammatory response, regulation of immune system processes, and other GO terms enriched in the TE cells, suggesting their potential role in immune regulation during pregnancy [16] (Fig. 1F). Taken together, these findings indicate that combining embryo *in vitro* culture platform with powerful scATAC-seq can successfully generate comprehensive and high-quality maps of open chromatin and lineage regulators during early monkey embryogenesis.

Lineage specific transcriptional regulatory networks of monkey early embryonic development

To further characterize the transcriptional regulatory networks of monkey early embryonic development, we determined the lineage-specific TFs and their enriched motifs, as well as lineage-specific DPs. In addition, the gene activity scores and expression levels of the TF target genes were analyzed. As a result, a series of TFs, which may play important roles in cell lineage specification, were identified

(Supplementary Table S3). The gene expression levels, TF motif enrichment, and chromatin accessibility of the top ten lineage-specific TFs and their target genes were shown in Fig. 2A and 2B. Next, leveraging identified lineage-specific TFs and their target genes, we constructed modules of lineage-specific TFs and regulatory networks of target genes that were putatively co-regulated by two lineage-specific TF modules (EPI-VE/YE, EXMC-VE/YE, EXMC-TE, and TE-VE/YE). EPI and VE/YE lineages were highly related in the networks by hub TFs, such as FOXH1, indicating their similarities in the regulatory program during early embryogenesis. In contrast, TE and EXMC lineages were distinct from the other lineages (Fig. 2C). Also, the top five GO terms of TF target genes were shown in Fig. 2D. Consistent with the transcriptional regulatory network analysis, we observed that the genome regions of TF co-regulated genes were more accessible in co-regulated lineages. Notably, the expression of TF co-regulated genes displayed a lineage-specific pattern, suggesting that besides chromatin accessibility, additional mechanisms exist to guarantee lineage specification during early embryogenesis. For example, the expression and chromatin accessibility levels of FOXH1 target genes in the EPI and VE/YE (Fig. 2E), as these

genes are both involved in the WNT signaling pathway in the EPI and VE/YE (Fig. 2F).

Single cell chromatin accessibility reveals regulatory mechanisms of EPI lineage

To explore the regulatory mechanisms underpinnings the specification of EPI cells, Seurat [17] and FigR [18] we first applied to integrate the scRNA-seq and scATAC-seq datasets. We observed that most of the cells in the scRNA-seq and scATAC-seq datasets overlapped with each other, suggesting that the chromatin accessibility and gene expression in most EPI cells occur in a concordant manner during early embryonic development (Fig. 3A). The coembedded UMAP plot identified four cell clusters, and they were designated as EPI-A, EPI-B, EPI-C, and gastrulating cell (Gast)) (Fig. 3A) based on our previous study [5]. Next, the Monocle2 analysis [19] was applied to construct the developmental trajectory of EPI-A, -B, -C, and Gast cells. We observed that all the cells were ordered in a U-like trajectory, with EPI-A cells occupying one end and Gast cells occupying the other. EPI-B and -C cells were in the middle (Fig. 3B). Worth noting that identical

developmental trajectories were obtained when different algorithms were applied, such as Slingshot [20] and TSCAN [21] (Supplementary Fig. S2A). This trajectory revealed the continuous differentiation of EPI-A to Gast cells, as EPI-A cells are equivalent to ICM cells, EPI-B cells are equivalent to EPI cells at the pre-implantation stage (EPI-A and B were subsequently renamed as early EPI, early), EPI-C cells are equivalent to EPI cells at the early post-implantation stage. Gast cells are equivalent to *in vivo* gastrulation cells (Supplementary Fig. S2B).

To delineate the regulatory role of chromatin accessibility in EPI specification, we examined the cell type specific DPs from EPI-A to Gast cells. During the transition from EPI-A to B and EPI-C to Gast, most genome regions tended to gain accessibility. In contrast, they lost accessibility during the transition from EPI-B to C (Supplementary Fig. S2C and Table S4). Notably, Hox gene activation was observed when the EPI cells underwent gastrulation, indicating their important roles in primitive streak formation (Supplementary Fig. S2D). Interestingly, we observed that the activation of chromatin states preceded the expression of genes in some genome regions (Supplementary Fig. S2E), which implies an inconsistency between gene

expression and chromatin accessibility during EPI cell specification. The GO terms and TF binding motif enrichment analyses of DPs indicated that OTC4 and SOX2 might be involved in the transition from EPI-A to B and early post-implantation stage EPI (EPI-C) to gastrulating cells (Gast) (Supplementary Fig. S2E and 2F).

To interrogate the correlation between gene expression and chromatin accessibility, we related the DPs to the DEGs, and two main patterns were detected:1) chromatin became accessible first in EPI-A cells, and then the genes were expressed (pattern 1) (Fig. 3C); 2) the scaled values of gene expression and chromatin accessibility levels were comparable in EPI-A cells (Fig. 3D) (pattern 2). In pattern 1, four sub-patterns (clusters 1-4) were identified, and the GO enrichment analysis was performed. For example, pluripotency-related TFs, such as POU5F1, SOX2, and NANOG, belong to cluster 2, in which chromatin opens at pre- and peri-implantation stages (EPI-A and EPI-B, early-stage EPI) and gene expressions were up-regulated until the post-implantation stage (EPI-C) (Fig. 3C), which was also observed during the establishment of mouse PSCs in different pluripotent states [22].

In pattern 2, five sub-patterns (clusters 1-5) were observed. Interestingly, genes

involved in mesoderm formation and gastrulation belonged to pattern 2 (Fig. 3D). To further determine the dynamics of gene expression and chromatin accessibility during EPI specification, Monocle2-based pseudotime ordering of aligned single scRNA-seq and scATAC-seq cells was performed, and gene expression and chromatin accessibility levels were investigated. Similarly, two main patterns were observed:1) the opening of chromatin regions preceded the gene expression; 2) the opening of chromatin regions occurred concurrently with the gene expression, despite sub-patterns being detected in these two main patterns (Supplementary Fig. S3A).

As little is known about the pluripotency regulation of post-implantation EPI cells, we next focused our analysis on EPI-C and Gast cells. The EPI-C and Gast specific TFs (Supplementary Table S5), the chromatin accessibility, and the expression levels of TF target genes were determined. We observed that chromatin became accessible preceding the gene expression, indicative of an initial priming process of post-implantation EPI cells before their commitment to the specific lineage (Fig. 3E). Monocle2-based pseudotime ordering analysis of aligned single scRNA-seq and scATAC-seq cells further confirmed this observation (Supplementary Fig. S3B). We

then investigated mechanisms leading to the pluripotency transition from early EPI to Gast. We observed that the expressions of core pluripotent factors, such as OCT4 and NANOG, were upregulated in EPI-C cells, which highly correlated with the expression of FGF signaling members such as FGF2, FGF4 and FGF receptor 1 (FGFR1) (Fig. 3F). Furthermore, the expressions of gastrulation marker genes, including TBX3 and CDH2, was highly correlated with the expression of BMP and WNT signaling members. Neither FGF nor BMP signaling could regulate the expressions of naïve pluripotency related genes [23] (Fig. 3F), and the activation of chromatin regions corresponding to FGF and BMP signaling members preceded their expression (Fig. 3G). Thus, these results suggest that multiple mechanisms, including chromatin accessibility, guarantee EPI lineage specification.

Transcriptional regulation of trophoblast specification

The transcriptional mechanism underlying early trophoblast specification in primates is elusive, we then explored chromatin accessibility and gene expression profiles of trophoblasts. Louvain clustering analysis identified four distinct clusters (TE-A, TE-B,

TE-C, and TE-D) in the trophoblasts. Based on scRNA-seq analysis and alignment of single scRNA-seq and scATAC-seq cells (Supplementary Fig. S4A), we annotated these four identical types of trophoblasts in scATAC-seq profiles (Fig. 4A). After considering the gene expression levels, chromatin accessibility of marker genes and developmental trajectories (Fig. 4B and Supplementary Fig. S4B and S4C), TE-A cells were defined as TE cells, as they expressed the TE marker gene CDX2 [24, 25]; TE-B cells were defined as early stage CTs, as they expressed ITGA6 [26], and TE-C were defined as the proliferative CTs, as they expressed the mature CT marker KRT7 (CK7) [26] and the CT markers GATA3 [26] (Fig. 4B). Finally, TE-D were defined as EVTs, as they expressed the EVT markers, ITGA5 [26] and FN1 [27, 28] (Fig. 4B). To define the critical TFs involved in trophoblast lineage specification, we investigated the gene expression and chromatin accessibility of lineage specific TFs and their target genes (Supplementary Fig. S4C and Supplementary Table S6). In addition, the TF regulatory networks were generated, and key TFs involved in trophoblast lineage specification were identified: MSX2 and CDX2 in TE-A, zinc

finger containing proteins (ZNFs) including ZNF707, 75A and et al. in TE-B, EGR1 and EGR2 in TE-C, and EST2 and FOSL2 in TE-D were identified (Fig. 4C).

As we observed that the opening of chromatin regions preceded the gene expression alongside pseudotime ordering of single trophoblast cells during specification (Supplementary Fig. S4D), we sought to define the gene-regulatory mechanisms underlying trophoblast lineage specification by determining the gain and the loss of accessible peaks during transitions from TE-A to B, TE-B to C and TE-B to D (Supplementary Table S7). We observed that TE-B mainly gained open chromatin peaks during differentiation from TE-A. In contrast, TE-C lost open chromatin peaks during differentiation from TE-B (Fig. 4D), and corresponding genes were ranked according to their changed peaks (Supplementary Fig. S5A). The GO term enrichment analysis of gained and lost peaks was performed, and TF motif enrichment was calculated by chromVAR [29] (Supplementary Fig. S5B and S5C). These findings imply that chromatin is primed for lineage specification in trophoblast progenitor cells and gradually closed, accompanied by the further differentiation of progenitor to mature cell types.

A previous study reported that domains of regulatory chromatin (DORCs) are enriched in lineage-determining genes and can be used to infer cell fate choices *de novo* [30]. To delineate the cis-regulatory programs during trophoblast specification, we defined 941 DORC genes during trophoblast specification based on previously reported criteria (regions with >10 significant peak-gene associations) [30]. Consistent with the study in mouse skin cells, DORCs were enriched for critical regulators of trophoblast lineage specification, such as ASCL2 (regulator of human EVT differentiation) [31] and GCM1 (essential for the differentiation of human trophoblast cells along both villous and extra-villous pathways) [32] (Fig. 4E). Next, DORCs that were activated during lineage transition were identified (Fig. 4F). As DORCs activation precedes gene expressions [30], we analyzed chromatin accessibility and gene expressions of DORC genes. We also observed the chromatin activation of DORCs preceding gene expression during the differentiation from early CTs to mature CTs (TE-B to TE-C) and early CTs to EVTs (TE-B to TE-D) (Fig. 4G). These results indicate that chromatin activation is important for priming DORC loci before the final expression of DORC genes and commitment to specific cell lineages.

However, during specification from TEs (TE-A) to early stage CTs (TE-B), the activation of DORC regions and gene expressions were cogredient (Fig. 4G), indicating that another mechanism is involved in progenitor cell fate determination. Finally, the GO term enrichment analysis of DORC genes during lineage specification was performed, and the top ten GO terms are shown (Fig. 4H).

Finally, scRNA-seq based cross-species comparisons between mouse (embryonic day 6.5-8.5) [33] and monkey (9 d.p.f.-20 d.p.f.) gastrulation were performed as comparable mouse scATAC-seq dataset during this stage is not available. UMAP analysis showed that monkey EPI cells overlapped with mouse EPI and primitive streak cells, monkey VE/YE cells overlapped with mouse extraembryonic and visceral endoderm cells, and monkey TE cells overlapped with mouse extraembryonic ectoderm cells. Interestingly, monkey EXMCs clustered with mouse mesenchyme, suggesting their mesenchymal cell characteristics (Supplementary Fig. S6A). Then, the DEGs were determined between monkey and mouse identical cell types, and representative genes were shown in Supplementary Fig. S6B.

Lineage segregation between the epiblast and trophoblast

Previous studies indicated lineage flexibility between naïve PSCs and TEs [34, 35], however, the underlying mechanism remains elusive, we then sought to decipher the regulatory events during EPI and trophoblast lineage specification. First, the correlation of gene expression profiles between EPI and trophoblast cells in this study and the previously published dataset [5, 36] were calculated. We found that early stage EPI cells (ICM [36] and EPI-A) highly correlated with early stage TE cells (TE-A and pre-implantation early TE [36]) (Fig. 5A and Supplementary Fig. S7A). Moreover, the chromatin accessibility profiles also displayed a high correlation coefficient between early-stage EPI (EPI-A) and TE (TE-A) cells (Supplementary Fig. S7B). These data suggest a high similarity between early-stage EPI and TE at the transcriptional regulatory level.

To identify the key gene set involved in EPI and TE lineage specification, we explored a set of genes with expression differences gradually increasing between EPI and TE cells from peri to post-implantation transition in this and previously published dataset, respectively [5, 36] (Fig. 5B). Next, we assessed the overlap between these

two sets of genes and 220 genes (designated as EPI-trophoblast lineage driving genes, E-T driving genes) were identified (Fig. 5C, Supplementary Table S8). To weigh the importance of the 220 genes in EPI and trophoblast lineage segregation, the absolute value of the log fold change of the averaged 220 E-T driving genes between the EPI and trophoblast were calculated at peri and post-implantation stage, respectively (E-T expression difference). The same method was used to calculate the gene activity scores (E-T gene activity difference). Finally, log fold changes of the E-T expression differences and the E-T activity differences of the 220 genes were calculated. GNAO1 in the EPI and INSL4 in the trophoblast were identified (Fig. 5D). GO term enrichment analysis showed that genes differentially expressed in the EPI were enriched in regulating neuron differentiation, which was also observed in early post-implantation EPI cells *in vivo* [36]. Meanwhile, genes specifically expressed in trophoblast cells were enriched in placenta development and other terms (Fig. 5E). To identify TFs that are critical for EPI and trophoblast lineage segregation, TF motif enrichment analysis for the peaks linked to the 220 genes was conducted, and the expression of TFs was also detected (Supplementary Fig. S7C). Representative TFs

that potentially regulate EPI and trophoblast lineage identity are shown in Fig. 5F. To evaluate the weight of TFs in deriving the lineage specification of the EPI and trophoblast, we devised an approach to calculate the driving potential of TFs, which putatively bound to the 220 E-T driving genes (see Methods). We observed clear ordering of ZFNs and PATZ1 on the top of the list of TFs that putatively specify the EPI lineage and NR2F2 on the top of the list of TFs that are involved in trophoblast lineage determination (Fig. 5G), a role of that has been studied in humans [37]. Thus, the driving potential allows us to identify important TFs that play important roles in lineage specification.

Discussion

After implantation, mammalian embryos undergo dramatic lineage diversification and determination, and a multi-faceted regulatory process is involved to guarantee and achieve this cellular and molecular transition [38-40]. However, this multi-faceted regulatory process, especially the epigenetic mechanism, remains unsolved in primates. Using the scATAC-seq approach, we delineated the chromatin accessibility

landscape of *in vitro* cultured monkey embryos. Despite these advances, a low-throughput manual method was used here, and some cell lineages may have been entirely missed. In the future, high-throughput and spatial omics sequencing methods will provide more information on primate early post-implantation development.

In integrative scRNA-seq and scATAC-seq analysis, we observed chromatin opening before gene expression during EPI specification, suggesting EPI cells are permissive after implantation and ready for rapid differentiation. Lineage priming was also observed during the early differentiation of CTs to mature CTs or EVTs, but not during the specification of TE cells to early CTs. This observation is inconsistent with human hematopoietic stem cells and mouse skin cells [30, 41], implying that in addition to chromatin accessibility, another mechanism may underlie progenitor specification in monkey trophoblasts. Thus, we speculate that chromatin is primed before cell fate determination in cells requiring rapid specification.

Naïve PSCs have been reported to possess trophoblast differentiation capability [34, 35, 42-45]. Is this a conserved cellular mechanism in PSCs, or does it only exists in cultured PSCs, and which TFs networks and signaling pathways are involved in this

process? These questions remain unanswered. In this study, we identified similarities between early stage EPI and TE cells regarding gene expression and chromatin accessibility. Furthermore, after leveraging the 'driving potential' calculation, we identified a group of TFs that may involve in the trophoblast differentiation of naïve PSCs. Among them, NR2F2 has been confirmed as a marker of trophectoderm maturation [37]. Further studies that combine lineage tracing and genome editing will help us to understand early cell identity and plasticity.

Taken together, our findings help us understand the transcriptional regulation of primates' early post-implantation development and provide a valuable resource for regenerative medicine.

Methods

Animals

Healthy cynomolgus monkeys of 5 to 12 years old were used in this study. Monkeys were usually housed in groups. During superovulation and oocyte collection, they were provisionally caged individually at 16–26℃ under 40% to 70% relative humidity

and a 08:00 to 20:00 light vs. dark photoperiod, fed of commercial pelleted food and water ad libitum. Vaginal bleeding was observed twice daily to detect the onset of menses-the beginning of bleeding was defined as the 1st day of menstruation.

Single cell collection

The monkey embryos were cultured as previously reported [5]. After washing with phosphate buffered saline (PBS) (MA0008, Meilunbio), the embryos were cut into several pieces with a 1-mL syringe and digested into single cells with 0.1% trypsin (25200-072, GIBCO) at 37℃ for 3-5 minutes. After neutralization with 2% FBS (04-002-1A, Biological Industries), the cells were washed with ice-cold PBS containing 0.1-1% BSA. Finally, the individual cells were picked into ice-cold lysis buffer on ice with a mouth pipette for single cell ATAC libraries construction as previously described [5].

Pre-processing of scRNA-seq data

For the pre-processing of raw sequencing data, we removed adapters and filtered out low-quality reads with an N rate > 0.2 using Cutadapt (v1.15, RRID:SCR_011841) [46]. Filtered reads were then aligned to the *Macaca fascicularis* genome (Macaca_fascicularis_5.0) using STAR (v2.5.3, RRID:SCR_004463) [47]. Rsem-Calculate-Expression (RSEM, RRID:SCR_013027) (v1.3.0) [48] was used to calculate the read counts, which were then quantified as transcripts per million mapped reads (TPM). Cells with mapped reads < 1 million and ≤ 2000 genes with TPM values > 1 were filtered out.

Cell clustering and UMAP projection

We selected the top 2,000 variable genes based on log-transformed TPM matrices using Seurat (v3.2.2, RRID:SCR_016341) [17]. Principal components analysis (PCA) was performed, and the first 30 principal components (PCs) were used to build an SNN graph using the "FindNeighbors" function in the R package Seurat. We used UMAP (RRID:SCR_018217) [49] to visualize the distance between the cells on a two-dimensional map and Harmony (v1.0, RRID:SCR_018809) [50] to remove the

batch effects between the embryos. Cell clustering was performed using the “FindClusters” function in Seurat and cell-type annotations based on known cell-type specific marker genes. We identified marker genes between the clusters using the “FindAllMarkers” function in Seurat ($p < 0.05$). The top 2,000 DEGs were selected to construct the trajectory model using Monocle2 (v2.18.0, RRID:SCR_016339) [19, 51].

Comparisons between EPI cells *in vitro* and *in vivo*

We extracted the overlapping genes in EPI cells between our *in vitro* and *in vivo* embryos [36]. Then R package Seurat was used to integrate our *in vitro* and *in vivo* datasets. The log-transformed count expression matrices of genes x cells were used to create the Seurat object, and then we used normalized values processed by the “NormalizeData” function according to the tutorial of Seurat. The top 2,000 variable genes were identified using the “FindVariableFeatures” function, and scale gene expression values were obtained using the “ScaleData” function. The anchors between our and *in vivo* data were found with the FindTransferAnchors function

(anchor.features = 2,000, reduction = “cca”, dims = 1:30). The “IntegrateData” function (dims = 1:30) was applied to our and *in vivo* datasets to get an integrated Seurat object. Then the integrated Seurat object was processed with “ScaleData”, “RunPCA” and “RunUMAP” function, and a UMAP graph was constructed to visualize the similarities between *in vitro* and *in vivo* embryos. Next, we averaged the gene expression levels of each cell cluster in both datasets and calculated the Pearson correlations between the cell clusters in both datasets. The Euclidean distance between the cell clusters was calculated, and unsupervised hierarchical clustering was performed to determine gene expression pattern similarities between the *in vitro* and *in vivo* cell clusters.

Pre-processing of scATAC-seq data

The raw sequencing data were filtered using Cutadapt (v1.16) [46], and then filtered fragments were aligned to the *Macaca fascicularis* genome (Macaca_fascicularis_5.0) using Bowtie2 (v2.2.5, RRID:SCR_016368) [52]. Fragments with an alignment quality of > Q30 were retained, and duplicate fragments were removed. We filtered

out cells whose usable fragments were<10,000 and promoter regions (500 bp around the transcriptional start site) with a ratio of fragments <10%. Sambamba (v0.6.6) [53] was used to aggregate the fragments of all cells. Reference peaks were constructed using MACS2 (v2.1.2, RRID:SCR_013291) [54]. Finally, we counted fragments in the reference peaks using the “getCounts” function in chromVAR (v1.4.0) [29].

scATAC-seq dataset analysis

Signac (v0.2.5, RRID:SCR_021158) [55] was used to analyze the processed scATAC-seq dataset. We used latent semantic indexing (LSI) and UMAP to reduce the dimensions and visualize for the scATAC-seq dataset (dims = 1:6). Gene activity scores were calculated using the “FeatureMatrix” function to count fragments in the two kb-upstream regions of genes and gene bodies. Cell clustering was performed using Seurat’s “FindNeighbors” and “FindClusters” functions. Differentially accessible regions were identified using the “FindMarkers” function in Seurat with parameters of min.pct = 0.2 and test.use = 'LR'. The per-cell motif activity score was computed by chromVAR (“RunChromVAR” function in Signac). Finally, TF motif

enrichment of differentially accessible regions was performed using the "FindMotifs" function.

Integrated analysis of scRNA-seq and scATAC-seq datasets

The gene activity value was obtained by calculating the fragments in the 2 kb-upstream region and gene body, which could be used to measure the gene accessibility and for correlation analysis with the gene expressions. scATAC-seq and scRNA-seq pairs were matched by Seurat's canonical correlation analysis (CCA) using the "FindTransferAnchors" function (dims = 1:30, reduction = "cca") based on the top 2000 variable genes identified by "FindVariableFeatures" function, then scRNA-seq cell-type annotations information was transferred to scATAC-seq using "TransferData" function. To generate co-embedding UMAP, "FindIntegrationAnchors" (anchor.features = 2000, dims = 1:30) and "IntegrateData" (dims = 1:30) functions in Seurat were used. As previously described [56], we identified peak-to-gene links based on the null *trans* correlations. Differential peak-to-gene linkages were visualized by ComplexHeatmap (v2.7.8.1000, RRID:SCR_017270) [57]. Gene

Ontology (GO) analysis of the corresponding genes was performed using clusterProfiler (v3.18.1, RRID:SCR_016884) [58].

For constructing the transcription factor (TF)-target gene network, we first identified the TFs and marker genes highly expressed in the same group of cells. Next, we identified TF-target gene pairs, that is, if the marker gene was linked with peaks that matched the corresponding TF motif. For a given marker gene with at least one linked and matched peak, we summed their squared correlation R^2 as the linkage score for the TF-target pair. NetworkD3 (v0.4) package [59] in R V4.1.0 was used to visualize the TF- target gene network. We reconstructed the TF regulatory network using the dataset from the corresponding lineage for subtype analysis in each lineage.

Establishment of ATAC-RNA cell pairs and development trajectory analysis

ATAC and RNA cells were paired by the “pairCells” function in R package FigR [55] based on co-embedding PCA components, and pairs with ATAC and RNA cells in the same cell type were retained.

Since ATAC-RNA cell pairs were established, we could get “pseudo cells” in which each “pseudo cell” had chromatin accessibility and gene expression information like multi-modal data. Then paired RNA cells were used to create pseudotime trajectory using R package monocle2.

For the trajectory of EPI, we identified pseudotime-dependent genes whose expression varied with pseudotime using the “differentialGeneTest” function (fullModelFormulaStr = “~sm.ns(Pseudotime)”) and genes with *Q*-values < 0.1 were retained. Pseudotime-dependent genes were split into 4 main clusters based on expression and further split into some sub-clusters in each main cluster based on chromatin accessibility using the “plot_pseudotime_heatmap” function. ATAC and RNA pseudotime heatmap were combined using the “Heatmap” function in the R package ComplexHeatmap. Other pseudotime analysis methods, such as Slingshot (RRID:SCR_017012) [20] and TSCAN [21] were also used to create EPI developmental trajectory.

For the trajectory of TE, we identified branch-dependent genes whose expression varied with the branch using the “BEAM” function, and genes with *Q*-values < 0.001

were retained. Branch-dependent genes were split into 4 main clusters based on expression and further split into some sub-clusters in each main cluster based on chromatin accessibility using the “plot_genes_branched_heatmap” function. ATAC and RNA branched pseudotime heatmap were combined using the “Heatmap” function in the R package ComplexHeatmap.

Comparison of scRNA-seq datasets among mouse and monkey embryos

To get co-embedding UMAP for mouse and monkey embryos, we extracted cells in embryonic day 6.5-8 mouse embryos. 17,358 common genes were retained in mouse and monkey embryo data based on a homologous gene list from Ensembl BioMart. Then Seurat objects of mouse and monkey embryos were created, and the Seurat object of mouse embryos was split into 3 objects based on sequencing batch. Four objects were normalized using the “NormalizeData” function, and their variable genes were identified using the “FindVariableFeatures” function. The integration features between four objects were obtained using the “SelectIntegrationFeatures” function, which were inputted into the “ScaleData” and “RunPCA” functions to perform

principal component analysis (PCA). Then, we identified “anchors” between four objects by the “FindIntegrationAnchors” function (reduction = “rpca”, k.anchor = 20) and integrated four objects using the “IntegrateData” function. Co-embedding UMAP was generated by integrated object using the “RunUMAP” function.

Aiming to study the differences between monkey and mouse embryos in gene expressions, we matched cell types of cross-species in the shared neighborhood based on co-embedding UMAP and got mouse EPI, VE/YE, EXMC, and TE corresponding cell types in monkey embryos. Conserved cell type markers were identified using “FindConservedMarkers” functions with max *P*-values < 0.05 and min log2 fold changes > 0.25. DEGs were identified using “FindMarkers” functions in corresponding cell types between species with *P*-values < 0.05 and log2 fold changes > 0.25. And then, shared genes in DEGs with cell type markers were retained.

Analysis of gained and lost peaks

We identified the accessible peaks (peak read count was > 0) in each stage (the percentage of cells with accessible peaks was > 0.25). The gained peaks at a particular

stage were defined as the accessible peaks nonoverlapping with a previous stage. The lost peaks at a specific stage were defined as the peaks non-existing with this stage compared to a previous stage. Gained and lost peak-to-gene linkages were visualized by ComplexHeatmap (RRID:SCR_017270) [56]. GO analysis of the corresponding genes was performed using clusterProfiler (RRID:SCR_016884) [58].

Identification of genes involved in EPI and TE lineage specification

To study the genes with increasing expression differences during lineage differentiation, we identified the DEGs in EPI and TE sub-types using the "FindMarkers" function in Seurat and selected genes with increasing log fold change -log *P* values. EPI and TE up-regulated genes were classified based on the log fold change values.

Identification of TF regulating lineage specification

TF motif enrichment analysis was performed for genes upregulated in the EPI and TE. TF motif enrichment in the peaks of EPI and TE genes, *P* values less than 0.01 in EPI

and greater than 0.01 in TE, and the enrichment fold changes in EPI larger than that in TE were identified as EPI-regulated TFs, and vice versa.

To examine the importance of TFs in the corresponding group regulatory network, we calculated the degree centrality, closeness centrality, and eigenvector centrality of each TF in the network and the rank, respectively. The comprehensive rank was obtained by adding the ranks of the three centralities, and the higher the rank is, the more influential the TFs in the network.

Data Availability

All sequencing data were deposited at the National Center for Biotechnology Information Sequence Read Archive under accession no. SRP175059. The data were also deposited at the China National GeneBank (CNGB) Nucleotide Sequence Archive under accession no. CNP0000231. Mouse embryos' scRNA-seq dataset was downloaded from EMBL-EBI ArrayExpress under accession no. E-MTAB-6967. All supporting data and materials are available in the *GigaScience* GigaDB database [60].

Additional Files

Supplementary Table S1. Quality control data for scATAC-seq dataset.

Supplementary Table S2. Lineage-specific marker peaks and corresponding genes.

Supplementary Table S3. Lineage-specific TFs and their candidate target genes.

Supplementary Table S4. Gained or lost peaks and corresponding genes during EPI lineage transition.

Supplementary Table S5. EPI subtype lineage-specific TFs and corresponding target genes.

Supplementary Table S6. Trophoblast subtype lineage-specific TFs and corresponding target genes.

Supplementary Table S7. Gained or lost peaks and corresponding genes during trophoblast lineage transition.

Supplementary Table S8. Gene list of 220 genes involved in EPI and trophoblast lineage segregation.

Supplementary Fig. S1. Quality control of scATAC-seq data. (A) Bar charts showing the distribution of embryonic day in each cell type. Left panel, scATAC-seq dataset; right panel, scRNA-seq dataset. (B) Violin chart showing the quality control of the scATAC-seq dataset for each embryo. (C) Co-embedded UMAP for single cell pairs of scATAC-seq and scRNA-seq datasets based on geodesic distance-based pairing approach. The colors of cells represent technology and cell type. (D) The distribution of prediction scores calculated in the integrated procedure. (E) Percentage of each cell type in scATAC-seq and scRNA-seq datasets. (F) The chromatin accessibility and gene expression levels of cell type markers in the individual cell. Each gray line indicates one ATAC-RNA single cell pair. (G) UMAP plot of gene expression levels

and motif deviation scores of lineage-specific TFs.

Supplementary Fig. S2. Lineage specification of EPI. (A) Pseudotime trajectories of EPI cells inferred by slingshot (left) and TSCAN (right) analysis. (B) Hierarchical cluster analysis of expression profiles of EPI subtypes between *in vivo* [36] and *in vitro* embryos. (C) Bar chart showing the number of genes that gained or lost peaks during EPI subtype transitions. Genes are grouped by the number of peaks changed. (D) The number of accessible peaks changed for each gene during EPI subtype transitions. (E) Heatmap showing gained peaks and corresponding genes during EPI subtypes transitions with listed well-studied marker genes, TF binding motifs, candidate TFs, and enriched GO terms. *P* values derived from the hypergeometric test are shown, and the color indicates the gene ratios. (F) Heatmap showing lost peaks and corresponding genes during EPI subtypes transitions with listed well-studied marker genes, TF binding motifs, candidate TFs, and enriched GO terms. *P* values derived from the hypergeometric test are shown, and the color indicates the gene ratio.

Supplementary Fig. S3. The correlation between gene expression and chromatin accessibility during EPI specification. (A) Heatmaps showing gene activity scores and expression levels of pseudotime-dependent genes in Fig. 3B. Columns of the heatmap indicate pseudotime (left panel). Representative genes and GO enrichment terms of genes are listed on the middle and right panels. *P* values derived from the hypergeometric test are shown, and the color indicates the gene ratio. (B) Pseudotime heatmaps showing gene activity scores and expression levels of EPI-C and gastrulating cell (Gast) specific TF target genes in Fig. 3E.

Supplementary Fig. S4. Dynamics of trophoblast cell fate transitions. (A) Co-embedded UMAP for single cell pairs of scATAC-seq and scRNA-seq datasets based on geodesic distance-based pairing approach. The colors of cells represent technology and cell type. (B) Pseudotime trajectory of the scATAC-scRNA paired EPI subtypes with cells colored by trophoblast subtypes and pseudotime. (C) Heatmaps showing z-scores of trophoblast averaged subtype-specific TFs, their target gene

expression levels, and gene activity scores. Averaged gene expression levels and activity scores are calculated from cells aggregated by trophoblast subtypes. (D) Heatmaps showing gene activity scores and expression levels of branch-dependent genes in (B). Columns of the heatmap indicate pseudotime (left panel). Representative genes and GO enrichment terms are listed on the middle and right panels. *P* values derived from the hypergeometric test are shown, and the color indicates the gene ratio.

Supplementary Fig. S5. Dynamics of chromatin accessibility during trophoblast specification. (A) The number of changed accessible peaks of each gene during the transition of trophoblast subtypes. The genes are ranked by changed accessible peaks. (B) Heatmap showing gained peaks and corresponding genes during each trophoblast subtypes transitions with listed well-studied marker genes, TF binding motifs, corresponding candidate TFs, and representative enriched GO terms. *P* values derived from the hypergeometric test are shown, and the color indicates the gene ratio. (C) Heatmap showing lost peaks and corresponding genes during each trophoblast

subtype's transitions with listed well-studied marker genes, TF binding motifs, corresponding candidate TFs, and representative enriched GO terms. *P* values derived from the hypergeometric test are shown, and the color indicates the gene ratio.

Supplementary Fig. S6. Cross-species comparison between mouse and monkey gastrulating embryos. (A) UMAP visualization of cells of in vivo mouse (embryonic day 6.5-8) and in vitro monkey (9-20 d.p.f.) embryos. Colors encode cell source and type. (B) Heatmap of the top 40 DEG expressions between monkey and mouse embryos. Each column indicates matched cell cluster between monkey and mouse embryos.

Supplementary Fig. S7. Transcriptional regulation of EPI and trophoblast lineage segregation. (A) Heatmap showing Pearson correlation coefficient of gene expression profiles between EPI and TE subtypes. The EPI and TE cells were classified into four groups based on development stage and hierarchical clustering (pre_EPI_TE, post_TE, post_EPI, and Gast). pre, pre-implantation; post, post-implantation; Gast, gastrulating

cells. (B) Heatmap showing Pearson correlation coefficient of chromatin accessibility profiles between EPI and TE subtypes. The EPI and TE cells were classified into four groups (pre_EPI_TE, post_TE, post_EPI, and Gast). pre, pre-implantation; post, post-implantation; Gast, gastrulating cells. (C) Bubble plot showing TF expression and corresponding motif enrichments that regulate lineage differentiation. *P* values are derived from the hypergeometric test.

Ethics Approval

The ethical committee of the LPBR approved all animal and experiment procedures (LPBR-2016-01), and the procedures were performed by following the guidelines of the Association for Assessment and Accreditation of Laboratory Animal Care International (AAALAC) for the ethical treatment of non-human primates.

Competing Interests

The authors declare no competing interests.

Funding

This work was supported by the National Natural Science Foundation of China (82192871), the Natural Science Foundation of Yunnan Province (202001BC070001 and 202102AA100053), and the China National GeneBank (CNGB).

Authors' contributions

H.S., N.S., C.L., L.W., Y.Y., X.W., and L.L. performed most of the experiments. X.D., B.C., and B.B. performed the bioinformatics analysis. X.D., J.W, H.Y., W.J., Z.S., and T.T. participated in discussions. X.D., J. W., Z.S., and T.T. analyzed the data and wrote the manuscript. T.T. and Z.S. conceived and supervised the study.

References

1. Rossant J and Tam PPL. Early human embryonic development: Blastocyst formation to gastrulation. Dev Cell. 2022;57 2:152-65. doi:10.1016/j.devcel.2021.12.022.
2. Zhai J, Xiao Z, Wang Y and Wang H. Human embryonic development: from peri-implantation to gastrulation. Trends Cell Biol. 2022;32 1:18-29. doi:10.1016/j.tcb.2021.07.008.
3. Xiang L, Yin Y, Zheng Y, Ma Y, Li Y, Zhao Z, et al. A developmental landscape of 3D-cultured human pre-gastrulation embryos. Nature. 2020;577 7791:537-42. doi:10.1038/s41586-019-1875-y.

4. Ma H, Zhai J, Wan H, Jiang X, Wang X, Wang L, et al. In vitro culture of cynomolgus monkey embryos beyond early gastrulation. Science. 2019;366 6467 doi:10.1126/science.aax7890.
5. Niu Y, Sun N, Li C, Lei Y, Huang Z, Wu J, et al. Dissecting primate early post-implantation development using long-term in vitro embryo culture. Science. 2019;366 6467 doi:10.1126/science.aaw5754.
6. Deglincerti A, Croft GF, Pietila LN, Zernicka-Goetz M, Siggia ED and Brivanlou AH. Self-organization of the in vitro attached human embryo. Nature. 2016;533 7602:251-4. doi:10.1038/nature17948.
7. Shahbazi MN, Jedrusik A, Vuoristo S, Recher G, Hupalowska A, Bolton V, et al. Self-organization of the human embryo in the absence of maternal tissues. Nat Cell Biol. 2016;18 6:700-8. doi:10.1038/ncb3347.
8. Xiang Y, Zhang Y, Xu Q, Zhou C, Liu B, Du Z, et al. Epigenomic analysis of gastrulation identifies a unique chromatin state for primed pluripotency. Nat Genet. 2020;52 1:95-105. doi:10.1038/s41588-019-0545-1.
9. Yang X, Hu B, Liao J, Qiao Y, Chen Y, Qian Y, et al. Distinct enhancer signatures in the mouse gastrula delineate progressive cell fate continuum during embryo development. Cell Res. 2019;29 11:911-26. doi:10.1038/s41422-019-0234-8.
10. Argelaguet R, Clark SJ, Mohammed H, Stapel LC, Krueger C, Kapourani CA, et al. Multi-omics profiling of mouse gastrulation at single-cell resolution. Nature. 2019;576 7787:487-91. doi:10.1038/s41586-019-1825-8.
11. Ke Y, Xu Y, Chen X, Feng S, Liu Z, Sun Y, et al. 3D Chromatin Structures of Mature Gametes and Structural Reprogramming during Mammalian Embryogenesis. Cell. 2017;170 2:367-81 e20. doi:10.1016/j.cell.2017.06.029.
12. Zheng H, Huang B, Zhang B, Xiang Y, Du Z, Xu Q, et al. Resetting Epigenetic Memory by Reprogramming of Histone Modifications in Mammals. Mol Cell. 2016;63 6:1066-79. doi:10.1016/j.molcel.2016.08.032.
13. Wang C, Liu X, Gao Y, Yang L, Li C, Liu W, et al. Reprogramming of H3K9me3-dependent heterochromatin during mammalian embryo development. Nat Cell Biol. 2018;20 5:620-31. doi:10.1038/s41556-018-0093-4.

14. Shahbazi MN and Zernicka-Goetz M. Deconstructing and reconstructing the mouse and human early embryo. Nat Cell Biol. 2018;20 8:878-87. doi:10.1038/s41556-018-0144-x.
15. Mole MA, Weberling A and Zernicka-Goetz M. Comparative analysis of human and mouse development: From zygote to pre-gastrulation. Curr Top Dev Biol. 2020;136:113-38. doi:10.1016/bs.ctdb.2019.10.002.
16. Yockey LJ and Iwasaki A. Interferons and Proinflammatory Cytokines in Pregnancy and Fetal Development. Immunity. 2018;49 3:397-412. doi:10.1016/j.immuni.2018.07.017.
17. Stuart T, Butler A, Hoffman P, Hafemeister C, Papalexi E, Mauck WM, 3rd, et al. Comprehensive Integration of Single-Cell Data. Cell. 2019;177 7:1888-902 e21. doi:10.1016/j.cell.2019.05.031.
18. Kartha VK, Duarte FM, Hu Y, Ma S, Chew JG, Lareau CA, et al. Functional inference of gene regulation using single-cell multi-omics. Cell Genom. 2022;2 9 doi:10.1016/j.xgen.2022.100166.
19. Qiu X, Hill A, Packer J, Lin D, Ma YA and Trapnell C. Single-cell mRNA quantification and differential analysis with Census. Nat Methods. 2017;14 3:309-15. doi:10.1038/nmeth.4150.
20. Street K, Risso D, Fletcher RB, Das D, Ngai J, Yosef N, et al. Slingshot: cell lineage and pseudotime inference for single-cell transcriptomics. BMC Genomics. 2018;19 1:477. doi:10.1186/s12864-018-4772-0.
21. Ji Z and Ji H. TSCAN: Pseudo-time reconstruction and evaluation in single-cell RNA-seq analysis. Nucleic Acids Res. 2016;44 13:e117. doi:10.1093/nar/gkw430.
22. Shen H, Yang M, Li S, Zhang J, Peng B, Wang C, et al. Mouse totipotent stem cells captured and maintained through spliceosomal repression. Cell. 2021;184 11:2843-59 e20. doi:10.1016/j.cell.2021.04.020.
23. Messmer T, von Meyenn F, Savino A, Santos F, Mohammed H, Lun ATL, et al. Transcriptional Heterogeneity in Naive and Primed Human Pluripotent Stem Cells at Single-Cell Resolution. Cell Rep. 2019;26 4:815-24 e4. doi:10.1016/j.celrep.2018.12.099.
24. Strumpf D, Mao CA, Yamanaka Y, Ralston A, Chawengsaksophak K, Beck F, et al. Cdx2 is required for correct cell fate specification and differentiation

of trophectoderm in the mouse blastocyst. Development. 2005;132 9:2093-102. doi:10.1242/dev.01801.

25. Sritanaudomchai H, Sparman M, Tachibana M, Clepper L, Woodward J, Gokhale S, et al. CDX2 in the formation of the trophectoderm lineage in primate embryos. Dev Biol. 2009;335 1:179-87. doi:10.1016/j.ydbio.2009.08.025.

26. Okae H, Toh H, Sato T, Hiura H, Takahashi S, Shirane K, et al. Derivation of Human Trophoblast Stem Cells. Cell Stem Cell. 2018;22 1:50-63 e6. doi:10.1016/j.stem.2017.11.004.

27. Dong C, Beltcheva M, Gontarz P, Zhang B, Popli P, Fischer LA, et al. Derivation of trophoblast stem cells from naive human pluripotent stem cells. Elife. 2020;9 doi:10.7554/eLife.52504.

28. Telugu BP, Adachi K, Schlitt JM, Ezashi T, Schust DJ, Roberts RM, et al. Comparison of extravillous trophoblast cells derived from human embryonic stem cells and from first trimester human placentas. Placenta. 2013;34 7:536-43. doi:10.1016/j.placenta.2013.03.016.

29. Schep AN, Wu B, Buenrostro JD and Greenleaf WJ. chromVAR: inferring transcription-factor-associated accessibility from single-cell epigenomic data. Nat Methods. 2017;14 10:975-8. doi:10.1038/nmeth.4401.

30. Ma S, Zhang B, LaFave LM, Earl AS, Chiang Z, Hu Y, et al. Chromatin Potential Identified by Shared Single-Cell Profiling of RNA and Chromatin. Cell. 2020;183 4:1103-16 e20. doi:10.1016/j.cell.2020.09.056.

31. Varberg KM, Iqbal K, Muto M, Simon ME, Scott RL, Kozai K, et al. ASCL2 reciprocally controls key trophoblast lineage decisions during hemochorial placenta development. Proc Natl Acad Sci U S A. 2021;118 10 doi:10.1073/pnas.2016517118.

32. Baczyk D, Drewlo S, Proctor L, Dunk C, Lye S and Kingdom J. Glial cell missing-1 transcription factor is required for the differentiation of the human trophoblast. Cell Death Differ. 2009;16 5:719-27. doi:10.1038/cdd.2009.1.

33. Pijuan-Sala B, Griffiths JA, Guibentif C, Hiscock TW, Jawaid W, Calero-Nieto FJ, et al. A single-cell molecular map of mouse gastrulation and early organogenesis. Nature. 2019;566 7745:490-5.

doi:10.1038/s41586-019-0933-9.

34. Guo G, Stirparo GG, Strawbridge SE, Spindlow D, Yang J, Clarke J, et al. Human naive epiblast cells possess unrestricted lineage potential. Cell Stem Cell. 2021;28 6:1040-56 e6. doi:10.1016/j.stem.2021.02.025.

35. Io S, Kabata M, Iemura Y, Semi K, Morone N, Minagawa A, et al. Capturing human trophoblast development with naive pluripotent stem cells in vitro. Cell Stem Cell. 2021;28 6:1023-39 e13. doi:10.1016/j.stem.2021.03.013.

36. Nakamura T, Okamoto I, Sasaki K, Yabuta Y, Iwatani C, Tsuchiya H, et al. A developmental coordinate of pluripotency among mice, monkeys and humans. Nature. 2016;537 7618:57-62. doi:10.1038/nature19096.

37. Meistermann D, Bruneau A, Loubersac S, Reignier A, Firmin J, Francois-Campion V, et al. Integrated pseudotime analysis of human pre-implantation embryo single-cell transcriptomes reveals the dynamics of lineage specification. Cell Stem Cell. 2021;28 9:1625-40 e6. doi:10.1016/j.stem.2021.04.027.

38. Peng G and Jing N. The genome-wide molecular regulation of mouse gastrulation embryo. Sci China Life Sci. 2017;60 4:363-9. doi:10.1007/s11427-016-0285-3.

39. Parfitt DE and Shen MM. From blastocyst to gastrula: gene regulatory networks of embryonic stem cells and early mouse embryogenesis. Philos Trans R Soc Lond B Biol Sci. 2014;369 1657 doi:10.1098/rstb.2013.0542.

40. Tam PP and Behringer RR. Mouse gastrulation: the formation of a mammalian body plan. Mech Dev. 1997;68 1-2:3-25. doi:10.1016/s0925-4773(97)00123-8.

41. Ranzoni AM, Tangherloni A, Berest I, Riva SG, Myers B, Strzelecka PM, et al. Integrative Single-Cell RNA-Seq and ATAC-Seq Analysis of Human Developmental Hematopoiesis. Cell Stem Cell. 2021;28 3:472-87 e7. doi:10.1016/j.stem.2020.11.015.

42. Yu L, Wei Y, Duan J, Schmitz DA, Sakurai M, Wang L, et al. Blastocyst-like structures generated from human pluripotent stem cells. Nature. 2021;591 7851:620-6. doi:10.1038/s41586-021-03356-y.

43. Fan Y, Min Z, Alsolami S, Ma Z, Zhang E, Chen W, et al. Generation of human blastocyst-like structures from pluripotent stem cells. Cell Discov. 2021;7

1:81. doi:10.1038/s41421-021-00316-8.
44. Kagawa H, Javali A, Khoei HH, Sommer TM, Sestini G, Novatchkova M, et al. Human blastoids model blastocyst development and implantation. Nature. 2022;601 7894:600-5. doi:10.1038/s41586-021-04267-8.
45. Liu X, Tan JP, Schroder J, Aberkane A, Ouyang JF, Mohenska M, et al. Modelling human blastocysts by reprogramming fibroblasts into iBlastoids. Nature. 2021;591 7851:627-32. doi:10.1038/s41586-021-03372-y.
46. Martin M. Cutadapt removes adapter sequences from high-throughput sequencing reads. EMBnet J 2011;17:10-2.
47. Dobin A, Davis CA, Schlesinger F, Drenkow J, Zaleski C, Jha S, et al. STAR: ultrafast universal RNA-seq aligner. Bioinformatics. 2013;29 1:15-21. doi:10.1093/bioinformatics/bts635.
48. Li B and Dewey CN. RSEM: accurate transcript quantification from RNA-Seq data with or without a reference genome. BMC Bioinformatics. 2011;12:323. doi:10.1186/1471-2105-12-323.
49. Leland McInnes JH, James Melville. Umap: Uniform manifold approximation and projection for dimension reduction. arXiv preprint arXiv. 2018;1802.
50. Korsunsky I, Millard N, Fan J, Slowikowski K, Zhang F, Wei K, et al. Fast, sensitive and accurate integration of single-cell data with Harmony. Nat Methods. 2019;16 12:1289-96. doi:10.1038/s41592-019-0619-0.
51. Qiu X, Mao Q, Tang Y, Wang L, Chawla R, Pliner HA, et al. Reversed graph embedding resolves complex single-cell trajectories. Nat Methods. 2017;14 10:979-82. doi:10.1038/nmeth.4402.
52. Langmead B and Salzberg SL. Fast gapped-read alignment with Bowtie 2. Nat Methods. 2012;9 4:357-9. doi:10.1038/nmeth.1923.
53. Tarasov A, Vilella AJ, Cuppen E, Nijman IJ and Prins P. Sambamba: fast processing of NGS alignment formats. Bioinformatics. 2015;31 12:2032-4. doi:10.1093/bioinformatics/btv098.
54. Zhang Y, Liu T, Meyer CA, Eeckhoute J, Johnson DS, Bernstein BE, et al. Model-based analysis of ChIP-Seq (MACS). Genome Biol. 2008;9 9:R137. doi:10.1186/gb-2008-9-9-r137.

55. Stuart T, Srivastava A, Madad S, Lareau CA and Satija R. Single-cell chromatin state analysis with Signac. Nat Methods. 2021;18 11:1333-41. doi:10.1038/s41592-021-01282-5.

56. Granja JM, Klemm S, McGinnis LM, Kathiria AS, Mezger A, Corces MR, et al. Single-cell multiomic analysis identifies regulatory programs in mixed-phenotype acute leukemia. Nat Biotechnol. 2019;37 12:1458-65. doi:10.1038/s41587-019-0332-7.

57. Gu Z, Eils R and Schlesner M. Complex heatmaps reveal patterns and correlations in multidimensional genomic data. Bioinformatics. 2016;32 18:2847-9. doi:10.1093/bioinformatics/btw313.

58. Yu G, Wang LG, Han Y and He QY. clusterProfiler: an R package for comparing biological themes among gene clusters. OMICS. 2012;16 5:284-7. doi:10.1089/omi.2011.0118.

59. J.J. A, C. G, K. R and C. Y. NetworkD3: D3 JavaScript Network Graphs from R. 2017.

60. Tan T, Dai X, Shao H, Sun N, Ci B, Wu J, et al. Supporting data for "Developmental dynamics of chromatin accessibility during post-implantation development of monkey embryos" GigaScience Database 2023; doi:10.5524/102384.

Figure Legends

Figure 1 Landscape of chromatin accessibility during monkey peri- and post-implantation development

(A) Schematic illustration of scATAC sequencing of monkey embryos at different developmental stages. (B) UMAP plot of all the scATAC-seq and scRNA-seq cells. Cells are colored by their cell-type annotation. ATAC cell types were transferred from

RNA. (C) Aggregated scATAC-seq tracks denoting the chromatin accessibility peaks for the marker genes of each cell type. Peak-to-gene linkages are shown at the bottom, and correlations are represented by arcs colored by the correlation score (color scales for both panels are to the right). (D) Heatmap showing DPs and corresponding DEGs for each cell type and some well-studied lineage markers are listed. Each row shows one DP with its corresponding gene, whereas some genes are duplicated in a row cluster as multiple peaks link to one gene. (E) Representative lineage-specific TFs and their binding motifs in DPs. (F) Representative enriched GO terms within each lineage. *P* values derived from the hypergeometric test are shown, and the color indicates the gene ratio.

Figure 2 TF regulatory networks of early monkey embryogenesis

(A) Z-scores of averaged lineage-specific TF expression and corresponding motif enrichment in the DPs of each lineage (left panel). Z-scores of averaged lineage-specific TF target genes expression levels and averaged gene activity scores calculated from cells aggregated by lineage (right panel). Only the top ten

lineage-specific TFs of each lineage with the most significant *P* values in the DEGs are shown here. (B) Heatmaps showing the averaged gene expression levels and activity scores of target genes of representative lineage-specific TFs. (C) Lineage-specific TF regulatory networks and target genes regulated by multiple lineage-specific TF modules are shown. The representative target genes are listed beside. (D) The corresponding genes' top five GO enrichment terms are listed in (C). (E) FOXH1 target gene activity scores and expression levels in the EPI and VE/YE lineages, respectively. All values are min-max normalized. (F) Representative GO enrichment terms of FOXH1 target genes. *P* values derived from the hypergeometric test are shown, and the color indicates the gene ratio.

Figure 3 Transcriptional regulation of EPI specification

(A) Co-embedded UMAP for single cell pairs of scATAC-seq and scRNA-seq datasets based on geodesic distance-based pairing approach. The colors of cells represent technology and cell type. (B) Pseudotime trajectory of the scATAC-scRNA paired EPI subtypes. Cells are colored by the EPI subtypes and pseudotime. (C)

Heatmaps showing gene activity scores and expression levels of subtype-specific DEGs in pattern 1, which are classified into four clusters based on hierarchical clustering. All values are min-max normalized (left panel). Line chart showing the averaged gene activity scores and gene expression levels in heatmap clusters and representative genes are listed (middle panel). Bar chart showing the representative GO enrichment terms of genes in heatmap clusters. *P* values derived from the hypergeometric test are shown, and the color indicates the gene ratio (right panel). (D) Heatmaps showing gene activity scores and expression levels of subtype-specific DEGs in pattern 2, which are classified into five clusters based on hierarchical clustering, and all values were min-max normalized (left panel). Line chart showing the averaged gene activity scores and gene expression levels in heatmap clusters and representative genes are listed (middle panel). Bar chart showing the representative GO enrichment terms of genes in heatmap clusters. *P* values derived from the hypergeometric test are shown, and the color indicates the gene ratio (right panel). (E) Box chart of averaged EPI-C and gastrulating cell (Gast) specific TF target gene activity scores and expression levels. (F) Heatmap showing the correlation

coefficients of gene expression levels among the representative genes. These genes were classified into three groups by hierarchical clustering. They were up-regulated in early EPI (EPI-A and EPI-B), EPI-C, and Gast, respectively. (G) Heatmaps showing gene activity scores and gene expression levels corresponding to (F) and all values were min-max normalized.

Figure 4 Chromatin accessibility dynamics of trophoblast specification during monkey early embryonic development

(A) UMAP plot of RNA-sequenced and ATAC-sequenced single cells derived from the trophoblast lineage, with cells colored by trophoblast subtypes. (B) Heatmaps showing averaged gene activity scores and expression levels of representative marker genes. The color bar represents the z-scores calculated from the trophoblast (TE) subtype aggregates. A gradient of blue, gray, and red indicates low to high values. (C) TF regulatory networks of each trophoblast subtype are shown. Gray dots indicate target genes, colored dots indicate trophoblast subtype-specific TFs, and lines indicate regulatory relationships between TFs and target genes. (D) Bar chart showing gene

numbers, with gains or losses peaks during trophoblast specification and 1, 2, and ≥3 indicating peak change numbers. (E) The number of significantly correlated peaks ($p < 0.05$) for each gene (±25 kb from transcription start sites (TSSs)). DORC genes situated above the dotted line are shown. (F) Changes in peak numbers and expression levels of genes during trophoblast specification from TE-A to TE-B (A2B), TE-B to TE-C (B2C), and TE-B to TE-D (B2D) are shown. The x-axis indicates the averaged log fold change between the later and earlier stages, and the y-axis indicates the corresponding number change of the accessible peak. (G) Accessible peak numbers and expression levels of upregulated DORC genes and all values were min-max normalized. (H) Bar chart showing representative GO terms enrichment in (G). *P* values derived from the hypergeometric test are shown, and the color indicates the gene ratio.

Figure 5 Transcriptional regulation of EPI and TE segregation

(A) Pearson correlation coefficient networks of gene expression profiles between EPI and trophoblast subtypes. Subtypes are classified into four groups based on

developmental stage and hierarchical clustering. Line width represents the coefficient correlation, and the lines with a coefficient correlation < 0.7 were removed. preE, pre-implantation early; preL, pre-implantation late; pre, pre-implantation; post, post-implantation; pa, parietal. (B) Averaged log fold change (top) and -log10 (*P* value) (bottom) of DEGs in the EPI and trophoblast from peri- to post-implantation of monkey embryos *in vivo* and *in vitro*. The x-axis indicates the ranked gene list, which was ordered by the averaged log fold change between EPI-A and TE-A, EPI-B and EPI-C, and TE-C and TE-D, respectively, in *in vitro* cultured embryos, and between ICM and preE-TE, pre-EPI and preL-TE, and post-implantation EPI (postE-EPI, postL-EPI, and Gast1) and post-implantation TE (post-paTE) in *in vivo* embryos [36]. preE, pre-implantation early; preL, pre-implantation late; pre, pre-implantation; post, post-implantation; pa, parietal. (C) Venn diagram showing genes involved in EPI and trophoblast segregation. The 220 overlapping genes are conserved between embryos *in vivo* and *in vitro*. (D) Weighting the importance of the 220 genes in epiblast and trophoblast segregation. Dot chart showing gene expression level difference ratios and chromatin accessibility difference ratios in the 220 genes in (C). The ratios of the

gene expression levels or chromatin accessibility differences were expressed as the post-implantation stage log fold change to the pre-implantation stage log fold change. The dot color indicates how many TFs potentially regulate a specific gene. (E) Representative enriched GO terms of 220 genes in the EPI and trophoblast. *P* values derived from the hypergeometric test are shown, and the color indicates the gene ratio. (F) Representative TFs that putatively regulate the segregation of the EPI and trophoblast. All expression levels are min-max normalized, and the motif enrichment is z-scores. (G) Weighting the ranking of the TFs that putatively regulate the expression of the 220 EPI and trophoblast lineage segregation genes.

Figure 1

Click here to access/download;Figure;fig1.jpg

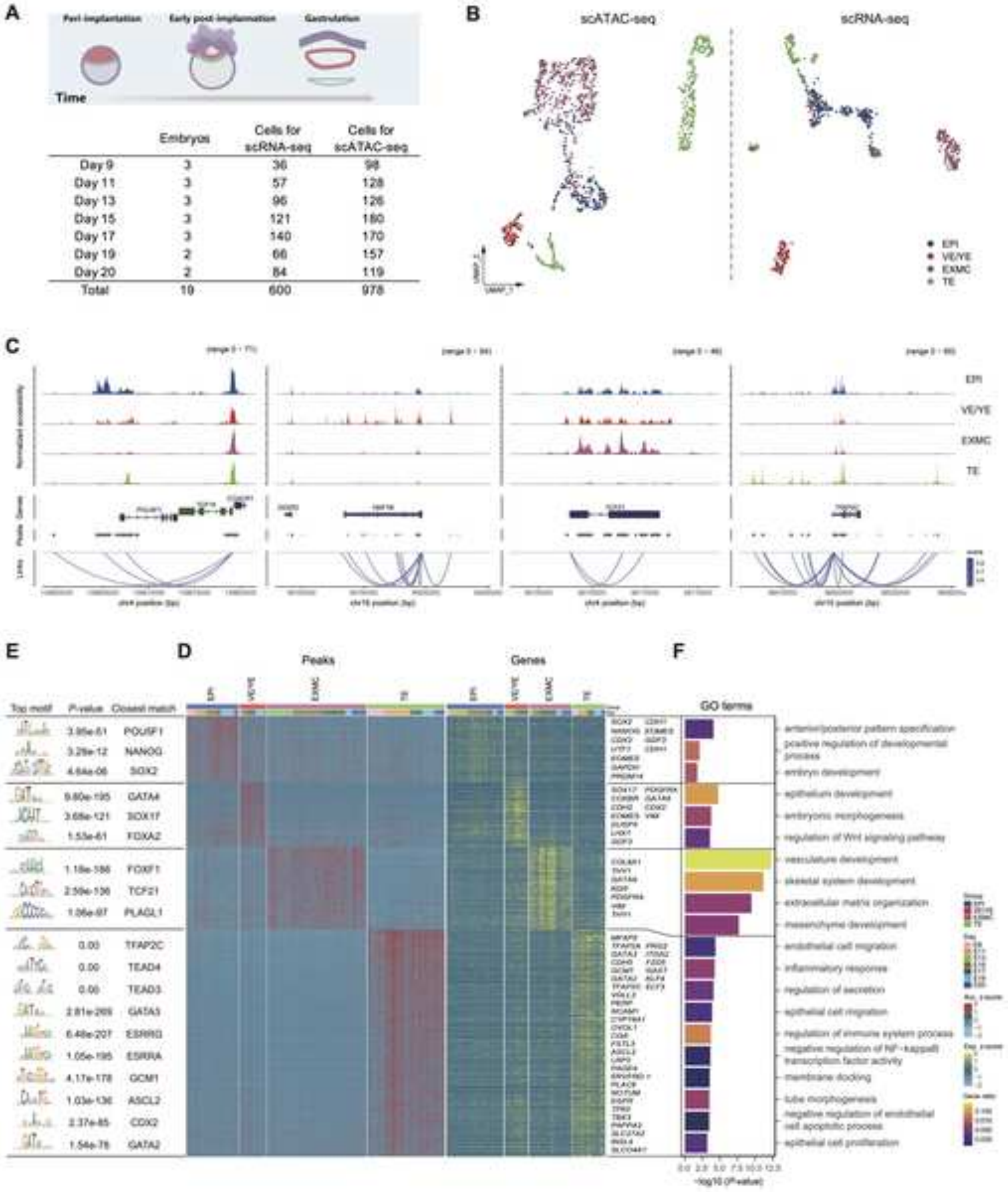

Figure 2

Click here to access/download;Figure;fig2.jpg

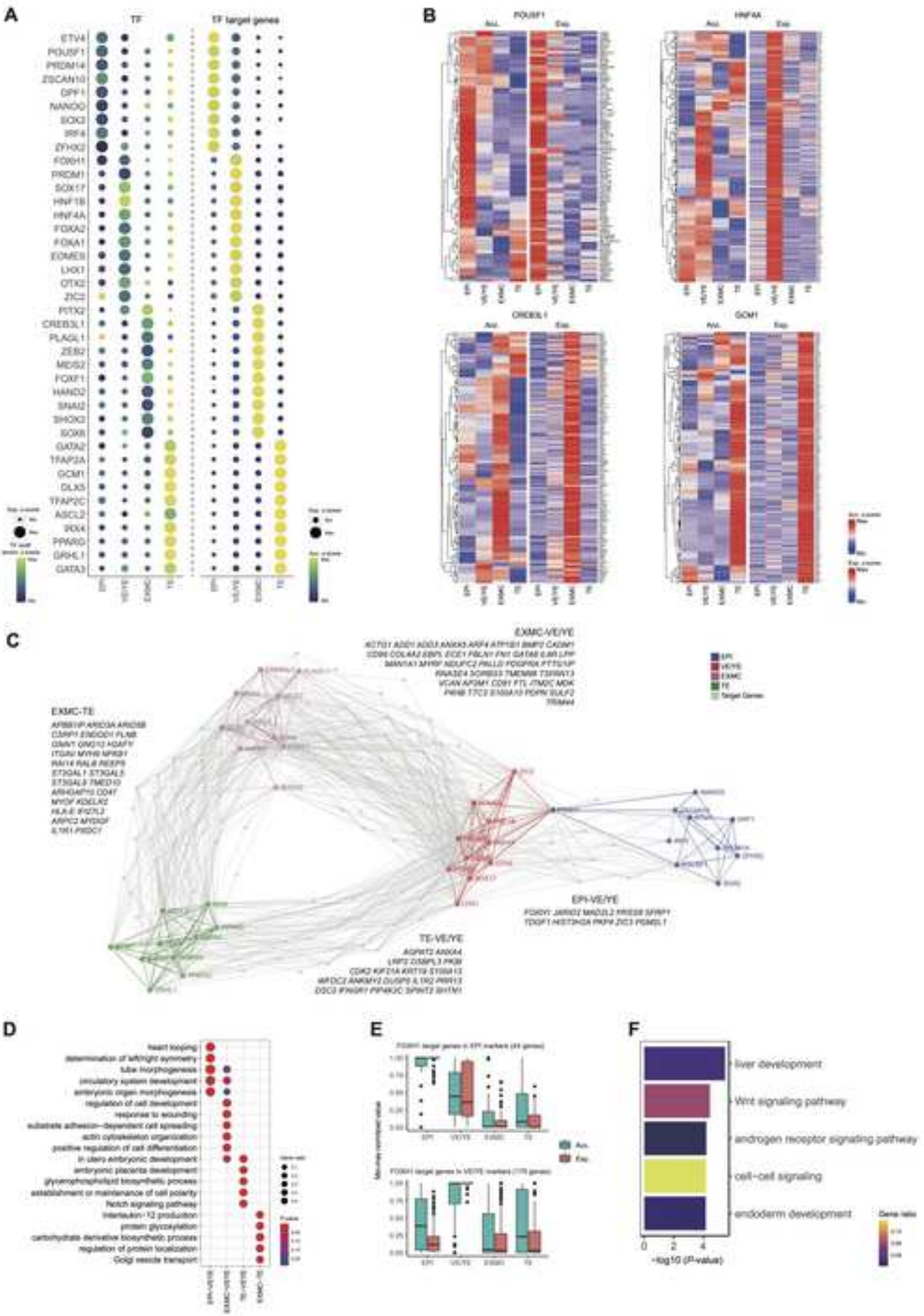

Figure 3 Click here to access/download;Figure;fig3.jpg

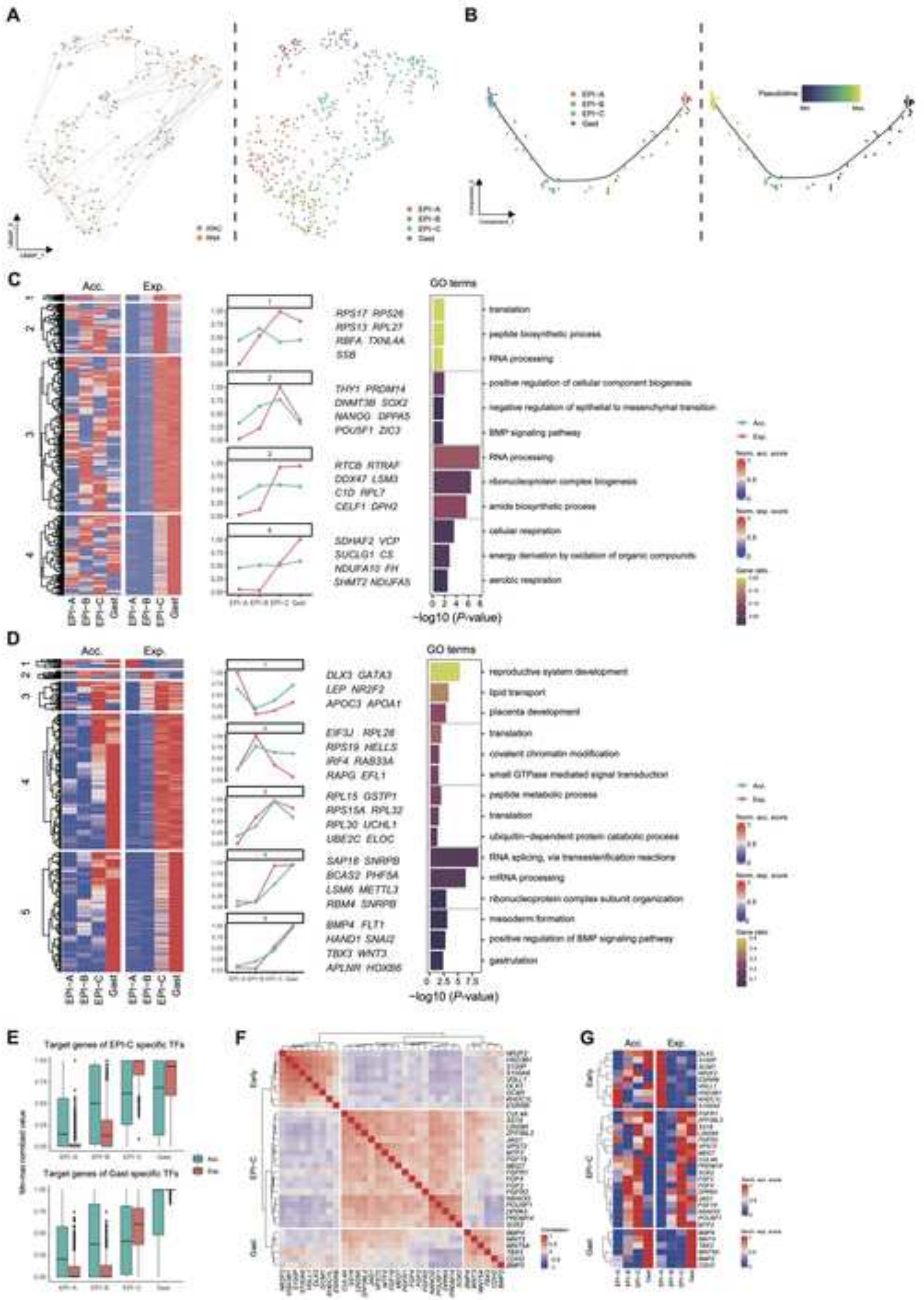

Figure 4

Click here to access/download;Figure;fig4.jpg

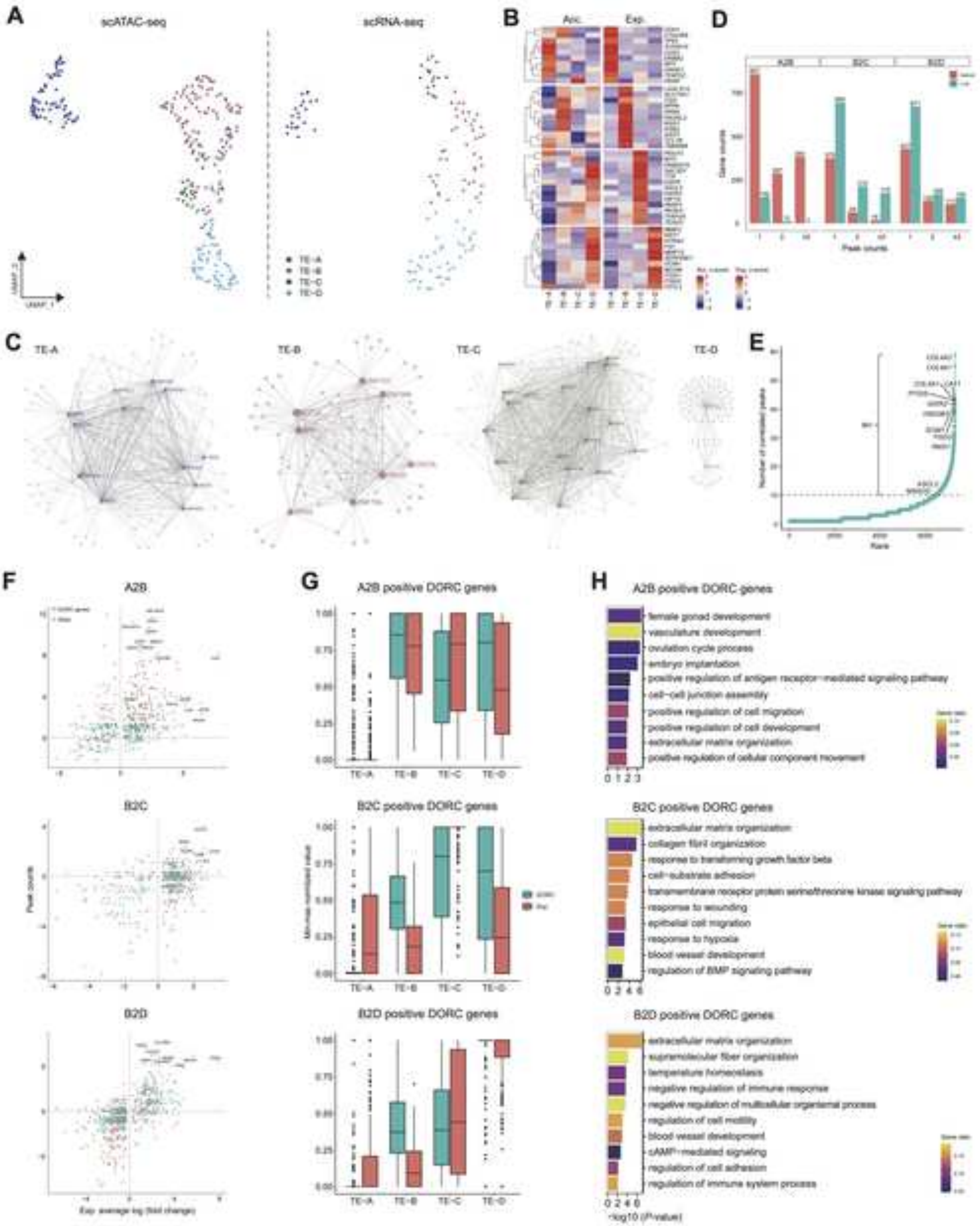

Figure 5

Click here to access/download;Figure;fig5.jpg

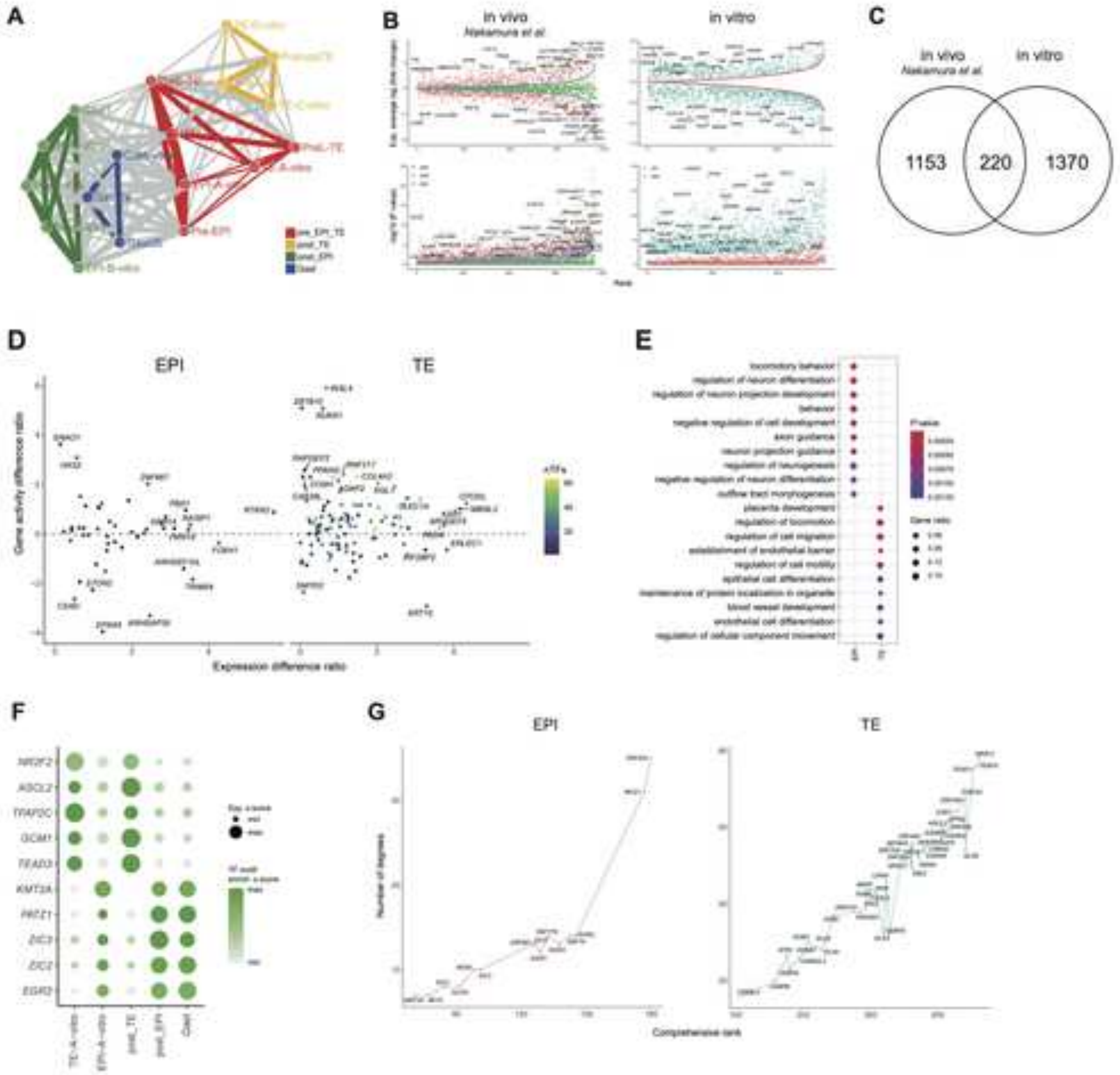

Supplementary Fig. 1

Click here to access/download
Supplementary Material
figS1.jpg

Supplementary Fig. 2

Click here to access/download
Supplementary Material
figS2.jpg

Supplementary Fig. 3

Click here to access/download
Supplementary Material
figS3.jpg

Supplementary Fig. 4

Click here to access/download
Supplementary Material
figS4.jpg

Supplementary Fig. 5

Click here to access/download
Supplementary Material
figS5.jpg

Supplementary Fig. 6

Click here to access/download
Supplementary Material
figS6.jpg

Supplementary Fig. 7

Click here to access/download
Supplementary Material
figS7.jpg

Supplementary Table S1

Click here to access/download
Supplementary Material
TableS1.xlsx

Supplementary Table S2

Click here to access/download

Supplementary Material

TableS2.xlsx

Supplementary Table S3

Click here to access/download
Supplementary Material
TableS3.xlsx

Supplementary Table S4

Click here to access/download
Supplementary Material
TableS4.xlsx

Supplementary Table S5

Click here to access/download
Supplementary Material
TableS5.xlsx

Supplementary Table S6

Click here to access/download
Supplementary Material
TableS6.xlsx

Supplementary Table S7

Click here to access/download
Supplementary Material
TableS7.xlsx

Supplementary Table S8

Click here to access/download
Supplementary Material
TableS8.xlsx

25 Mar 2023
Dear Dr. Hongling,

I am attaching our revised manuscript (GIGA-D-22-00278) titled "**Developmental dynamics of chromatin accessibility during post-implantation development of monkey embryos**". We thank you and the reviewers for the positive comments and suggestions, which helped improve our manuscript immensely. We have provided a detailed point-by-point response and revised the manuscript to address the remaining concerns.

If you have any questions, please do not hesitate to contact us.

Tao Tan
State Key Laboratory of Primate Biomedical Research
Institute of Primate Translational Medicine
Kunming University of Science and Technology

Our responses to the reviewers' comments are in highlighted blue.

Referee #1:

The authors present a scATAC-seq dataset of monkey embryo development. A major limitation of the analysis is that the authors seem to pool cells within each cell type instead directly doing analysis on a single-cell level, which largely loses the power and resolution of single-cell analysis. Below are my specific comments:

[Response] We greatly thank the reviewer for the constructive comments and agree that analysis on the single-cell level is necessary. We have added the single-cell level analysis in Supplementary Fig. S1C-1G, Fig. 3A-3B, Supplementary Fig. S2A, Supplementary Fig. S3, Supplementary Fig. S4A, S4B, and S4D based on the reviewer's suggestion.

Fig 1d: What is each row? Is each row showing one DP and its corresponding gene, or one DEG and its corresponding peak? Or are there duplicated genes or peaks in the plot?

[Response] We have added the descriptions in the figure legend of Fig 1D to clarify this ambiguity. Each row shows one DP with its corresponding gene, whereas some genes are duplicated in a row cluster as multiple peaks link to one gene. (Page 50, lines 880-882).

For the single-cell analysis, the authors claim that ' which implies that there is a time lag between gene expression and chromatin accessibility during EPI cell specification'. However, this may not be true biology since the scRNA and scATAC were computationally aligned. The time lag could be introduced by the computational alignment.

[Response] We thank the reviewer for raising this point. In our bioinformatic analysis approach, similar clusters were aligned between scRNA-seq and scATAC-seq datasets based on the top 2,000 variable genes, and high consistency between matched clusters was observed (also see Methods) (Page 29, lines 457-462), implying similar clusters of scRNA-seq and scATAC-seq datasets were well aligned (Fig. 3A). Based on this observation, the asynchrony features instead of the congruent features in the aligned clusters are plausible. To further clarify the possibility that the computational alignment could introduce the time lag, we have aligned single ATAC and RNA cells in the revised manuscript based on a geodesic distance-based pairing approach using “FigR” which can reduce the deviation caused by cell cluster mean value to a certain extent (Kartha et al., 2022) (Fig.3A). Notably, a time lag between gene expression and chromatin accessibility during EPI cell specification was also observed (Supplementary Fig. S3A). We also have toned down this claim as “which implies an inconsistency between gene expression and chromatin accessibility during EPI cell specification” in the revised manuscript (Page 12, lines 184-185).

For the pattern analysis in Figure 3C,D, why not use the pseudotime ordering and perform the analysis on a single-cell level, instead of the current analysis that pools cells with the same cell type

[Response] We have aligned the nearest scATAC and scRNA cells by the “pairCells” function in the R package FigR (Kartha *et al.*, 2022) based on co-embedding PCA components, and pairs with the same cell types were retained. The pseudotime ordering of scATAC-seq and scRNA-seq cells have been included in Supplementary Fig. S3A.

Patterns in Figure 3C,D are rather noisy. It is not convincing that there are only two patterns. For example, in C, there are many peaks showing decreasing then increasing pattern, and it is not sure when the open chromatin leads to the increase of gene expression. In D, there are peaks that have increased accessibility after increase of gene expression (in cluster 4 for example). Those may not be called to have synchronized pattern since there is a time lag.

[Response] We agree with the reviewer’s comments; the description of the dynamic gene expression patterns and chromatin accessibility during EPI specification needed to clarify. We have rewritten this part as “To interrogate the correlation between gene expression and chromatin accessibility, we related the DPs to the DEGs, and two main patterns were detected:1) chromatin became accessible first in EPI-A cells, and then the genes were expressed (pattern 1) (Fig. 3C); 2) the scaled values of gene expressions and chromatin accessibility were comparable in EPI-A cells (Fig. 3D) (pattern 2).” (Page 12, lines 189-193). To further delineate the developmental dynamics of gene expression and chromatin accessibility alongside pseudotime ordering of EPI specification, we have paired single cells of scRNA-seq and scATAC-

seq datasets, and the patterns of correlation between gene expression and chromatin accessibility were determined (Supplementary Fig. S3A).

Authors should consider comparing the pseudotime analysis by Monocle 2 with other pseudotime analysis methods such as Slingshot and TSCAN.”

[Response] We have compared the pseudotime trajectory created by Slingshot (Street et al., 2018) and TSCAN (Ji and Ji, 2016) with that of Monocle (v2.18.0) (Qiu et al., 2017), and the same developmental trajectories were observed between this three methods (Fig. 3B and Supplementary Fig. S2A)

Similarly for Figure 3E, why not perform analysis on single-cell level using pseudotime ordering?

[Response] We have performed analysis on single-cell level using pseudotime ordering in Supplementary Fig. S3B.

For Figure 4, similar pseudotime analysis on single-cell level should be done.

[Response] As the reviewer suggested, we have paired single TE cells of scRNA-seq and scATAC-seq datasets and created pseudotime trajectory. Then branch-dependent genes whose expression varied with developmental branching were identified, and their chromatin accessibility and expression levels were visualized (Supplementary Fig. S4D).

“FindAllMarkers" function in Seurat ($p < 0.05$) were raw p-value or adjusted p-value used?

[Response] The adjusted p-values were used.

In the methods section "Comparisons between EPI cells in vitro and in vivo" gene expression values should be scale.

[Response] We have used Seurat (v3.2.2) (Stuart et al., 2019) to remove batch effects from both in vitro and in vivo data, and we used normalized values processed by the “NormalizeData” function according to the tutorial of Seurat. We have added this information in the method section (Page 27, lines 415-416).

In the methods section "Identification of transcription factors that regulates lineage specification": 'The P values of EPI-upregulated genes were divided by the P values of TE-upregulated genes and subsequently log10-transformed.' this does not seem right to me. The pvalues merely reflect the reproducibility of the test and it does not make sense to use p-values as measures of signals. Instead, statistics or fold change should be used in these scenarios.

[Response] As suggested by the reviewer, we have now identified lineage TF based on threshold *P* value and enrichment fold change. EPI-regulated TFs and TE-regulated TFs have been updated, and corresponding figures were replaced in all figures.

Referee #2:

The proposed manuscript described the analysis of the chromatin accessibility of monkey embryos. This is an important resource that has the potential to increase our knowledge in early embryogenesis of embryos. The analysis is enhanced with previously published single cell RNA-seq data. The computational approach and analysis is sound, but mostly restrict on describing TFs, putative targets and the gene ontology terms. Authors do not include any functional validation or comparative analysis, i.e. contrast of results in other organisms. This would have enhanced the study.

[Response] We thank the reviewer for the positive comments. We have revised the manuscript based on the reviewer's suggestions, and the cross-species comparison is included in Supplementary Fig. S6.

Major points:
It is not clear from figure S1C that coembedding found the similar cell types. More analysis should be done, as for example a comparison of cell proportion of the clusters found in scatac and scrna. Another interesting approach would be to correlate the gene accessibility and gene expression of the equivalent cells.

[Response] Thanks for this insightful comment. As suggested, we have added a bar plot showing the percentage of cell types in scATAC-seq and scRNA-seq datasets and box plots to show the chromatin accessibility and gene expression levels of cell markers in each cell type of ATAC-RNA aligned cells. To correlate the gene accessibility and expression of the equivalent cells, we have now paired scATAC-seq and scRNA-seq cells based on a geodesic distance-based pairing approach using FigR, and the combined patterns of chromatin accessibility and gene expressions have been determined (Supplementary Fig. S1C-S1G)

Similarly, it is hard to interpret Fig. S1D. Authors should show the results in the co-embedding space.

[Response] We have provided the new figures showing the results in the co-embedding space in Supplementary Fig. S1G.

Altogether authors should give more details on how the scRNA/scATAC integration is done. This could also include a short results in the results section (page 7).

[Response] We thank the reviewer for raising this point. As suggested, we have described more details on integrating scRNA-seq and scATAC-seq datasets in the results section (page 7, lines 96-106) and methods (page 29, lines 454-476). Furthermore, a histogram plot was provided to show the distribution of prediction scores in annotated label transfer based on CCA, reflecting the integration quality

(Supplementary Fig. S1D).

Reference

Ji, Z., and Ji, H. (2016). TSCAN: Pseudo-time reconstruction and evaluation in single-cell RNA-seq analysis. Nucleic Acids Res *44*, e117. 10.1093/nar/gkw430.

Kartha, V.K., Duarte, F.M., Hu, Y., Ma, S., Chew, J.G., Lareau, C.A., Earl, A., Burkett, Z.D., Kohlway, A.S., Lebofsky, R., and Buenrostro, J.D. (2022). Functional inference of gene regulation using single-cell multi-omics. Cell Genom *2*. 10.1016/j.xgen.2022.100166.

Qiu, X., Hill, A., Packer, J., Lin, D., Ma, Y.A., and Trapnell, C. (2017). Single-cell mRNA quantification and differential analysis with Census. Nat Methods *14*, 309-315. 10.1038/nmeth.4150.

Street, K., Risso, D., Fletcher, R.B., Das, D., Ngai, J., Yosef, N., Purdom, E., and Dudoit, S. (2018). Slingshot: cell lineage and pseudotime inference for single-cell transcriptomics. BMC Genomics *19*, 477. 10.1186/s12864-018-4772-0.

Stuart, T., Butler, A., Hoffman, P., Hafemeister, C., Papalexi, E., Mauck, W.M., 3rd, Hao, Y., Stoeckius, M., Smibert, P., and Satija, R. (2019). Comprehensive Integration of Single-Cell Data. Cell *177*, 1888-1902 e1821. 10.1016/j.cell.2019.05.031.
