# Supplementary figures and images for: Developmental dynamics of chromatin accessibility during post-implantation development of monkey embryos
Source: Gigascience. 2023 May 25;12:giad038. doi: 10.1093/gigascience/giad038 (PMC10209733; doi:10.1093/gigascience/giad038)

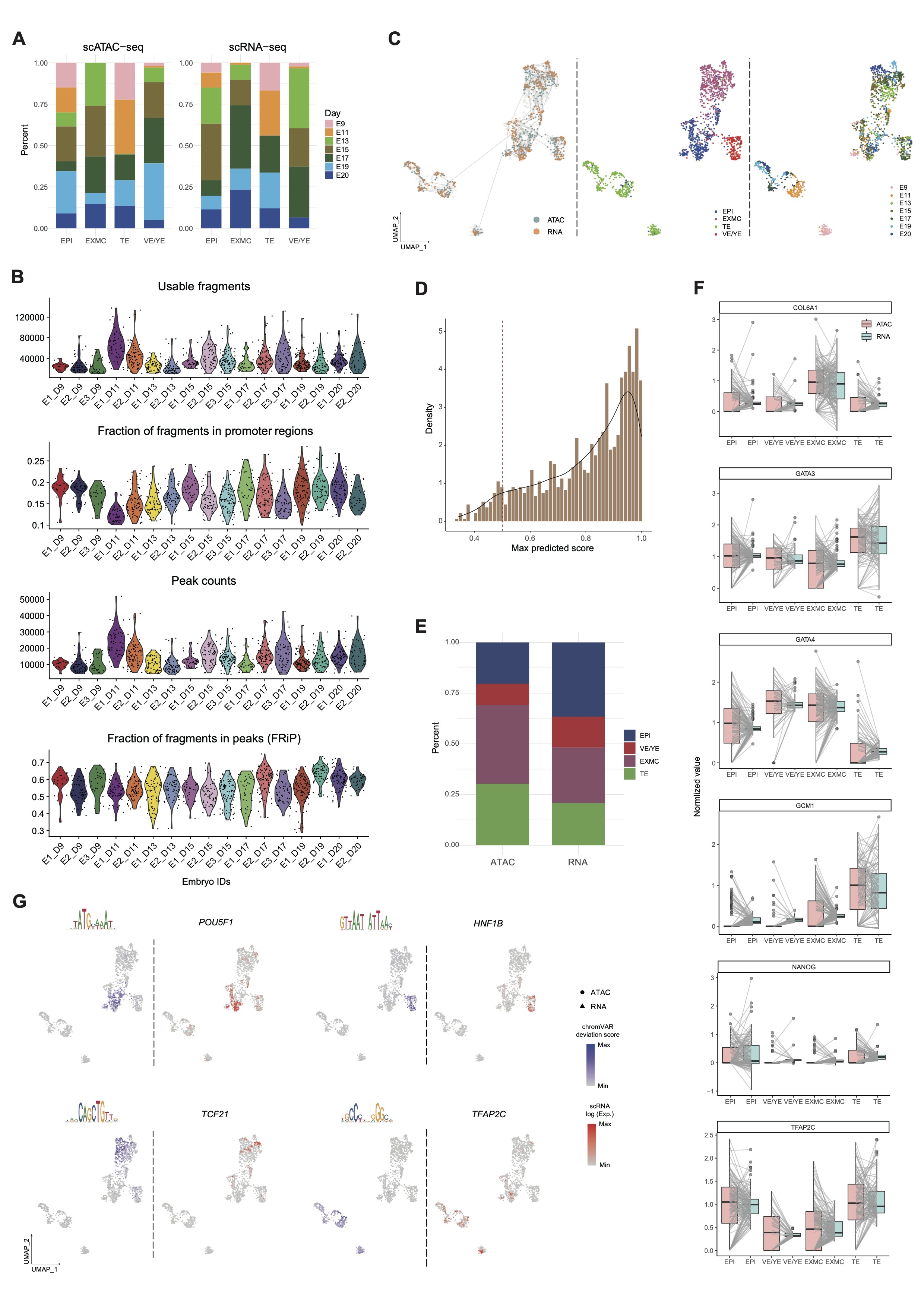

Supplement: giad038_Supplemental_Figures_and_Tables [file giad038_supplemental_figures_and_tables.zip › figS1.jpg]

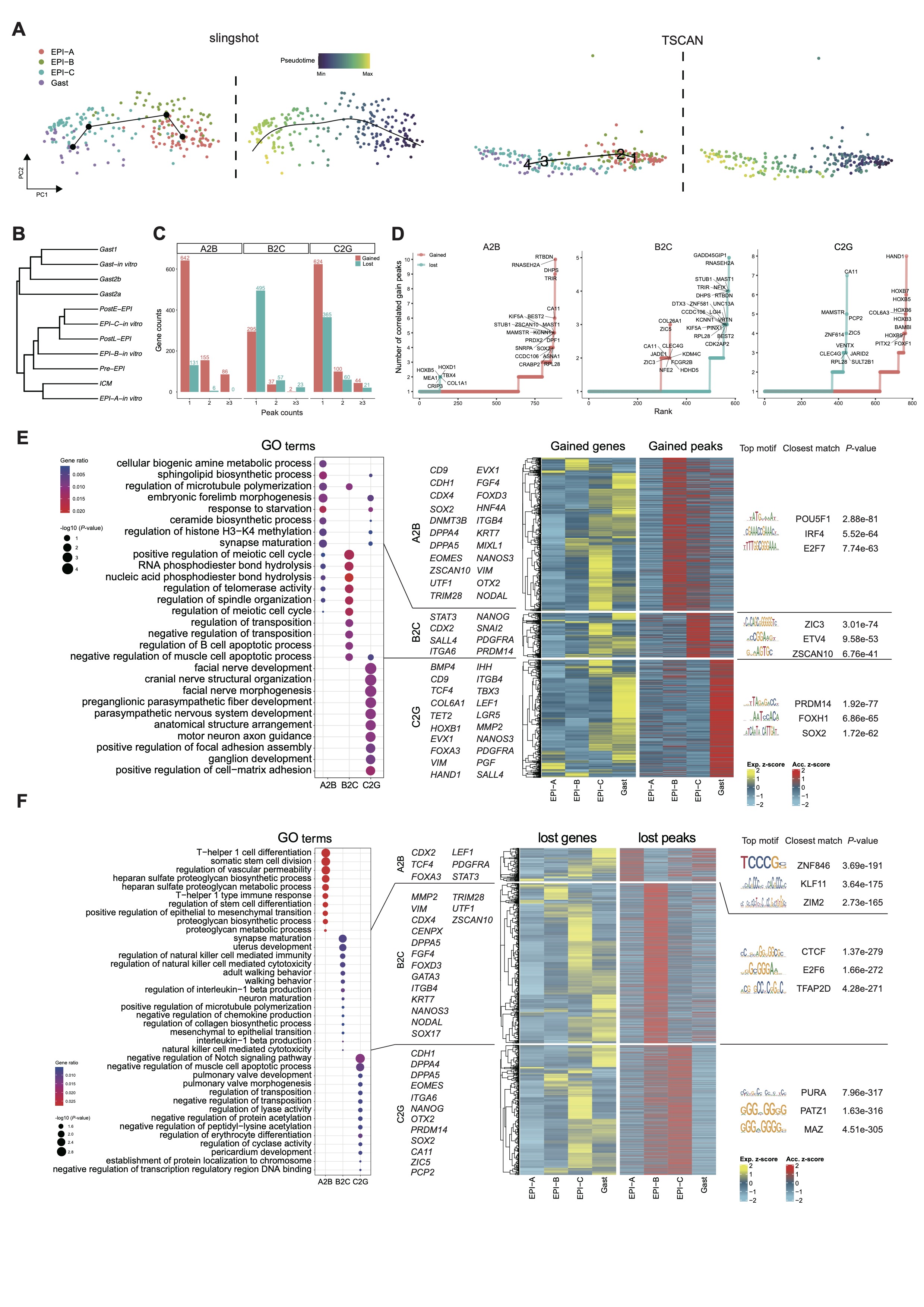

Supplement: giad038_Supplemental_Figures_and_Tables [file giad038_supplemental_figures_and_tables.zip › figS2.jpg]

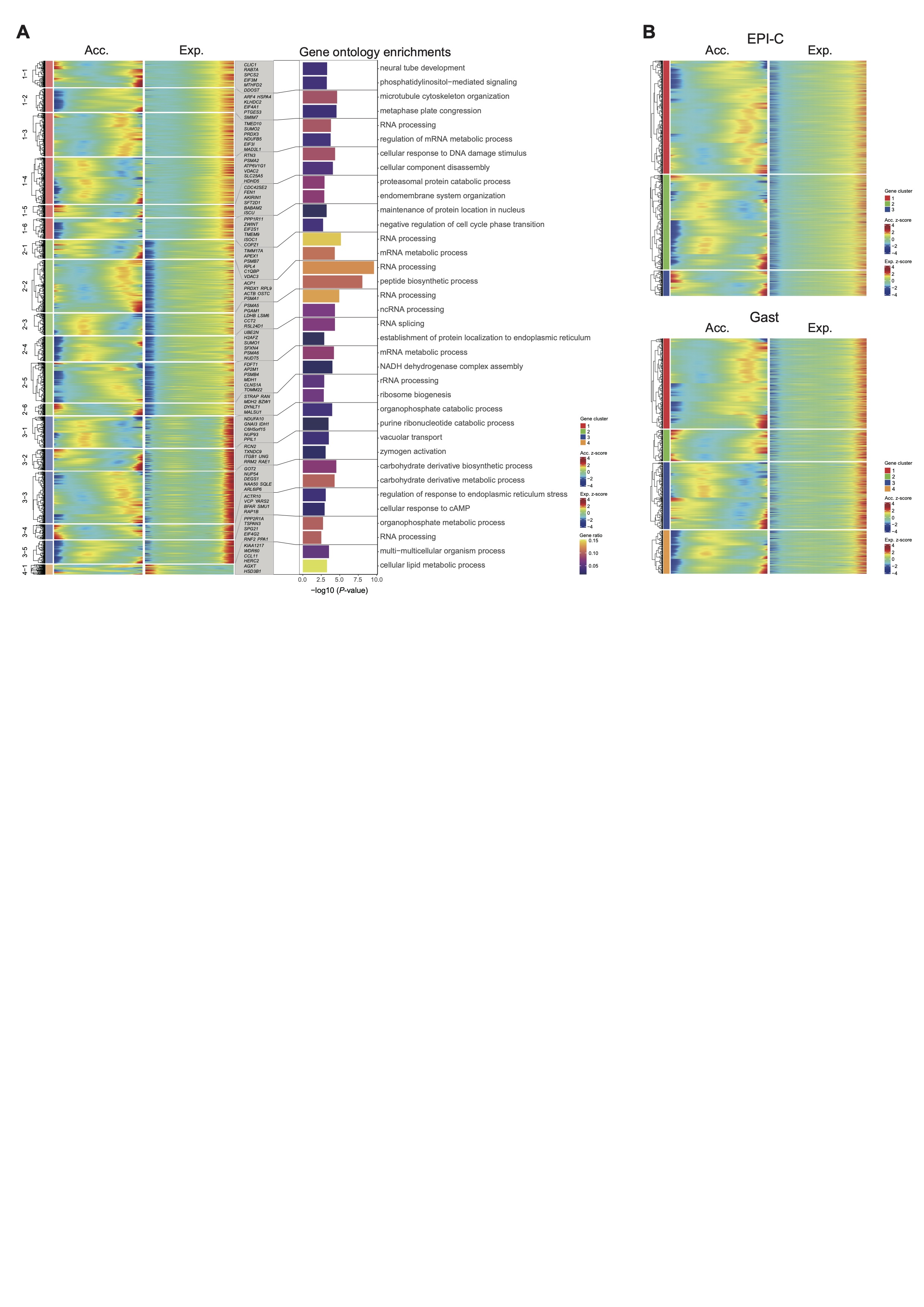

Supplement: giad038_Supplemental_Figures_and_Tables [file giad038_supplemental_figures_and_tables.zip › figS3.jpg]

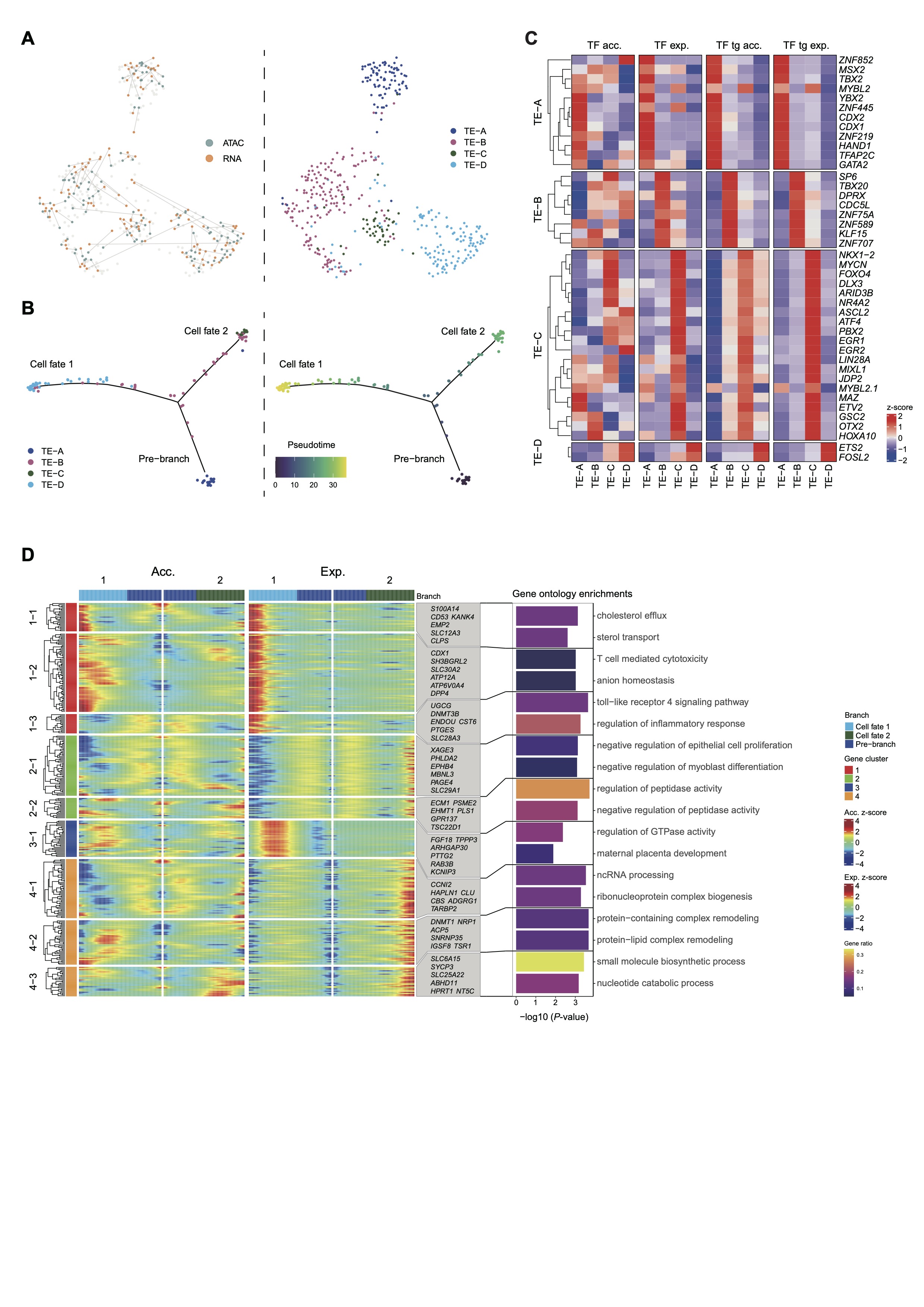

Supplement: giad038_Supplemental_Figures_and_Tables [file giad038_supplemental_figures_and_tables.zip › figS4.jpg]

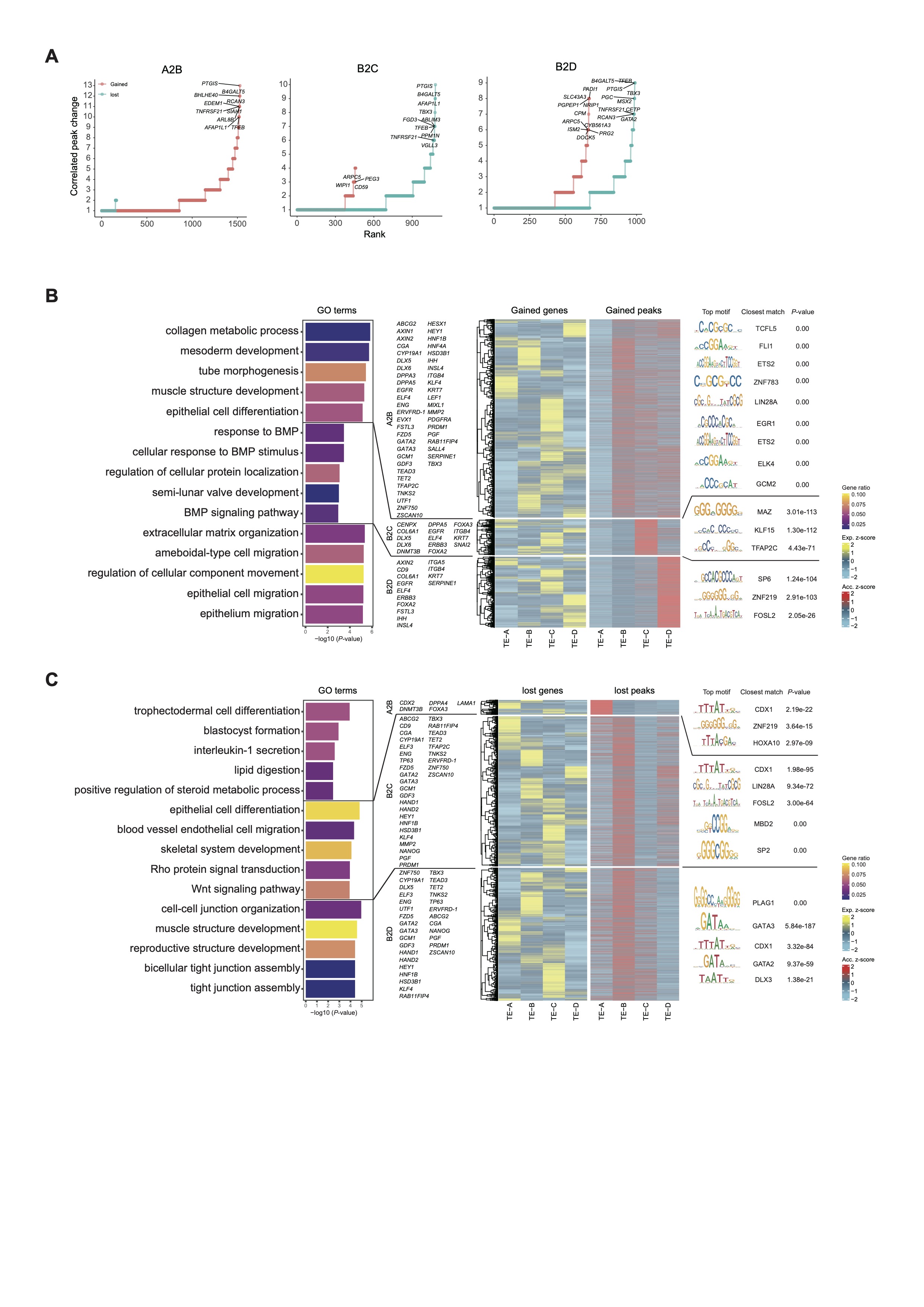

Supplement: giad038_Supplemental_Figures_and_Tables [file giad038_supplemental_figures_and_tables.zip › figS5.jpg]

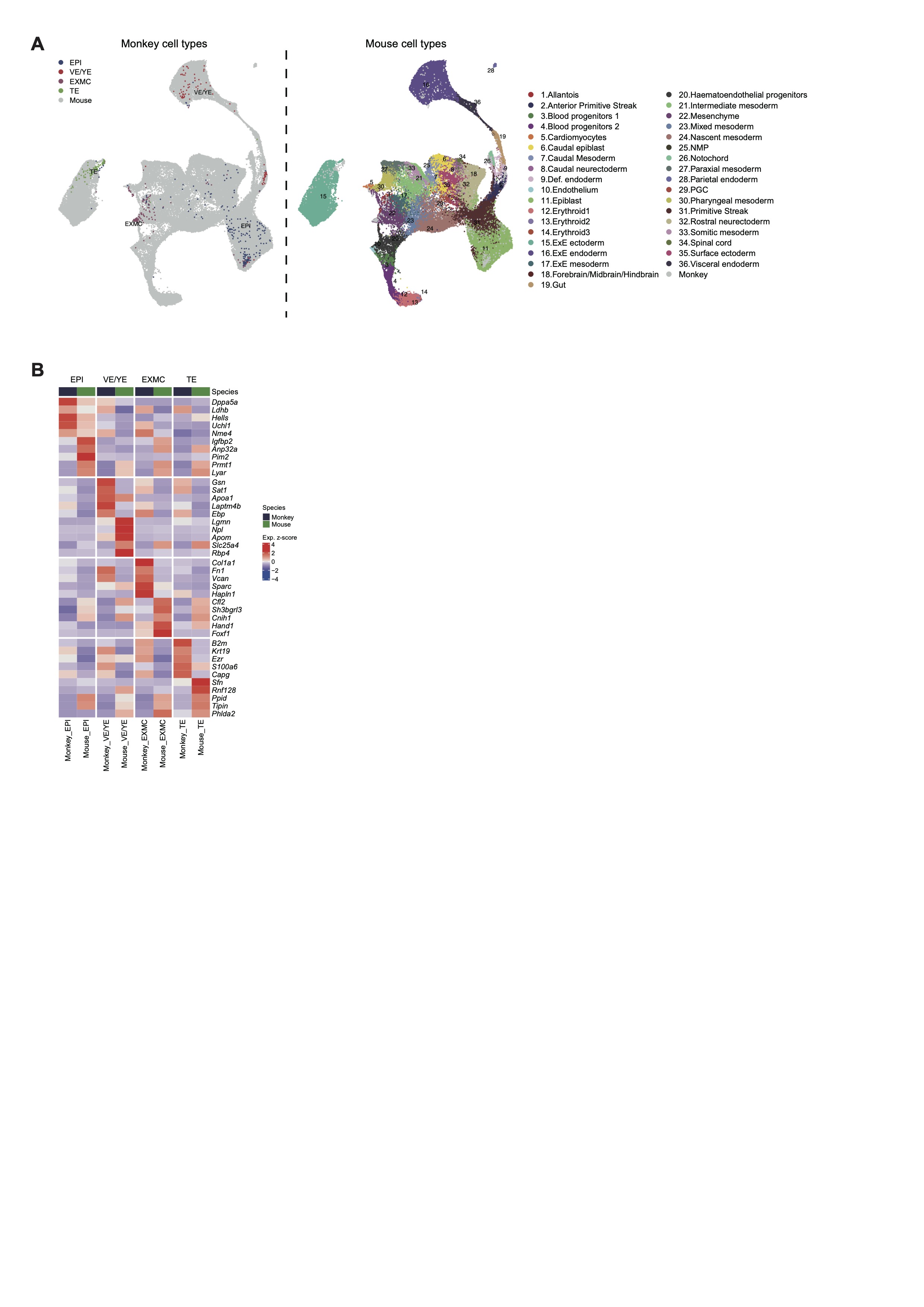

Supplement: giad038_Supplemental_Figures_and_Tables [file giad038_supplemental_figures_and_tables.zip › figS6.jpg]

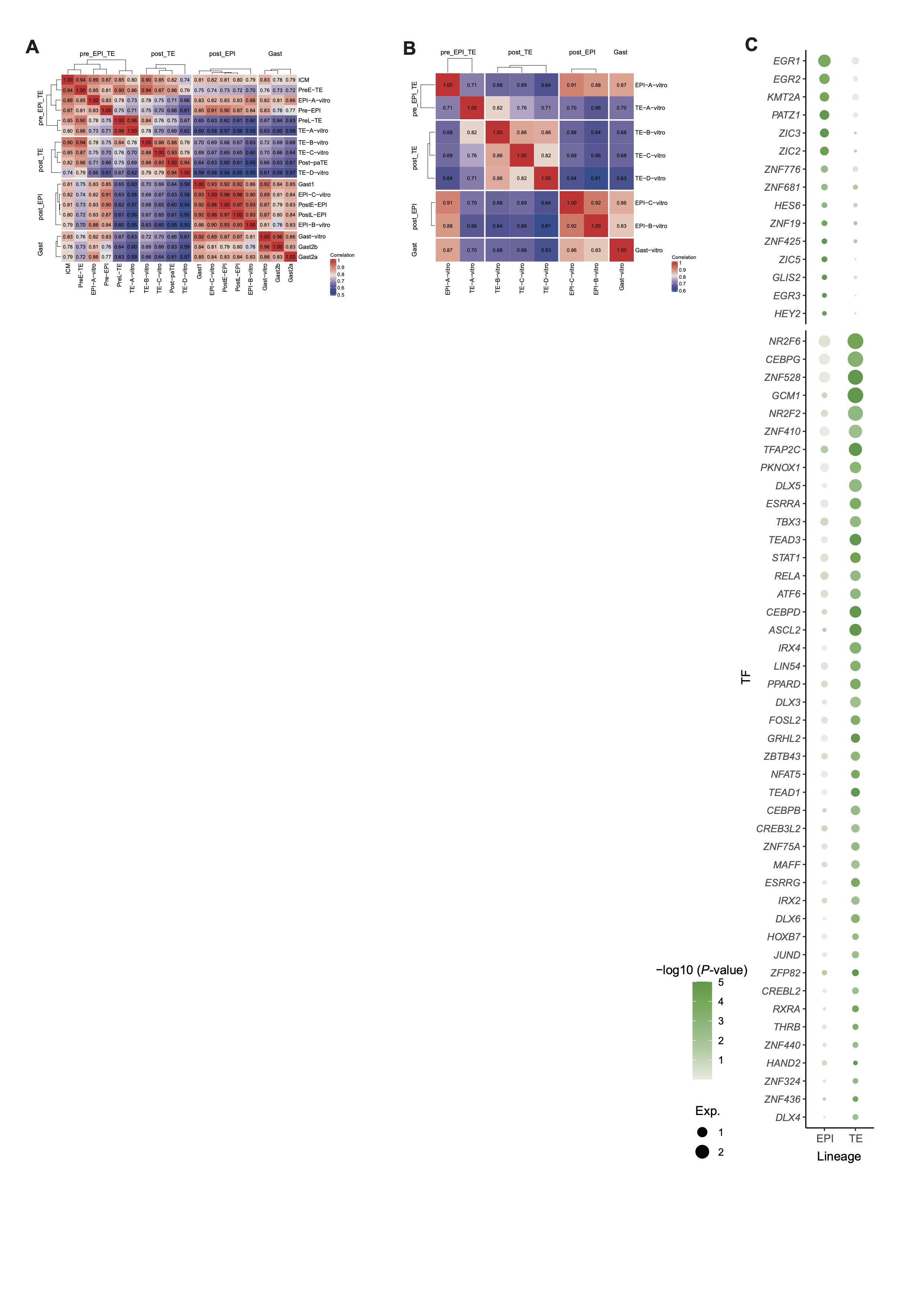

Supplement: giad038_Supplemental_Figures_and_Tables [file giad038_supplemental_figures_and_tables.zip › figS7.jpg]
