# Supplementary material for: Developmental dynamics of chromatin accessibility during post-implantation development of monkey embryos
Source: Gigascience. 2023 May 25;12:giad038. doi: 10.1093/gigascience/giad038 (PMC10209733; doi:10.1093/gigascience/giad038)
Supplement: giad038_Response_to_Reviewer_Comments_Original_Submission [file giad038_response_to_reviewer_comments_original_submission.pdf]

Author's Response To Reviewer Comments

Close

Dear Dr. Hongling,

I am attaching our revised manuscript (GIGA-D-22-00278) titled "Developmental dynamics of chromatin accessibility during post-implantation development of monkey embryos". We thank you and the reviewers for the positive comments and suggestions, which helped improve our manuscript immensely. We have provided a detailed point-by-point response and revised the manuscript to address the remaining concerns.

If you have any questions, please do not hesitate to contact us.

Tao Tan
State Key Laboratory of Primate Biomedical Research
Institute of Primate Translational Medicine
Kunming University of Science and Technology

Our responses to the reviewers' comments

Referee #1:

The authors present a scATAC-seq dataset of monkey embryo development. A major limitation of the analysis is that the authors seem to pool cells within each cell type instead directly doing analysis on a single-cell level, which largely loses the power and resolution of single-cell analysis. Below are my specific comments:

[Response] We greatly thank the reviewer for the constructive comments and agree that analysis on the single-cell level is necessary. We have added the single-cell level analysis in Supplementary Fig. S1C-1G, Fig. 3A-3B, Supplementary Fig. S2A, Supplementary Fig. S3, Supplementary Fig. S4A, S4B, and S4D based on the reviewer's suggestion.

Fig 1d: What is each row? Is each row showing one DP and its corresponding gene, or one DEG and its corresponding peak? Or are there duplicated genes or peaks in the plot?

[Response] We have added the descriptions in the figure legend of Fig 1D to clarify this ambiguity. Each row shows one DP with its corresponding gene, whereas some genes are duplicated in a row cluster as multiple peaks link to one gene. (Page 50, lines 880-882).

For the single-cell analysis, the authors claim that ' which implies that there is a time lag between gene expression and chromatin accessibility during EPI cell specification'. However, this may not be true biology since the scRNA and scATAC were computationally aligned. The time lag could be introduced by the computational alignment.

[Response] We thank the reviewer for raising this point. In our bioinformatic analysis approach, similar clusters were aligned between scRNA-seq and scATAC-seq datasets based on the top 2,000 variable genes, and high consistency between matched clusters was observed (also see Methods) (Page 29, lines 457-462), implying similar clusters of scRNA-seq and scATAC-seq datasets were well aligned (Fig. 3A). Based on this observation, the asynchrony features instead of the congruent features in the aligned clusters are plausible. To further clarify the possibility that the computational alignment could introduce the time lag, we have aligned single ATAC and RNA cells in the revised manuscript based on a geodesic distance-based pairing approach using "FigR" which can reduce the deviation caused by cell cluster mean value to a certain extent (Kartha et al., 2022) (Fig.3A). Notably, a time lag between gene expression and chromatin

accessibility during EPI cell specification was also observed (Supplementary Fig. S3A). We also have toned down this claim as “which implies an inconsistency between gene expression and chromatin accessibility during EPI cell specification” in the revised manuscript (Page 12, lines 184-185).

For the pattern analysis in Figure 3C,D, why not use the pseudotime ordering and perform the analysis on a single-cell level, instead of the current analysis that pools cells with the same cell type

[Response] We have aligned the nearest scATAC and scRNA cells by the “pairCells” function in the R package FigR (Kartha et al., 2022) based on co-embedding PCA components, and pairs with the same cell types were retained. The pseudotime ordering of scATAC-seq and scRNA-seq cells have been included in Supplementary Fig. S3A.

Patterns in Figure 3C,D are rather noisy. It is not convincing that there are only two patterns. For example, in C, there are many peaks showing decreasing then increasing pattern, and it is not sure when the open chromatin leads to the increase of gene expression. In D, there are peaks that have increased accessibility after increase of gene expression (in cluster 4 for example). Those may not be called to have synchronized pattern since there is a time lag.

[Response] We agree with the reviewer’s comments; the description of the dynamic gene expression patterns and chromatin accessibility during EPI specification needed to clarify. We have rewritten this part as “To interrogate the correlation between gene expression and chromatin accessibility, we related the DPs to the DEGs, and two main patterns were detected:1) chromatin became accessible first in EPI-A cells, and then the genes were expressed (pattern 1) (Fig. 3C); 2) the scaled values of gene expressions and chromatin accessibility were comparable in EPI-A cells (Fig. 3D) (pattern 2).” (Page 12, lines 189-193). To further delineate the developmental dynamics of gene expression and chromatin accessibility alongside pseudotime ordering of EPI specification, we have paired single cells of scRNA-seq and scATAC-seq datasets, and the patterns of correlation between gene expression and chromatin accessibility were determined (Supplementary Fig. S3A).

Authors should consider comparing the pseudotime analysis by Monocle 2 with other pseudotime analysis methods such as Slingshot and TSCAN.”

[Response] We have compared the pseudotime trajectory created by Slingshot (Street et al., 2018) and TSCAN (Ji and Ji, 2016) with that of Monocle (v2.18.0) (Qiu et al., 2017), and the same developmental trajectories were observed between this three methods (Fig. 3B and Supplementary Fig. S2A)

Similarly for Figure 3E, why not perform analysis on single-cell level using pseudotime ordering?

[Response] We have performed analysis on single-cell level using pseudotime ordering in Supplementary Fig. S3B.

For Figure 4, similar pseudotime analysis on single-cell level should be done.

[Response] As the reviewer suggested, we have paired single TE cells of scRNA-seq and scATAC-seq datasets and created pseudotime trajectory. Then branch-dependent genes whose expression varied with developmental branching were identified, and their chromatin accessibility and expression levels were visualized (Supplementary Fig. S4D).

“FindAllMarkers" function in Seurat ($p < 0.05$) were raw p-value or adjusted p-value used?

[Response] The adjusted p-values were used.

In the methods section "Comparisons between EPI cells in vitro and in vivo" gene expression values should be scale.

[Response] We have used Seurat (v3.2.2) (Stuart et al., 2019) to remove batch effects from both in vitro and in vivo data, and we used normalized values processed by the “NormalizeData” function according to

the tutorial of Seurat. We have added this information in the method section (Page 27, lines 415-416).

In the methods section "Identification of transcription factors that regulates lineage specification": 'The P values of EPI-upregulated genes were divided by the P values of TE-upregulated genes and subsequently log10-transformed.' this does not seem right to me. The pvalues merely reflect the reproducibility of the test and it does not make sense to use p-values as measures of signals. Instead, statistics or fold change should be used in these scenarios.

[Response] As suggested by the reviewer, we have now identified lineage TF based on threshold P value and enrichment fold change. EPI-regulated TFs and TE-regulated TFs have been updated, and corresponding figures were replaced in all figures.

Referee #2:

The proposed manuscript described the analysis of the chromatin accessibility of monkey embryos. This is an important resource that has the potential to increase our knowledge in early embryogenesis of embryos. The analysis is enhanced with previously published single cell RNA-seq data. The computational approach and analysis is sound, but mostly restrict on describing TFs, putative targets and the gene ontology terms. Authors do not include any functional validation or comparative analysis, i.e. contrast of results in other organisms. This would have enhanced the study.

[Response] We thank the reviewer for the positive comments. We have revised the manuscript based on the reviewer's suggestions, and the cross-species comparison is included in Supplementary Fig. S6.

Major points:
It is not clear from figure S1C that coembedding found the similar cell types. More analysis should be done, as for example a comparison of cell proportion of the clusters found in scatac and scrna. Another interesting approach would be to correlate the gene accessibility and gene expression of the equivalent cells.

[Response] Thanks for this insightful comment. As suggested, we have added a bar plot showing the percentage of cell types in scATAC-seq and scRNA-seq datasets and box plots to show the chromatin accessibility and gene expression levels of cell markers in each cell type of ATAC-RNA aligned cells. To correlate the gene accessibility and expression of the equivalent cells, we have now paired scATAC-seq and scRNA-seq cells based on a geodesic distance-based pairing approach using FigR, and the combined patterns of chromatin accessibility and gene expressions have been determined (Supplementary Fig. S1C-S1G)

Similarly, it is hard to interpret Fig. S1D. Authors should show the results in the co-embedding space.

[Response] We have provided the new figures showing the results in the co-embedding space in Supplementary Fig. S1G.

Altogether authors should give more details on how the scRNA/scATAC integration is done. This could also include a short results in the results section (page 7).

[Response] We thank the reviewer for raising this point. As suggested, we have described more details on integrating scRNA-seq and scATAC-seq datasets in the results section (page 7, lines 96-106) and methods (page 29, lines 454-476). Furthermore, a histogram plot was provided to show the distribution of prediction scores in annotated label transfer based on CCA, reflecting the integration quality (Supplementary Fig. S1D).

Reference

Ji, Z., and Ji, H. (2016). TSCAN: Pseudo-time reconstruction and evaluation in single-cell RNA-seq analysis. Nucleic Acids Res 44, e117. 10.1093/nar/gkw430.
Kartha, V.K., Duarte, F.M., Hu, Y., Ma, S., Chew, J.G., Lareau, C.A., Earl, A., Burkett, Z.D., Kohlway, A.S.,

Lebofsky, R., and Buenrostro, J.D. (2022). Functional inference of gene regulation using single-cell multi-omics. Cell Genom 2. 10.1016/j.xgen.2022.100166.
Qiu, X., Hill, A., Packer, J., Lin, D., Ma, Y.A., and Trapnell, C. (2017). Single-cell mRNA quantification and differential analysis with Census. Nat Methods 14, 309-315. 10.1038/nmeth.4150.
Street, K., Risso, D., Fletcher, R.B., Das, D., Ngai, J., Yosef, N., Purdom, E., and Dudoit, S. (2018). Slingshot: cell lineage and pseudotime inference for single-cell transcriptomics. BMC Genomics 19, 477. 10.1186/s12864-018-4772-0.
Stuart, T., Butler, A., Hoffman, P., Hafemeister, C., Papalexi, E., Mauck, W.M., 3rd, Hao, Y., Stoeckius, M., Smibert, P., and Satija, R. (2019). Comprehensive Integration of Single-Cell Data. Cell 177, 1888-1902 e1821. 10.1016/j.cell.2019.05.031.

Close
