# Supplementary material for: Developmental dynamics of chromatin accessibility during post-implantation development of monkey embryos
Source: Gigascience. 2023 May 25;12:giad038. doi: 10.1093/gigascience/giad038 (PMC10209733; doi:10.1093/gigascience/giad038)
Supplement: giad038_Reviewer_1_Report_Original_Submission — Zhicheng Ji -- 11/12/2022 Reviewed [file giad038_reviewer_1_report_original_submission.pdf]

Reviewer Report

Title: Developmental dynamics of chromatin accessibility during post-implantation development of monkey embryos

Version: Original Submission **Date:** 11/12/2022

Reviewer name: Zhicheng Ji

Reviewer Comments to Author:

The authors present a scATAC-seq dataset of monkey embryo development. A major limitation of the analysis is that the authors seem to pool cells within each cell type instead directly doing analysis on a single-cell level, which largely loses the power and resolution of single-cell analysis. Below are my specific comments:

1. Fig 1d: What is each row? Is each row showing one DP and its corresponding gene, or one DEG and its corresponding peak? Or are there duplicated genes or peaks in the plot?
2. For the single-cell analysis, the authors claim that ' which implies that there is a time lag between gene expression and chromatin accessibility during EPI cell specification'. However, this may not be true biology since the scRNA and scATAC were computationally aligned. The time lag could be introduced by the computational alignment.
3. For the pattern analysis in Figure 3C,D, why not use the pseudotime ordering and perform the analysis on a single-cell level, instead of the current analysis that pools cells with the same cell type?
4. Patterns in Figure 3C,D are rather noisy. It is not convincing that there are only two patterns. For example, in C, there are many peaks showing decreasing then increasing pattern, and it is not sure when the open chromatin leads to the increase of gene expression. In D, there are peaks that have increased accessibility after increase of gene expression (in cluster 4 for example). Those may not be called to have synchronized pattern since there is a time lag.
5. Authors should consider comparing the pseudotime analysis by Monocle 2 with other pseudotime analysis methods such as Slingshot and TSCAN.
6. Similarly for Figure 3E, why not perform analysis on single-cell level using pseudotime ordering?
7. For Figure 4, similar pseudotime analysis on single-cell level should be done.
8. "FindAllMarkers" function in Seurat ($p < 0.05$)" were raw p-value or adjusted p-value used?
9. In the methods section "Comparisons between EPI cells in vitro and in vivo" gene expression values should be scaled to have mean 0 and sd 1 across cells within each gene to remove the gene-specific effect. correlation should be calculated on the scaled values. Otherwise the correlation largely reflects housekeeping gene effects (i.e. some genes are always highly expressed)
10. In the methods section "Identification of transcription factors that regulates lineage specification": 'The P values of EPI-upregulated genes were divided by the P values of TE-upregulated genes and subsequently log10-transformed.' this does not seem right to me. The pvalues merely reflect the reproducibility of the test and it does not make sense to use p-values as measures of signals. Instead, statistics or fold change should be used in these scenarios.

Methods

Are the methods appropriate to the aims of the study, are they well described, and are necessary controls included? Choose an item.

Conclusions

Are the conclusions adequately supported by the data shown? Choose an item.

Reporting Standards

Does the manuscript adhere to the journal's guidelines on minimum standards of reporting? Choose an item.

Choose an item.

Statistics

Are you able to assess all statistics in the manuscript, including the appropriateness of statistical tests used? Choose an item.

Quality of Written English

Please indicate the quality of language in the manuscript: Choose an item.

Declaration of Competing Interests

Please complete a declaration of competing interests, considering the following questions:

- Have you in the past five years received reimbursements, fees, funding, or salary from an organisation that may in any way gain or lose financially from the publication of this manuscript, either now or in the future?
- Do you hold any stocks or shares in an organisation that may in any way gain or lose financially from the publication of this manuscript, either now or in the future?
- Do you hold or are you currently applying for any patents relating to the content of the manuscript?
- Have you received reimbursements, fees, funding, or salary from an organization that holds or has applied for patents relating to the content of the manuscript?
- Do you have any other financial competing interests?
- Do you have any non-financial competing interests in relation to this paper?

If you can answer no to all of the above, write 'I declare that I have no competing interests' below. If your reply is yes to any, please give details below.

I declare that I have no competing interests

I agree to the open peer review policy of the journal. I understand that my name will be included on my report to the authors and, if the manuscript is accepted for publication, my named report including any attachments I upload will be posted on the website along with the authors' responses. I agree for my report to be made available under an Open Access Creative Commons CC-BY license (http://creativecommons.org/licenses/by/4.0/). I understand that any comments which I do not wish to be included in my named report can be included as confidential comments to the editors, which will not be published.

Choose an item.

To further support our reviewers, we have joined with Publons, where you can gain additional credit to further highlight your hard work (see: https://publons.com/journal/530/gigascience). On publication of this paper, your review will be automatically added to Publons, you can then choose whether or not to claim your Publons credit. I understand this statement.

Yes Choose an item.
